# Active Essential Oils and Their Components in Use against Neglected Diseases and Arboviruses

**DOI:** 10.1155/2019/6587150

**Published:** 2019-02-07

**Authors:** Emanuela Coutinho Luna, Isadora Silva Luna, Luciana Scotti, Alex France M. Monteiro, Marcus Tullius Scotti, Ricardo Olímpio de Moura, Rodrigo Santos Aquino de Araújo, Kadja Luana Chagas Monteiro, Thiago Mendonça de Aquino, Frederico Fávaro Ribeiro, Francisco Jaime Bezerra Mendonça

**Affiliations:** ^1^Laboratório de Síntese e Vetorização de Moléculas, Departamento de Ciências Biológicas, Universidade Estadual da Paraíba, 58071-160 João Pessoa-PB, Brazil; ^2^Teaching and Research Management-University Hospital, Federal University of Paraíba, Campus I, João Pessoa-PB, Brazil; ^3^Postgraduate Program in Natural Products and Synthetic Bioactive, Federal University of Paraíba, Campus I, João Pessoa-PB, Brazil; ^4^Laboratory of Medicinal Chemistry, Nursing and Pharmacy School, Federal University of Alagoas, 57072-900 Maceió-AL, Brazil; ^5^Chemistry and Biotechnology Institute, Federal University of Alagoas, Maceió, Brazil

## Abstract

The term *neglected diseases* refers to a group of infections caused by various classes of pathogens, including protozoa, viruses, bacteria, and helminths, most often affecting impoverished populations without adequate sanitation living in close contact with infectious vectors and domestic animals. The fact that these diseases were historically not considered priorities for pharmaceutical companies made the available treatments options obsolete, precarious, outdated, and in some cases nonexistent. The use of plants for medicinal, religious, and cosmetic purposes has a history dating back to the emergence of humanity. One of the principal fractions of chemical substances found in plants are essential oils (EOs). EOs consist of a mixture of volatile and hydrophobic secondary metabolites with marked odors, composed primarily of terpenes and phenylpropanoids. They have great commercial value and were widely used in traditional medicine, by phytotherapy practitioners, and in public health services for the treatment of several conditions, including neglected diseases. In addition to the recognized cytoprotective and antioxidative activities of many of these compounds, larvicidal, insecticidal, and antiparasitic activities have been associated with the induction of oxidative stress in parasites, increasing levels of nitric oxide in the infected host, reducing parasite resistance to reactive oxygen species, and increasing lipid peroxidation, ultimately leading to serious damage to cell membranes. The hydrophobicity of these compounds also allows them to cross the membranes of parasites as well as the blood-brain barrier, collaborating in combat at the second stage of several of these infections. Based on these considerations, the aim of this review was to present an update of the potential of EOs, their fractions, and their chemical constituents, against some neglected diseases, including American and African trypanosomiasis, leishmaniasis, and arboviruses, specially dengue.

## 1. Introduction

Neglected diseases (ND) are a group of infections caused by various classes of infectious and parasitic pathogens, including protozoa, viruses, bacteria, and helminths. They most often affect low-income populations, with higher prevalence in tropical and subtropical countries. According to the World Health Organization (WHO), they are a set of 17 diseases, including leishmaniasis, African and American trypanosomiasis, leprosy, tuberculosis, leptospirosis, onchocerciasis, and schistosomiasis, affecting more than one billion people around the world and considered a threat to public health in 149 countries [1, 2].

The fact that these diseases are not considered priorities by large medical and pharmaceutical companies results in low investment in research and development of new drugs, causing the few available treatment options to become obsolete, precarious, outdated, and even in some cases nonexistent [3].

These diseases are known for the lack of attention paid to them by political, social, and health entities. The worsening of social and health conditions in a great part of the world's population has accelerated in recent years according to studies providing data regarding current social policies [4–6].

According to some studies in Latin American countries, Brazil has the highest rate of neglected diseases, and a large part of the Brazilian population both above and below the poverty line suffers from some of these diseases [7–9].

Several neglected diseases are arboviruses, that is, viral diseases transmitted through the saliva of contaminated arthropods [10]. Thus, they spread with worrisome epidemiological speed because of human exposure to the insect vectors [11, 12]. In some cases, a single vector can transmit more than one disease, as is the case of the vector *Aedes aegypti* that may transmit dengue, Zika, and chikungunya.

According to some authors [13, 14], 80% of the Brazilian population lives in urban areas. Demographic expansion and climatic conditions create an attractive environment for the reproduction of arbovirus vectors, rapidly affecting a large part of the population living in these urban centers [13]. Due to the absence of many natural predators, disease-bearing arthropods reproduce more easily in urban centers.

The use of plants for medicinal, religious, and cosmetic purposes dates back to the emergence of humanity. This historical interest promoted the development of several areas of inquiry invested in understanding and unraveling the potential of substances produced by plants as a result of their adaptive evolution [15, 16].

One of the principal fractions of chemical substances found in plants are essential oils (EOs). EOs consist primarily of a mixture of volatile hydrophobic secondary metabolites with marked odors and great commercial value; EOs may be thought of as fingerprints of the plants where they are found [17, 18]. They include aromatic alcohols, acids, esters, phenolics, ketones, aldehydes, and hydrocarbons. In plants, EOs act as protective agents against predators and attractants for pollinators [19, 20].

The main chemical compounds found in EOs are terpenoids and phenylpropanoids [21, 22] that are widely used as bioactive molecules in biology, agronomy, medicine, and pharmaceutical sciences. Among the medical-pharmaceutical activities of these compounds are antitumor, antihelmintic, and larvicidal [23–25] and insecticidal [26–32] activities, as well as activity against arbovirus vectors [33, 34].

Despite the recognized cytoprotective and antioxidative activities of these compounds, in many cases larvicidal, insecticidal, and antiparasitic activities are associated with the induction of oxidative stress in the parasite, increasing the level of nitric oxide production by the infected host, reducing parasite resistance to reactive oxygen species, and generating high levels of free radicals and increasing lipid peroxidation, ultimately leading to serious damage to cell membranes and the killing of parasites [35–40].

Based on these considerations, in this review an update of the potential of EOs, their fractions, and their components that are active against some neglected diseases, including American and African trypanosomiasis, leishmaniasis, and arboviruses, specially dengue is presented.

To compose the database, the authors did a systematical search in the following databases: PubMed, Web of Science, Medline, SciELO, Cochrane Library, and NAPRALERT. The following search terms were combined and screened: essential oils, name of the neglected disease, and/or the parasite name. No language restrictions were applied. The period considered was the last 10 years, without however disregarding some articles of revision, and some classical literatures were considered important.

### 1.1. Essentials Oils Active against *Trypanosoma cruzi*

Chagas disease (CD), also known as American trypanosomiasis, is a potentially life-threatening disease caused by the protozoan *Trypanosoma cruzi* [41]. According to the World Health Organization, CD affects about 7 million people worldwide and is endemic in 21 Latin American countries. It is estimated that CD leads to more than 7000 deaths per year and there are about 25 million people at risk for infection. Transmission to humans usually occurs through the excrement of infected vector insects (haematophagous triatomines) that normally become active at night, feeding on mammalian blood by biting exposed skin and defecating close to the bite. Transmission can also occur through transfusion of infected blood, congenital transmission, and (less frequently) organ transplantation or laboratory accidents [42].

Although it is an infectious disease with more than 100 years since its discovery, the morbidity and mortality associated with CD remains high. This may be attributed to several factors, including the absence of effective vaccines and/or medical treatments. Currently, only two drugs are available for CD treatment: benznidazole (BZ) and nifurtimox (NFX). Unfortunately, these drugs carry high toxicity and cause severe side effects that often interrupt treatment [43]. Therefore, there is a need for new therapeutic alternatives for CD that are more effective and less toxic for both acute and chronic phases.

Therefore, the use of natural products is an important strategy to identify new trypanocidal compounds, and the literature already described promising studies with the use of EOs, found in some species of plants in many tropical countries [44].

Over the last two decades, the trypanocidal activity of approximately 400 plant species has been analyzed; however, few reports have assessed the trypanocidal activity of EOs [45, 46].

The literature described that EOs have been used since the Middle Ages by Arabs for their antiseptic, bactericidal, virucidal, and fungicidal properties. They are also used for embalming and preservation of foods and as drugs with antimicrobial, analgesic, sedative, anti-inflammatory, spasmolytic, and local anesthesic properties [47]. However, little was known about their antiparasitic applications, with the first reports appearing only at the end of the 20^th^ century and beginning of the 21^st^ century. Chagas disease was no different, and only in recent decades has there been an increase in the number of publications describing the use of EOs and/or their constituents with inhibitory activity against trypanosomatid protozoa [48–50].

Holetz and coworkers produced one of the first reports of the effects of the essential oil of *Ocimum gratissimum* on *Herpetomonas samuelpessoai*, a nonpathogenic trypanosomatid that shares important antigens with *T. cruzi* and can be easily cultured. This trypanosomatid also induced humoral and cell-mediated immune responses, showing itself to be a good model for study of the biology of trypanosomatids and a suitable model for screening new trypanocidal drugs. The EO of *Ocimum gratissimum* inhibited protozoal growth in a dose-dependent manner with IC_50_ values between 100 and 91 *μ*g/mL, at concentrations from 20 to 250 *μ*g/mL [51, 52].

**Figure 1:**
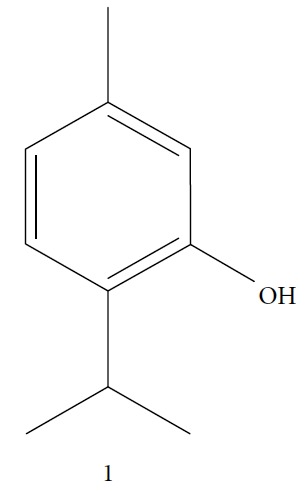
Chemical structure of thymol (**1**) - the main component of *T. vulgaris* EO.

In 2006, Santoro and colleagues investigated the effects of EOs from *Origanum vulgare* and *Thymus vulgaris* on growth and ultrastructure of evolutionary forms of *T. cruzi*. EO from oregano showed an IC_50_/24 h value of 175 *μ*g/mL against epimastigotes and induced trypomastigote lysis with IC_50_/24 h of 115 *μ*g/mL. Thyme EO presented an IC_50_/24 h of 77 *μ*g/mL for epimastigotes and 38 *μ*g/mL for trypomastigotes. Thymol (**1**) (Figure 1), the primary constituent of thyme, was also tested and showed an IC_50_/24 h value of 62 *μ*g/mL for epimastigotes and an IC_50_/24 h value of 53 *μ*g/mL for trypomastigotes. These IC_50_/24 h results were similar to those obtained after treatment *in vitro* of the parasites with the reference drug benznidazole (BZ) (IC_50_/24 h approximately 50 *μ*g/mL) [53].

**Figure 2:**
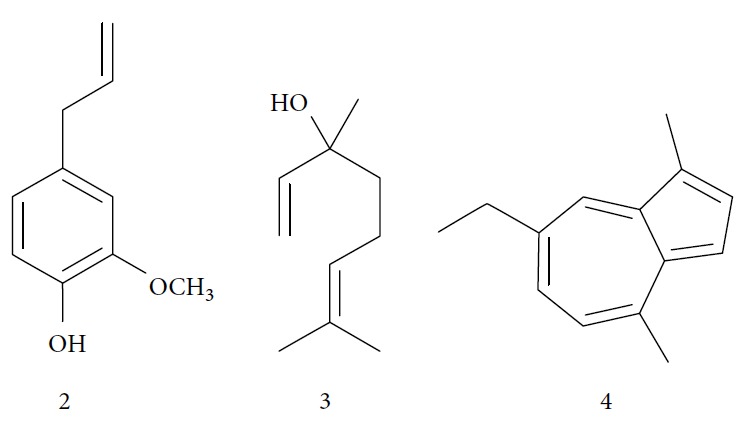
Chemical structures of eugenol (**2**), linalool (**3**), and chamazulene (**4**) - main constituents of clove (*Syzygium aromaticum* L.), basil (*Ocimum basilicum* L.), and yarrow (*Achillea millefolium* L.).

In another study, the Santoro group evaluated the EOs of three species, clove (*Syzygium aromaticum* L.), basil (*Ocimum basilicum* L.), and yarrow (*Achillea millefolium* L.) and some of their primary constituents (eugenol (**2**) and linalool (**3**)) (Figure 2). Treatment with EOs and their constituents showed that they inhibited *T. cruzi* growth, with clove EO being the most effective. Its IC_50_/24 h value was 99.5 *μ*g/mL for epimastigotes and 57.5 *μ*g/mL for trypomastigotes. Incubation of *T. cruzi* with eugenol (**2**) (the main constituent of clove) gave an IC_50_/24 h value of 246 *μ*g/mL for epimastigotes and 76 *μ*g/mL for trypomastigotes. This suggested that the chemical constituents of clove EO had a synergistic effect that increased trypanocidal activity. Linalool (**3**) (the primary constituent of basil) was also tested and gave IC_50_/24 h values of 162.5 *μ*g/mL for epimastigotes and 264 *μ*g/mL for trypomastigotes. Chamazulene (**4**) (Figure 2), one of the primary constituents of yarrow EO, was not evaluated in their study [54].

**Figure 3:**
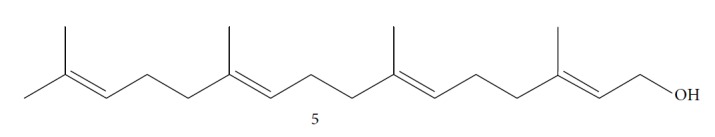
Chemical structure of geranylgeraniol, one of the main components of the ethanolic extract of *Pterodon pubescens* seeds.

Menna-Barreto and coworkers evaluated the potential trypanocidal effect of the oleaginous ethanolic extract of *Pterodon pubescens* seeds, its fractions, and geranylgeraniol, among other components isolated from the hexane fraction. The ethanolic extract was fractionated, yielding three subfractions including geranylgeraniol (**5**) (Figure 3). The EO of *P. pubescens*, its fractions, and geranylgeranyl induced dose-dependent lysis in *T. cruzi* trypomastigotes. Geranylgeraniol (**5**) was the most active compound, with an IC_50_/24 h value of 15.3 *μ*g/mL [55].

**Figure 4:**
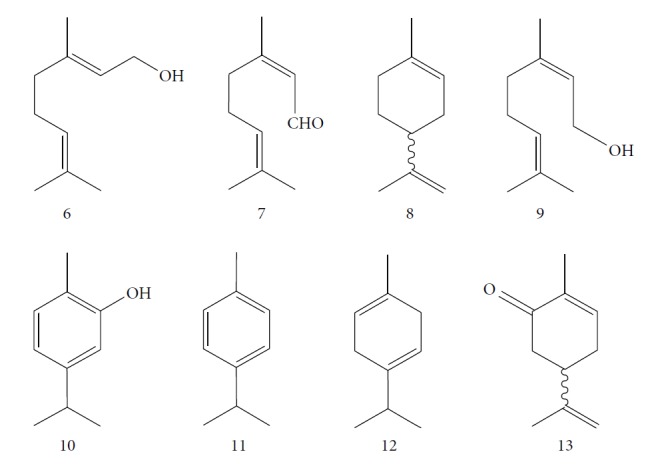
Chemical structures of carvacrol (**10**), geraniol (**6**), *p*-cymene (**11**), and other terpenes from Colombian *Lippia* spp. EO.

Escobar and coworkers evaluated the antiprotozoal activities of Colombian *Lippia* spp. EO and their major components. Nineteen EO samples were extracted from the aerial parts of five different species by microwave radiation-assisted hydrodistillation and were characterized by GC-FID and gas chromatography–mass spectrometry (GC-MS). Geraniol (**6**), neral (**7**), limonene (**8**), nerol (**9**), carvacrol (**10**), *p*-cymene (**11**), *γ*-terpenes (**12**), carvone (**13**), and thymol (**1**) were major components identified. Nine oils were active against epimastigotes from *T. cruzi* with IC_50_ values in the range of 5.5 to 32.2 *μ*g/mL. Six EOs were active against amastigotes interiorized in Vero cells with IC_50_ ranging from 12.2 to 53.0 *μ*g/mL. In addition, the authors evaluated seven of the major EO components, including thymol (**1**), carvacrol (**10**), geranial, and *p*-cymene (**11**) (Figure 4) which were active against epimastigotes, with IC_50_ values ranging from 0.3 to 28.1 *μ*g/mL. Thymol (**1**) and *S*-carvone (**13**) were the most active against intracellular amastigote-infected Vero cells, with IC_50_ values of 3.6 and 6.0 *μ*g/mL, respectively [56].

In a study by Rojas and coworkers, the authors evaluated the *in vitro* activity of EOS from 10 plants against *T. cruzi*, their cytotoxic activities, and their modulatory activities in a nitric oxide assay. The evaluated plants were *Mentha piperita L.* (mentha), *Rosmarinus officinalis L* (romero), *Chenopodium ambrosioides L.* (paico), *Eucalyptus globulus Labill* (eucalipto), *Artemisia absinthium L.* (ajenjo), *Melissa officinalis L* (toronjil), *Minthostachys setosa Brig* (muña), *Cymbopogon citratus* (hierba luisa), *Aloysia triphylla* (cedrón), and *Mentha spicata L.* (hierba buena). Of these, EOs from *Cymbopogon citratus* and *Aloysia triphylla* were the most active against epimastigote forms, inhibiting *T. cruzi* growth with IC_50_ values of 63.09 and 96.49 *μ*g/mL, respectively. There was no significant variation in the concentration of nitric oxide, and no cytotoxicity was evident [57].

Rojas and colleagues also evaluated the *in vivo* anti-*T. cruzi* effects of *Cymbopogon citratus* and *Aloysia triphylla* EOs in Balb/c mice. *C. citratus* EO administered at 250 mg/kg/day reduced parasitemia peaks from 113.92 ± 25.66 to 74.60 ± 12.37 trypomastigotes/mL. Reduction in the number of amastigotes and number of inflammatory infiltrates in the heart was also observed. When administered at 100 mg/kg/day, no reduction in the number of trypomastigotes was observed (77.40 ± 14.93 trypomastigotes/mL) [58]. *Aloysia triphylla* EO produced significant reduction in parasitemia (85.4%) with a peak of 250 mg/kg/day. They also observed a reduction in the number of amastigotes and inflammatory infiltrates in the heart [59].

**Figure 5:**
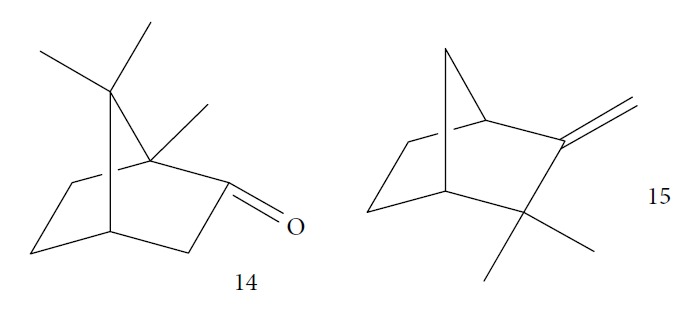
Chemical structure of camphor (**14**) and camphene (**15**), the main constituents of *Piper malacophyllum* EO.

Santos et al. evaluated *Piper malacophyllum* EO against diverse microorganisms including *T. cruzi*. Among the 28 compounds identified in the EO, (+) camphor (**14**) was the major constituent (32.8%) followed by camphene (**15**) (17.8%) (Figure 5). *P. malacophyllum* EO showed activity against epimastigote forms of *T. cruzi* with an IC_50_ value of 311.82 *μ*g/mL. This was considered a low activity value compared to that of other EOs described in the literature [60].

**Figure 6:**
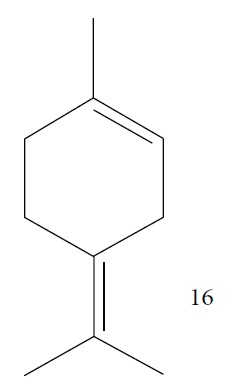
Chemical structure of terpinolene (**16**) - main constituent isolated from *Chenopodium ambrosioides* EO.

EOs from *Lippia sidoides*, *Lippia origanoides*, *Chenopodium ambrosioides*, *Ocimum gratissimum*, *Justicia pectoralis*, and *Vitex agnus-castus* were tested against *T. cruzi* by Borges and coworkers. All EOs inhibited epimastigote growth and reduced cell viability of trypomastigote forms in a dose-dependent manner. *C. ambroside* (main constituent – terpinolene (**16**) (69.9%)), *L. origanoides* (main constituent – carvacrol (**10**) (37.3%)), and *L. sidoides* (main constituent – thymol (**1**) (78.4%)) (Figures 1, 4, and 6) were the most effective against epimastigote forms (IC_50_ values of 21.3, 26.2, and 28.9 *μ*g/mL, respectively). The less active were EOs obtained from *J. pectorales*, *O. gratissimum*, and *V. agnus-castus* with IC_50_ values of 56.8, 71.1, and 157.1 *μ*g/mL, respectively. On the other hand, *L. sidoides*, *O. gratissimum* (38.4% – eugenol (**2**) as the main constituent), *C. ambrosioides*, and *L. origanoides* were found to be more effective against trypomastigotes with LC_50_/24 h values of 10.3, 11.5, 28.1, and 39.7 *μ*g/mL, respectively [61].

**Figure 7:**
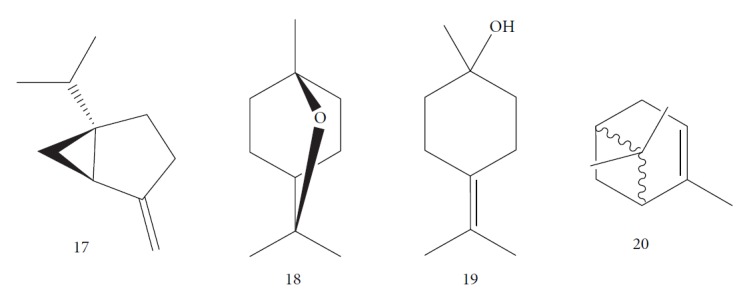
Chemical structure of the main constituent isolated from *Piper cubeba.*

Esperandim et al. investigated the activity *in vitro* of the *Piper cubeba* EO against trypomastigote and amastigote forms of *T. cruzi* and promastigote forms of *Leishmania amazonensis*. GC-MS analysis of the essential oils demonstrated than the main components were sabinene (**17**) (19.99%), eucalyptol (**18**) (11.87%), *γ*-terpineol (**19**) (6.36%), *α*-pinene (**20**) (5.82%), and camphor (**14**) (5.61%) (Figure 7). The *in vitro* activity against trypomastigotes and amastigotes of *T. cruzi* increased with increasing concentrations, giving IC_50_ values of 45.5 and 87.9 *μ*g/mL, respectively. The EO was not active against *L. amazonensis* [62].

**Figure 8:**
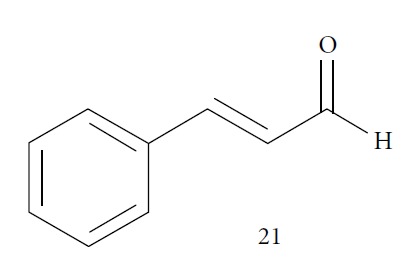
Chemical structure of (*E*)-cinnamaldehyde (**21**) - the main constituent identified in *Cinnamomum verum* EO.

In 2014, Azeredo and coworkers evaluated the *in vitro* antitrypanocidal activity of eight different EOs (*Cinnamomum verum*, *Citrus limon*, *Cymbopogon nardus*, *Corymbia citriodora*, *Eucalyptus globulus*, *Eugenia uniflora*, *Myrocarpus frondosus*, and *Rosmarinus officinalis*) against *T. cruzi*. Of these, *Cinnamomum verum* EO was the most effective against *T. cruzi* epimastigotes (IC_50_/24 h = 24.13 *μ*g/mL), followed by *Myrocarpus frondosus* (IC_50_/24 h = 60.87 *μ*g/mL) and *Eugenia uniflora* (IC_50_/24 h = 70 *μ*g/mL). No other evaluated EOs presented significant activity. In addition, *C. verum* EO showed IC_50_/24 h values of 5.05 *μ*g/mL and 20 *μ*g/mL against *T. cruzi* metacyclic trypomastigotes and intracellular amastigotes, respectively. GC-MS analysis of *C. verum* EO showed (*E*)-cinnamaldehyde (**21**) (81.52%) (Figure 8) and eugenol (**2**) (16.68%) as the main components [63].

Monzote and colleagues evaluated the *Chenopodium ambrosioides* EOs and their principal components against *Leishmania infantum*, *Plasmodium falciparum*, *Trypanosoma brucei*, and *T. cruzi*. *C. ambrosioides* EO showed activity against evaluated protozoa, with IC_50_ values ranging between 0.2 and 6.4 *μ*g/mL. Against *T. cruzi*, the IC_50_ value was 1.9 *μ*g/mL [64].

In 2015, Andrade et al. evaluated the trypanocidal activity of EOs from *Cinnamodendron dinisii* Schwacke (canellaceae) and *Siparuna guianensis* Aublet (Siparunaceae). Both EOs showed low trypanocidal activity, with IC_50_/24 h values of 209.30 *μ*g/mL for *S. guianensis* and 282.93 *μ*g/mL for *C. dinisii*. The IC_50_/24 h of the reference drug benznidazol was 12.8 *μ*g/mL [65].

**Figure 9:**
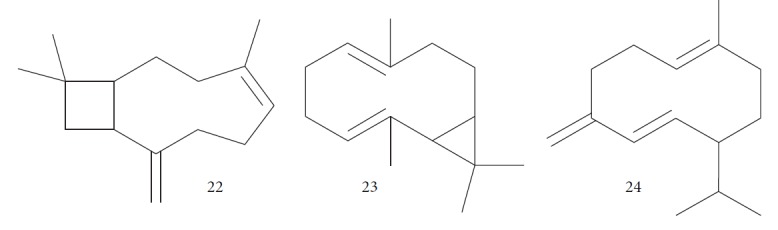
Chemical structure of (*E*)-caryophyllene (**22**), bicyclogermacrene (**23**), and germacrene (**24**) - main constituent identified in *Latana camara* EO.

The EO from *Latana camara* leaves was tested against *Leishmania braziliensis* and *T. cruzi* by Barros and coworkers. They found that *L. camara* EO was more effective against *L. braziliensis* than *T. cruzi* with IC_50_ values of 72.31 and 201.94 *μ*g/mL to promastigote and epimastigote forms, respectively. The authors also evaluated the composition of this EO by CG-MS and found that the main constituents were (*E*)-caryophyllene (**22**) (23.75%), bicyclogermacrene (**23**) (15.80%), and germacrene D (**24**) (11.73%) (Figure 9) [66, 67].

**Figure 10:**
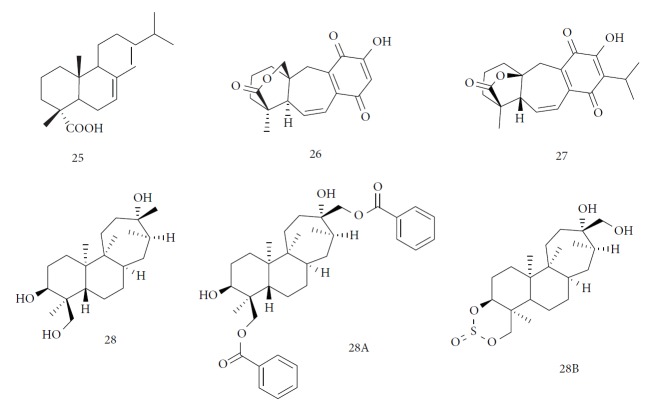
Chemical structure of abietic acid (**25**), 12-hydroxy-11,14-diketo-6,8,12-abietatrien-19,20-olide (**26**), 5-epi-icetexone (**27**), and aphidicolin (**28**) derivatives obtained by semisynthesis.

In 2016, a review published by Barros de Alencar and coworkers summarized many reports that identified the activity of diterpenes against NDs, including Chagas disease. The compounds and classes of compounds that were identified as most promising were abietic acid (**25**) derivatives, with IC_50_ values between <20 *μ*M and > 150 *μ*M (Olmo et al., 2015); 12-hydroxy-11,14-diketo-6,8,12-abietatrien-19,20-olide (**26**) isolated from *Salvia cuspidate* with IC_50_ value of 5 *μ*g/mL (Lozano *et al*., 2015); 5-epi-icetexone (**27**) (10 mg/kg/day, i.p.) used for 5 days, capable of reversing infection in Swiss mice (Lozano *et al*., 2016); and aphidicolin (**28**) derivatives obtained by semisynthesis exhibited high potency and selectivity against parasitic amastigote forms (Santos *et al.*, 2016). Two of these derivatives gave IC_50_ values of 0.6 *μ*M (**28 A**) and 0.78 *μ*M (**28 B**), with selectivity index > 100 for both (Figure 10) [68, 69].

**Figure 11:**
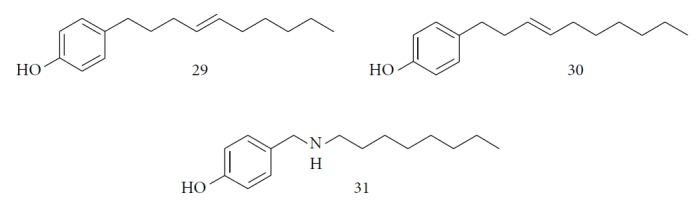
Chemical structure of alkenyphenol derivatives and its synthetic analogue (**31**).

Other semisynthetic compounds were evaluated by Varela and colleagues. The EO obtained from the areal parts (leaves) of *Piper malacophylum* (Piperaceae) presented two alkenyphenol derivatives as primary compounds: gibbilimbols A (**29**) and B (**30**), evaluated against promastigote/amastigote forms of *Leishmania infantum* and trypomastigote/amastigote forms of *T. cruzi*. Gibbilimbol B (**30**) showed better trypanocidal activity and lower toxicity to mammalian cells. These results encouraged researchers to prepare several quite simple synthetic analogues. Among these, the compound *n*-octyl-4-hydrozybenzylamine (LINS03003) (**31**) was the most potent, with IC_50_ values of 5.5 *μ*M against amastigote forms of *T. cruzi*, with a higher activity than that of the natural prototype (Figure 11) [70].

**Figure 12:**
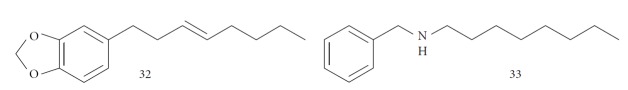
Chemical structures of 5-[(3*E*)-oct-3-en-1-il]-1,3-benzodioxole (**32**) and LINS03011 (**33**).

Spurred on by the previous results, Varela's group isolated a new natural product from the *P. malacophylum* EO — compound 5-[(3*E*)-oct-3-en-1-il]-1,3-benzodioxole (**32**) (Figure 12), and a new set of five compounds were synthesized. The isolated compound was evaluated against *L. infantum* and *T. cruzi* and presented potential against promastigote/trypomastigote/amastigote forms of *T. cruzi*, with IC_50_ values of 32–83 *μ*M and low toxicity (CC_50_ > 200 *μ*M to mammalian cells). The synthetic compounds possessed promising antiparasitic activity; however, the compounds were considered to be cytotoxic. Compound LINS03011 (**33**) (Figure 12) was the most active compound with IC_50_ values of 13.3 mM against amastigotes, comparable to the phenolic prototype LINS03003 (**31**), but with better selectivity index (SI = 24.5) and less toxicity [71].

**Figure 13:**
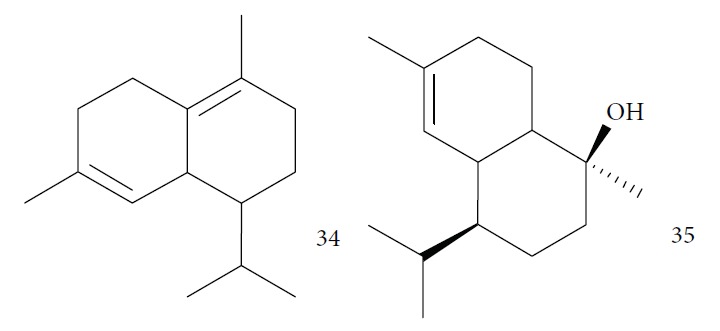
Chemical structure of the main constituents identified from *Eugenia brejoensis* EO.

Souza and coworkers evaluated the chemical composition and the *in vitro* effects of the EOs from the leaves of *Eugenia brejoensis* (*Eb*EO), *Hyptis pectinata* (*Hp*EO), *Hypenia salzmannii* (*Hs*EO), *Lippia macrophylla* (*Lm*EO), and seeds of *Syagrus coronata* (*Sc*EO) against *T. cruzi.* GC-MS allowed the identification of 114 of 162 compounds. Among these, hydrocarbon sesquiterpenes were the most abundant found in *Eb*EO (94.51%), *Hf*EO (69.84%), *Hp*EO (77.88), and *Hs*EO (56.16%). The *Lm*EO fraction had monoterpenes as its principal constituents (98.08%). The EOs presented IC_50_ values between 29 and 110.6 mg/mL for epimastigote forms, 17.39–182.49 mg/mL for trypomastigote forms, and 12.5–408.33 mg/mL for amastigote forms of *T. cruzi*. The most active was *Eb*EO, and the least active was *Sc*EO. *Eb*EO had as its main constituents *δ*-cadinene (**34**) (15.88%), *trans*-caryophyllene (**22**) (9.77%), and *α*-muurolol (**35**) (9.42%) (Figure 13) [72].

Villamizar et al. analyzed the trypanocidal effects of *Piper aduncum* EO (*Pa*EO) and its main constituents against various forms of *T. cruzi*. GC-MS identified nerolidol and linalool (**3**) as the major *Pa*EO components. *Pa*EO showed IC_50_/24 h = 12.1 *μ*g/mL against metacyclic trypomastigotes and IC_50_/24 h values of 9 *μ*g/mL against amastigotes forms. Among the two main constituents isolated and identified, linalool (**3**) (Figure 2) was the best, showing trypanocidal activity with an IC_50_/24 h value of 306 ng/mL [73].

**Figure 14:**
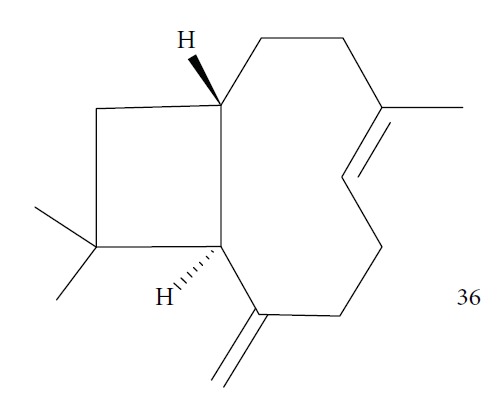
Chemical structure of *γ*-caryophyllene (**36**) - one of the main constituents identified from *Syzygium aromaticum* EO.

A recent study by Junior and colleagues evaluated the efficacy of the EO of *Syzygium aromaticum* (*Sa*EO) administrated orally, alone, and in combination with benznidazole (BZ) in mice infected with *T. cruzi* IV strain. GC-MS analysis identified eugenol (**2**) (82.2%) and *γ*-caryophyllene (**36**) (13.0%) (Figure 14) as the main components of *Sa*EO. Treatment with *Sa*EO promoted reduction in 1/5 parameters derived from the parasitemia curve when compared with untreated control groups. The groups treated with BZ and BZ + *Sa*EO had reductions in 4/5 of these parameters, with similar profiles of the parasitemia curve. In addition, the animals treated with BZ and BZ + *Sa*EO presented lower patency periods compared with animals treated only with *Sa*EO, as well as lower positivity of blood cultures compared with the untreated control group. With respect to cure rates, *Sa*EO showed 12.5% (1/8), BZ showed 25.0% (1/4), and BZ + *Sa*EO showed 37.5% (3/8), considered a very interesting result [43].

### 1.2. Essential Oils and Components with Anti-*Trypanosoma brucei* Activity

*Trypanosoma brucei* (Trypanosomatid family) is the principal protozoan responsible for causing the type of trypanosomiasis known as sleeping sickness (or human African trypanosomiasis). It is classified as a neglected disease and is transmitted by tsetse flies (*Glossina* spp., Diptera: Glossinidae).

In humans, this infection is caused by two subspecies of *T. brucei* (*T. brucei gambiense*, predominant in the West Africa, and *T. brucei rhodesiense*, predominant in the East Africa). There is also *T. brucei brucei* that is infective only in animals. It is endemic to Sub-Saharan Africa. Despite the fact that it has been declining over the last two decades, *T. brucei* nevertheless affects approximately 30,000 people annually (as well as about 60 million people - distributed in 36 countries - that inhabit areas at risk), with high mortality rates, mainly in rural populations that have little access to necessary treatments, causing substantial socioeconomic impact [74–86].

Human African trypanosomiasis, after parasite inoculations in the human host (or animal, *mutatis mutandis*), evolves in two stages. The primary stage is a hemolymphatic phase, or the parasitic cell replication stage, followed by a secondary meningoencephalitic phase, in which the parasites cross the blood-brain barrier and invade the central nervous system [86, 87]. This stage of the disease is characterized by a symptomatology including fever, headache, swelling of the lymph nodes, skin rashes, splenomegaly, confusion, sensory disturbances, motor coordination deficiency, and sleep disturbances (characteristic of the disease). It can be fatal if not properly treated [88].

The primary treatment is chemotherapy [89–91]; however, only four drugs are approved for human use: suramin, pentamidine, melarsoprol, and eflornithine [92]. The former two alternatives are used in the first stage of the disease, while the later two are used only in the last stage, representing alternatives of increased toxicity of the treatment [46, 88–92]. However, as was observed for the treatment of the other NDs, the treatment of *T. brucei* includes only a few effective drugs, and those that exist carry substantial toxicities, high costs, absence of sustainable production, and development of resistance by the protozoa [93–97]. There is an urgent need for the discovery and development of new drugs that are more efficient and secure. This need is reflected in the growing interest of many research groups in the search for new compounds with anti-*T. brucei* potential [98–106].

In many cases, traditional medicine has been the only alternative available for treatments based on natural plant products, and the treatment of *T. brucei* infections is no exception [64, 104, 107–114]. Natural plant products are excellent sources of safe and effective compounds to combat human African trypanosomiasis [88, 107, 115]. These natural bioactive products are for the most part plant secondary metabolites, including several classes of promising bioactive compounds, among which are EOs [92, 116–118].

As a reflection of the increasing interest in EOs for use against *T. brucei* evaluation, a number of investigators have studied their potential, from determination of vegetal sources in various plant parts (aerial or radicular systems) to acquisition of species and types of essential oils.

In parallel with the increase in the use of EOs obtained from various parts of plants (aerial parts, fruits, flowers, and/or root systems) to combat sleeping sickness, several investigators worldwide have expanded their studies into the potential of these EOs and their main constituent chemicals against *T. brucei*.

**Figure 15:**
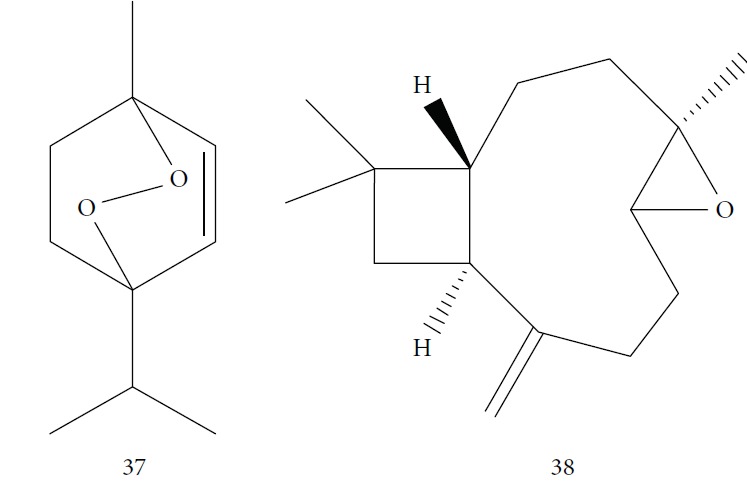
Chemical structures of ascaridole (**37**) and caryophyllene oxide (**38**) – one of the main components isolated from *C. ambrosioides* EO.

One of these studies (Monzote and colleagues [64]) evaluated the EO from *Chenopodium ambrosioides L.* (*Ca*EO), an aromatic plant with known biological potential from the Americas and Africa [119]. The plant's anti-*Leishmania* sp. activities have already been identified [120]. The main *Ca*EO components carvacrol (**10**) (62%), ascaridole (**37**) (22%), and caryophyllene oxide (**38**) (5%) (Figure 15) demonstrated substantial growth inhibition of *T. brucei* parasites (IC_50_ = 0.2 ± 0.07 *μ*g/mL), with activity comparable to that of the standard drug suramin (IC_50_ of 0.05 ± 0.05 *μ*g/mL). These results, demonstrating the broad antiprotozoal spectra of *Ca*EO, are an example of the potential of their use in endemic areas where more than one infectious species is present.

**Figure 16:**
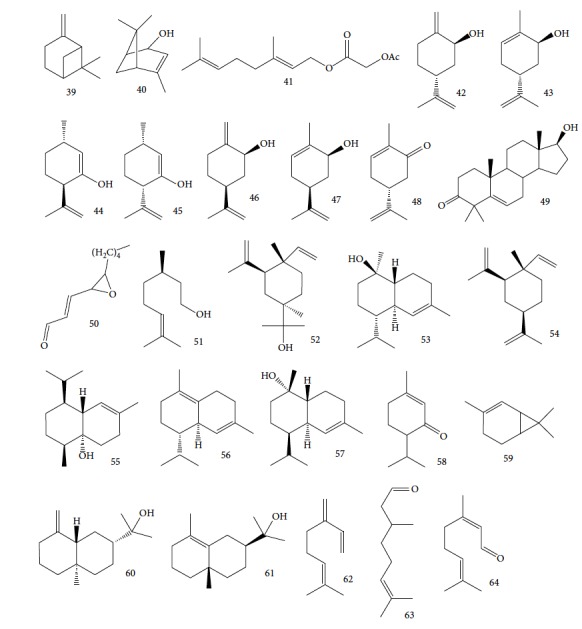
Chemical structures of several terpenes isolated from *Cymbopogon* species EO.

EOs from *Cymbopogon* species have aroused interest of many research groups because of their antitrypanosomal activities, as the example of their growth inhibitory activity against *T. brucei* [121, 122]. Kpoviessi et al. [122] evaluated the EOs of fresh leaves from four *Cymbopogon* species consisting primarily of monoterpenes and sesquiterpenes (*C. citrates* – with 29 compounds, *C. giganteus* – with 53 compounds, *C. nardus –* with 28 compounds, and *C. schoenantus –* with 41 compounds). The principal identified constituents were as follows: from *C. citrates*: geranial (**6**), neral (**7**), *β*-pinene (**39**), *cis*-geraniol (or nerol) (**9**), *cis*-verbenol (**40**), and geranyl acetate (**41**); from *C. giganteus*: *trans*-*p*-mentha-1(7),8-dien-2-ol (**42**), *trans*-carveol (**43**), *trans*-*p*-mentha-2,8-dienol (**44**), *cis*-*p*-mentha-2,8-dienol (**45**), *cis*-*p*-mentha-1(7),8-dien-2-ol (**46**), limonene (**8**), *cis*-carveol (**47**), *cis*-carvone (**48**), 4,4-dimethylandrost-5-en-3-one (**49**) and *trans*-carene-4,5-epoxy (**50**); from *C. nardus*: *β*-citronellol (**51**), elemol (**52**), limonene (**8**), *α*-cadinol (**53**), *β*-elemene (**54**), cubenol (**55**), germacrene-D (**24**), geranyl acetate (**41**), *δ*-cadinene (**34**), and *δ*-cadinol (**56**); and from *C. schoenantus*: piperitone (**57**), (+)-2-carene (**58**), limonene (**8**), elemol (**52**), *β*-eudesmol (**59**), *trans*-*p*-mentha-2,8-dienol (**44**), *cis*-*p*-mentha-2,8-dienol (**45**), and *τ*-eudesmol (**60**) (Figure 16).

These authors measured the cytotoxicity against WI38 and CHO cells and *in vitro* activity against *T. brucei*. All EOs evaluated were active, with IC_50_ values of 5.71 ± 1.40 *μ*g/mL for *C. nardus*, 2.10 ± 0.89 *μ*g/mL for *C. schoenantus*, 1.83 ± 0.13 *μ*g/mL for *C. citrates*, and 0.25 ± 0.11 *μ*g/mL for *C. giganteus*. The reference drug suramin had an IC_50_ of 0.11 ± 0.02 *μ*g/mL. *C. giganteus* EO was the most active oil and demonstrated the largest selectivity index (SI of >200, as well as SI of 21.73 for *C. citrates*, 8.75 for *C. nardus*, and 23.81 for *C. schoenantus*). The anti-*T. brucei* evaluation of some of the components from these EOs demonstrated high potentialities: *β*-myrcene (**62**) (IC_50_ = 2.24 *μ*g/mL), citronellal (**63**) (IC_50_ = 2.76 *μ*g/mL), *R*(+)-limonene (**8**) (IC_50_ = 4.24 *μ*g/mL), citral (**64**) (IC_50_ = 5.98 *μ*g/mL), and *β*-citronellol (**51**) (IC_50_ = 6.45 *μ*g/mL) (Figure 16). Of these, limonene (**8**) was the principal component of three of the four EOs, including *C. giganteus* (the most active), probably tightly influencing their antitrypanosomal activities.

The composition of essential oils of *C. giganteus* was highly rich in oxygenated monoterpenes that should greatly contribute their potential against *T. brucei*. The results showed that these compounds should be analyzed separately with greater depth in order to identify new alternatives for the treatment of human African trypanosomiasis.

**Figure 17:**
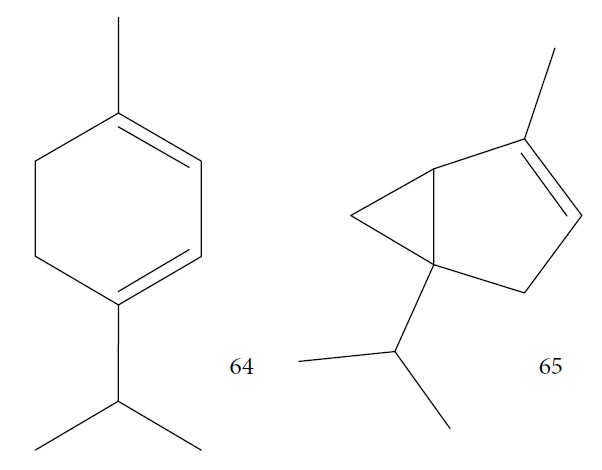
Chemical structures of *γ*-terpinene (**64**) and *α*-thujene (**65**) isolated from *Ocimum gratissimum*.

Kpoviessi et al. [123] analyzed the EOs from the aerial parts (seeds, leaves, and stems) of *Ocimum gratissimum* in various vegetative stages. Their analysis of the principal EO components from *O. gratissimum* revealed 47 constituents (the majority of them hydrocarbons and a minority of oxygenated compounds). Of these, *p*-cymene (**11**), thymol (**1**), *γ*-terpinene (**64**), *β*-myrcene (**61**), and *α*-thujene (**65**) (Figure 17) were the most abundant. *O. gratissimum* EO demonstrated moderate anti-*T. brucei* activity (IC_50_ of 27.23 ± 3.74 *μ*g/mL), as also previously observed with many of their principal compounds [94] as observed with thymol (**1**) (IC_50_ of 22.86 *μ*g/mL) and *p*-cymene (**11**) (IC_50_ of 76.32 *μ*g/mL). Thus, the primary isolated components did not possess strong anti-*T. brucei* activity and did not act synergistically (demonstrating an interesting selectivity index of 410 for the leaves and seeds portions). Conversely, the ethanolic extract of *O. gratissimum* demonstrated IC_50_ values less than 15 *μ*g/mL, leading us to believe that other bioactive compounds that were not present in the EO were responsible for the anti-*T. brucei* activity, justifying its continued use in traditional medicine.

**Figure 18:**
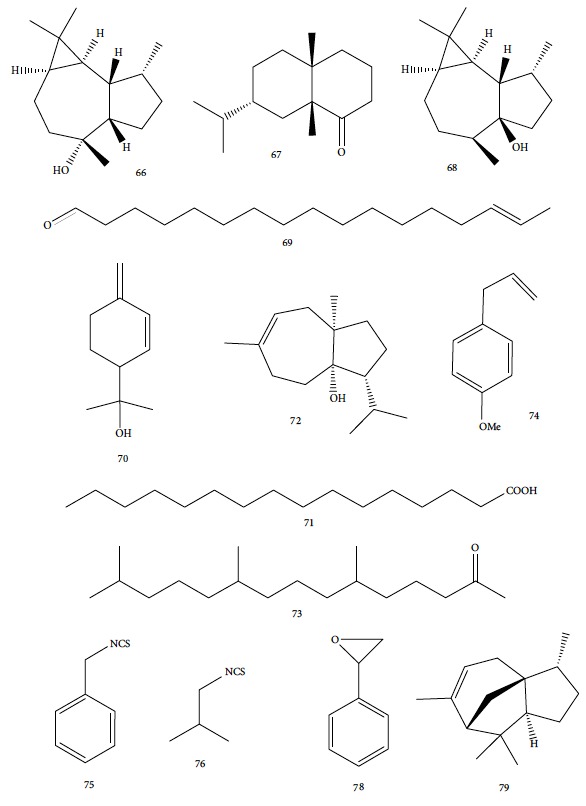
Chemical structures of constituents isolated from plants used in traditional Ethiopian medicine.

Nibret and Wink [94] evaluated anti-*T. brucei* activity and cytotoxicity against human leukemic cells HL-60 in several plants with biological potentialities well-known in traditional Ethiopian medicine, including *Hagenia abyssinica* (female flower), *Leonotis ocymifolia* (aerial parts), and *Moringa stenopetala* (seeds). The results were compared to those of the standard drug diminazene aceturate (IC_50_ = 0.088 *μ*g/mL and SI > 1464). CG-MS revealed 20 components of *H. abyssinica* (including ledol (**66**), valeranone (**67**), palustrol (**68**), *E*-15-heptadecenal (**69**), *α*-phellandren-8-ol (**70**), and verbenol (**40**) as primary compounds) and 68 components for *L. ocymifolia* (including caryophyllene oxide (**38**), palmitic acid (**71**), carotol (**72**), camphor (**14**), hexahydrofarnesyl acetone (**73**), estragole (**74**), and linalool (**3**) as the primary compounds) and for *M. stenopetala*, including benzyl isothiocyanate (**75**) and isobutyl isothiocyanate (**76**) as primary compounds. *M. stenopetala* EO demonstrated greater antitrypanosomal activity (IC_50_ of 5.03 *μ*g/mL), but with a lower selectivity index (SI = 2.31). Among the isolated compounds, the best results were observed with benzyl isothiocyanate (**75**) (IC_50_ = 1.20 *μ*g/mL and SI = 3.85), cinnamaldehyde (**21**) (IC_50_ = 2.93 *μ*g/mL and SI = 11.13), styrene oxide (**78**) (IC_50_ = 3.76 *μ*g/mL and SI = 19.92), and (−)-*α*-cedrene (**79**) (IC_50_ = 4.07 *μ*g/mL and SI = 5.45). Cinnamaldehyde (**21**) and styrene oxide (**78**) (Figure 18) were the compounds with the best combined results (IC_50_ and SI). Nevertheless, they observed that benzyl isothiocyanate (**75**) was the majority component of *M. stenopetala* EO and was therefore responsible for the anti-*T. brucei* potentiality of this plant. Despite a low selectivity index, the compound can be utilized as a basis for developing new antitrypanosomial drugs.

**Figure 19:**
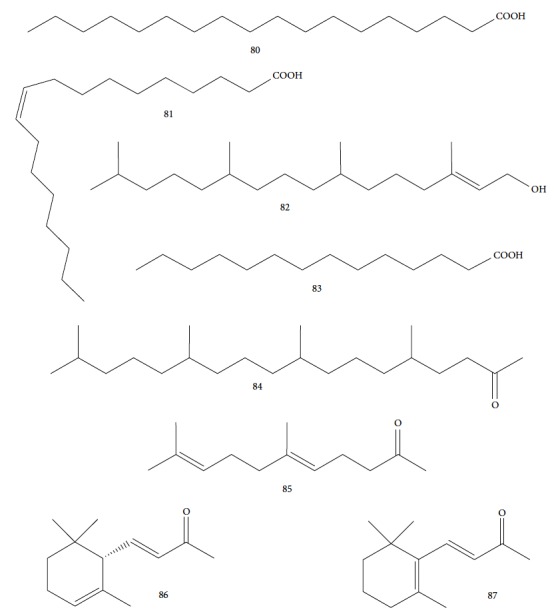
Chemical structures of some compounds isolated from *Keetia leucantha*.

*Keetia leucantha*, a plant used in traditional medicine of West African countries, was the object of study of Bero and colleagues [124]. They investigated the chemical composition of *Kl*OE and their anti-*T. brucei* activities. The plant is used empirically in the treatment of sleeping sickness [102]. *K. leucantha* EO demonstrated IC_50_ values of 20.9 ± 12.6 *μ*g/mL for bloodstream forms of *T. brucei brucei* (compared to IC_50_ = 0.11 ± 0.02 *μ*g/mL for the standard drug suramin). Analysis of the secondary metabolites contained in the OE returned 42 components, including *n*-hexadecanoic acid (43.46%) (**80**), oleic acid (9.51%) (**81**), phytol (5.68%) (**82**), tetradecanoic acid (**83**) (5.19%), and phytone (**84**) (4.3%) (Figure 19). Some of these compounds were also evaluated and presented moderate to weak activities, confirming the moderate activity of *Kl*OE against *T. brucei*. The IC_50_ values of the isolated EO components were as follows: phytol (**82**) (IC_50_ = 19.1 ± 2.3 *μ*g/mL), oleic acid (**81**) (IC_50_ = 64.3 ± 0.5 *μ*g/mL), and *n*-hexadecanoic acid (**80**) (IC_50_ > 100 *μ*g/mL). The best values were observed with the minority components: geranylacetone (**85**) (IC_50_ = 16.2 ± 12.5 *μ*g/mL), *α*-ionone (**86**) (IC_50_ = 13.1 ± 5.9 *μ*g/mL), and *β*-ionone (**87**) (IC_50_ = 10.5 ± 5.8 *μ*g/mL) (Figure 19). These components were evaluated against procyclic forms of *T. brucei*, and they were found to be even less active. According to the authors, these results can be an indication of the mechanisms of action of inhibition of *T. brucei brucei* growth. The authors suggest inhibition of glycolysis as a possible mechanism of action, as this is the main pathway of ATP synthesis for the bloodstream forms that do not possess the Krebs cycle or mitochondrial respiratory chain coupled to ATP. This phenomenon may explain their greater sensitivities compared with procyclic forms, highlighting the inhibitory activity of *β*-ionone (**87**) against glyceraldehyde-3-phosphate dehydrogenase (32.6% ± 3.4% at 20 *μ*g/mL); however, the inhibitory effect was not significant for *α*-ionone (**86**) (0.00% ± 7.2% at 20 *μ*g/mL) or phytol (**82**) (6.8% ± 0.7% at 20 *μ*g/mL).

**Figure 20:**
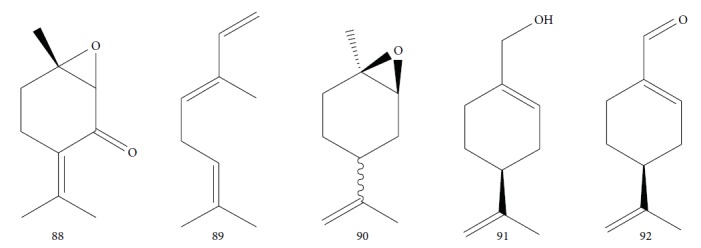
Chemical structures of rotundifolone (**88**), (−)-perillyl aldehyde (**92**), and other compounds isolated from *Mentha crispa* EO.

Another well-known plant in traditional medicine, *Mentha crispa*, was evaluated for EO activity by Sousa et al. [125]. They studied four activities against *T. brucei* bloodstream forms, as well as their cytotoxicity against human leukemic cells (HL-60). Phytochemical analysis identified major EO components, including rotundifolone (**88**) (58.11%), limonene (**8**) (10.58%), myrcene (**61**) (7.79%), germacrene (**24**) (6.55%), cis-*β*-ocimene (**89**) (5.01%), and *β*-pinene (**39**) (4.43%), as well as four other monoterpenic analogues: (+)-limonene epoxide (**90**), (−)-limonene epoxide (**90**), (−)-perillyl alcohol (**91**), and (−)-perillyl aldehyde (**92**) (Figure 20). The isolated compounds and the EO demonstrated dose-dependent activity in their minimum inhibitory concentration (MIC) values, varying from 1 to 100 *μ*/mL, and their GI_50_, varying from 0.3 to 13.3 *μ*g/mL, compared to the standard drug suramin (MIC = 0.1 *μ*g/mL, GI_50_ = 0.05 ± 0.003 *μ*g/mL, SI ≥ 2000). Greater potentialities were observed for *Mc*EO (MIC = 1 *μ*g/mL, GI_50_ = 0.33 ± 0.03 *μ*g/mL, SI = 25), rotundifolone (**88**) (MIC = 1 *μ*g/mL, GI_50_ = 0.32 ± 0.05 *μ*g/mL, SI = 10.6), and (−)-perillyl aldehyde (**92**) (Figure 20) (MIC = 1 *μ*g/mL, GI_50_ = 0.31 ± 0.02 *μ*g/mL, SI = 45). These compounds were only slightly less active than the reference drug. The presence of rotundifolone (**88**) as the major component in the *Mc*EO should be the primary compound responsible for its high anti-*T. brucei* activity.

Structurally comparing these major components with other analogues, the presence of an *α*,*β*-unsaturated carbonyl appeared to favor anti-*T. brucei* activity, because when these components were absent there was significant loss of activity. The change in the position of this *α*,*β*-unsaturated carbonyl, and the change of position of an additional unsaturation outside of the ring, appeared to strongly influence the cytotoxicity in HL-60, where (−)-perillyl aldehyde (**92**) showed less toxicity than did rotundifolone (**88**), but with a better selectivity index [125].

**Figure 21:**
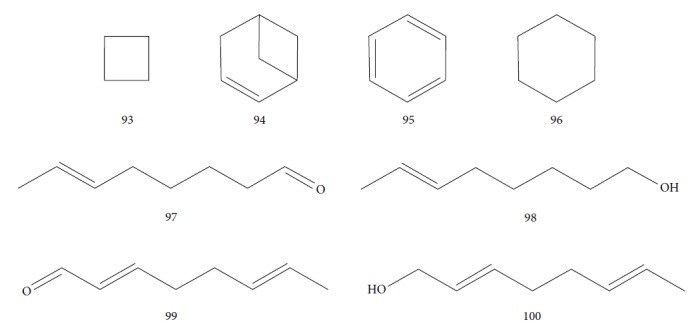
Chemical structures of main constituents isolated from *Cymbopogon citratus* – leaves, *Eucalyptus citriodora* – leaves, *Eucalyptus camaldulensis* – leaves, and *Citrus sinensis* – fruit peels.

Habila et al. [115] investigated the potential activity against *T. brucei brucei* of EOs from four plants (*Cymbopogon citratus* – leaves, *Eucalyptus citriodora* – leaves, *Eucalyptus camaldulensis* – leaves, and *Citrus sinensis* – fruit peels). CG-MS of EOs demonstrated an abundance of cyclobutane (**93**) (96.08%) in *C. sinensis*; eucalyptol (**18**) (75.04%), bicyclo[3.1.1]hept-2-ene (**94**) (10.27%), benzene (**95**) (6.23%), and cyclohexene (**96**) (4.92%) in *E. camaldulensis*; 6-octenal (**97**) (77.11%) and 6-octen-1-ol (**98**) (14.09%) in *E. citriodora*; and citral (**63**) (38.32%) and 2,6-octadienal (**99**) (35.05%) and 2,6-octadien-1-ol (**100**) (26.63%) in *C. citratus* (Figure 21).

The death of the parasites was observed after the use of 0.4 g/mL of the EOs. The EO most capable of killing parasites quickly was the *C. citratus* EO (3 min). EOs from *E. citriodora* and *E. camaldulensis* killed parasites in 4 minutes, and *C. sinensis* EO killed parasites in 5 min. In smaller doses, the time of death increased to 6 min (0.2 g/mL) and 15 min (0.1 g/mL) for *C. sinensis*, 8 min (0.2 g/mL) and 17 min (0.1 g/mL) for *E. camaldulensis*, 8 min (0.2 g/mL) and 22 min (0.1 g/mL) for *E. citriodora*, and 4 min (0.2 g/mL) and 11 min (0.1 g/mL) for *C. citratus*. Thus, their activities appeared to be dose-dependent and the four EOs demonstrated powerful activity, causing the death of protozoa in short periods of time, compared with that of the reference drug (diminaveto) that promoted the death of the parasites in equivalent times.

**Figure 22:**
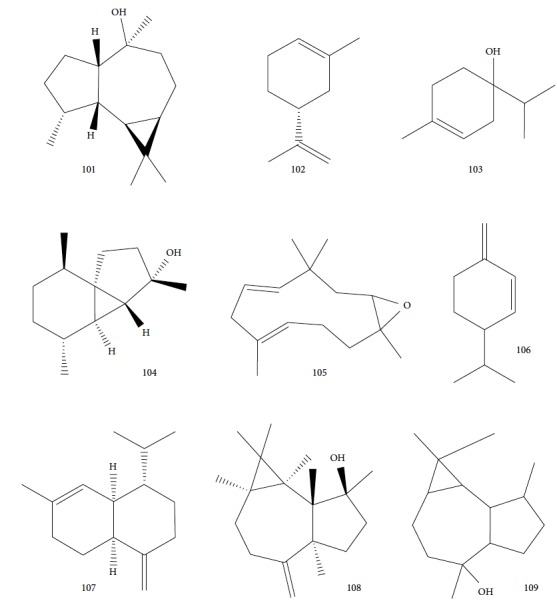
Chemical structures of isolated terpenoids from *Piper* genus.

Gutiérrez et al. [126] evaluated a species of the *Piper* genus, a well-known source of bioactive compounds [127]. *Piper ossanum* (leaves) was collected in two locations in Cuba (Bauta and Ceiba). Chromatograms of the EOs demonstrated the presence of 43 components for the Bauta species (*Po*B) and 39 for the Ceiba species (*Po*C). In both, the primary components were piperitone (**57**), camphene (**15**), camphor (**14**), and viridiflorol (**101**) (Figure 22), with P*o*B being more abundant (20.07% for *Po*B, and 19.01% for *Po*C). Some quantitative differences were observed in *Po*B and *Po*C EO constituents: camphor (**14**) (13.87% for *Po*B and 9.41% for *Po*C), viridiflorol (**101**) (12.97% for *Po*B and 18.80% for *Po*C), and camphene (**15**) (7.41% for *Po*B and 5.39% for *Po*C). *Po*B has as minor components *p*-cymene (**11**), sylvestrene (**102**), (*Z*)- and (*E*)-*β*-ocimene (**89**), terpinen-4-ol (**103**), cubebol (**104**), and humulene epoxide (**105**); *Po*C possessed small quantities of *β*-phellandrene (**106**), *γ*-muurolene (**107**), spathulenol (**108**), and globulol (**109**) (Figure 22). The values of the sensitivity of *T. brucei* against the EOs showed no significant difference in terms of IC_50_ values (8.1 *μ*g/mL for *Po*B and 8.4 *μ*g/mL for *Po*C), suggesting that the various compositions of their constituents were not decisive for a differentiation of their potential, classified as weak when compared to the standard drug suramin (IC_50_ = 0.05 *μ*g/mL). Both EOs demonstrated low toxicity in a human fetal lung fibroblast cell line (MRC-5) (4.2 *μ*g/mL for *Po*B and 0.95 *μ*/mL for *Po*C). Evaluation of the *T. brucei* activity of the main compounds of each EO is necessary in order to identify more promising compounds.

**Figure 23:**
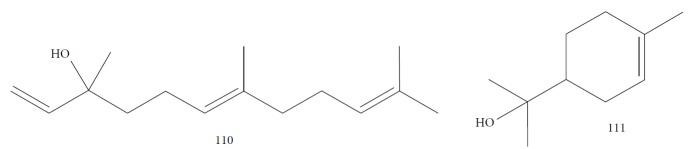
Chemical structure of (*E*)-nerolidol (**110**) and *α*-terpineol (**111**) – active compounds with anti-*T. brucei brucei* activity, isolated from *Strychnos spinosa* EO.

*Strychnos spinosa* leaves are widely utilized in African trypanosomiasis treatment [128]. Hoet et al. [129] studied the cytotoxicity and the anti-*T. brucei brucei* activity of *Ss*EO leaves and 16 of their components. *Ss*EO leaves contained more than 100 constituents, including palmitic acid (**71**) (34.3%), linalool (**3**) (16%), (*E*)-phytol (**82**) (6.7%), and (*E*)-geraniol (**6**) (4%). *Ss*EO showed moderately activity (IC_50_ = 13.5 *μ*g/mL) and weak selectivity index (SI = 4.4). Of the pure components tested, the prominent compounds were (*E*)-nerolidol (**110**) (Figure 23) (IC_50_ = 1.7 ± 0.05 *μ*g/mL, SI = 35.7), linalool (**3**) (IC_50_ = 2.5 ± 0.3 *μ*g/mL, SI ≥ 40), (*E*)-geranylacetone (**85**) (IC_50_ = 5.2 ± 0.6 *μ*g/mL, SI ≥ 19.2), *β*-ionone (**87**) (IC_50_ = 5.5 ± 0.2 *μ*g/mL, SI = 11.7), (*E*)-phytol (**82**) (IC_50_ = 6.2 ± 0.2 *μ*g/mL, SI = 3.4), and *α*-terpineol (**111**) (Figure 23) (IC_50_ = 7.0 ± 3.3 *μ*g/mL, SI ≥ 14.3). Among these, only linalool (**3**) and (*E*)-phytol (**82**) were found in abundance in *Ss*EO, leading to the conclusion that interaction with other compounds decreased the activity of the EO. In addition, the presence in minimal quantities of the more active compounds (identified as minority components) prevented the EOs from exerting their potential effects. Palmitic acid (**71**), the primary component for this species, was inactive and highly toxic (SI = 0.6).

Preliminary results [101] demonstrated good antitrypanosomal activity of dichloromethane extracts of *S. spinosa* (IC_50_ = 1.5 *μ*g/mL), causing us to speculate that there are active components that are not EO constituents that should be evaluated more intensively. The better results observed with linalool (**3**) and (*E*)-nerolidol (**110**) reflect the need for further studies of these substances and for investigation of the potential of other oxygenated terpenes to identify alternatives for human African trypanosomiasis treatment.

**Figure 24:**
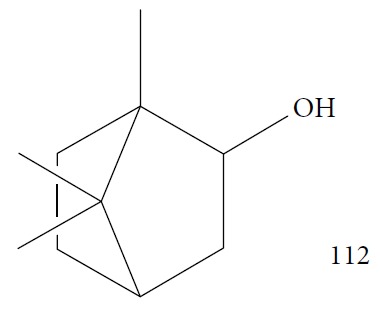
Chemical structure of borneol (**112**) – one of the compounds identified in the *Kadsura longipedunculata* EO.

Mulyaningsih et al. [130] investigated the *Kadsura longipedunculata* EO that had 50 identified components. The EO fraction was composed of 75% sesquiterpenes and 22.63% monoterpenes, including *δ*-cadinene (**34**) (21.79%), camphene (**15**) (7.27%), borneol (**112**) (6.05%) (Figure 24), cubenol (**55**) (5.12%), and *δ*-cadinol (**56**) (5.11%). *Kl*EO and camphene (**15**) were evaluated against bloodstream forms of *T. brucei brucei* and human hepatocellular liver carcinoma (HepG2); however, the results were not encouraging. *Kl*EO had an IC_50_ of 50.52 ± 0.029 *μ*g/mL and an IC_50_ of 136.96 *μ*g/mL. The IC_50_ of the pure compounds were 80.66 ± 0.87 *μ*g/mL for camphene (**15**) and 70.00 ± 1.28 *μ*g/mL for borneol (**112**), both unfavorable cytotoxicity profiles. These results indicate that the active substances of *K. longepedunculata* useful against human African trypanosomiasis treatment were not part of their EO constituents.

Hamdan and coworkers [131] evaluated the EOs of two Egyptian species of *Citrus* sp., *C. jambhiri* and *C. pyriformis*. For both species, 94 compounds were identified, with limonene (**8**) the major compound (92.48% for *C. jambhiri* and 75.56% for *C. pyriformis*). Both EOs had poor activity against bloodstream forms of *T. b. brucei*, with IC_50_ values of 72.47% ± 0.87 *μ*g/mL for *C. jambhiri* and of 71.29 ± 0.38 *μ*g/mL for *C. pyriformis*, much less active than the standard drug diminazene aceturate (IC_50_ = 0.0832 ± 0.0003 *μ*g/mL).

**Figure 25:**
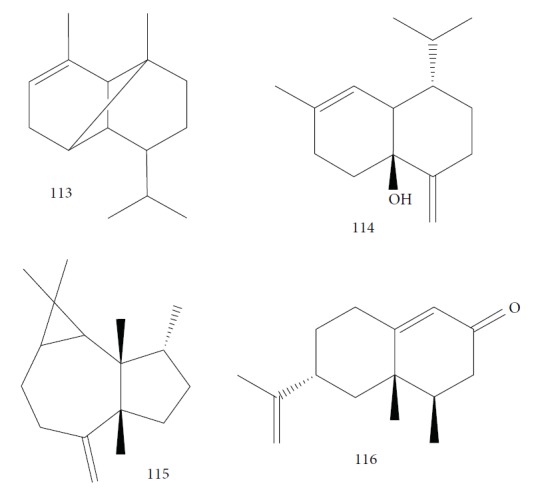
Chemical structure of compounds isolated from *Eugenia uniflora* EO.

Cruz et al. [119] evaluated the *Eugenia uniflora* EO from leaves and identified 87 compounds (spathulenol (**108**) – 15.8%, *α*-copaene (**113**) – 10.96%, muurola-4,10-dien-1*β*-ol (**114**) – 9.3%, caryophyllene oxide (**38**) – 8.93%, *allo*-aromadendrene (**115**) – 5.5%, and nootkatone (**116**) – 5.17%) (Figure 25). The oxygenated sesquiterpenes (54.09%) were the most abundant class of compounds. An *in vitro T. b. brucei* evaluation demonstrated moderate activity of *Eu*EO (IC_50_ = 11.20 ± 2.17 *μ*g/mL) with a weak selectivity index (SI = 6.82). The reference drug suramin had an IC_50_ of 0.16 ± 0.03 *μ*g/mL.

**Figure 26:**
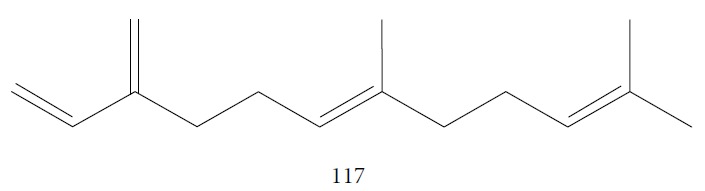
Chemical structure of (*E*)-*β*-farnesene (**117**) – one constituent from *Erigeron floribundus* EO.

Petrelli et al. [132] investigated the EO of *Erigeron floribundus* aerial parts, containing 85 constituents. The primary class of constituents was sesquiterpenes (60.4% - being 38.5% oxygenated and 21.9% hydrocarbons). The main components were caryophyllene oxide (**38**) (12.4%), spathulenol (**108**) (12.2%), (*E*)-*β*-farnesene (**117**) (Figure 26) (5.5%), and (*E*)-caryophyllene (**22**) (4.2%), in addition to limonene (**8**) (8.8%). *Ef*EO was evaluated for its antitrypanosomal activity against *T. brucei* and cytotoxicity in a human breast adenocarcinoma cell line (A375). There was moderate activity (IC_50_ = 33.5 ± 2.7 *μ*g/mL) with a low selectivity index value (SI ≥ 5.97). Their main constituent, caryophyllene oxide (**38**), was also assayed and showed up inactive, reflecting the low antitrypanosomal activity of the EO. However, the evaluation of another potential major component, limonene (**8**), demonstrated promising activity (IC_50_ = 5.6 ± 1.6 *μ*g/mL and SI ≥ 17.85), being the main contributor to the expression of EO potentiality. The authors concluded that this difference of activity in favor of limonene (**8**) could be attributed to the degree of its unsaturation, in addition to its exocyclic methylene group. The occurrence of a possible binding site with SH groups of proteins may be an indicator of their mechanism of action. However, this speculation requires more detailed investigation.

EOs from aerial parts of a variety of plant species were analyzed by Costa et al. [88], including *Cymbopogon citratus L.* (leaves), *Distichoselinum tenuifolium* (aerial parts), *Juniperus oxycedrus* (leaves and berries), *Lavandula luisieri* (influorescences), *Lavandula viridis* (aerial parts), *Lippia graveolens* (aerial parts), *Mentha cervina* (leaves), *Mentha x piperita* (leaves), *Origanum virens* (leaves), *Rosmarinus officinalis* (leaves), *Seseli tortuosum* (aerial parts), *Syzygium aromaticum* (floral buttons), *Thymbra capitata* (aerial parts), *Thymus capitellatus* (aerial parts), *Thymus mastichina* (leaves), and *Thymus zygis* sylvestris (leaves).

**Figure 27:**
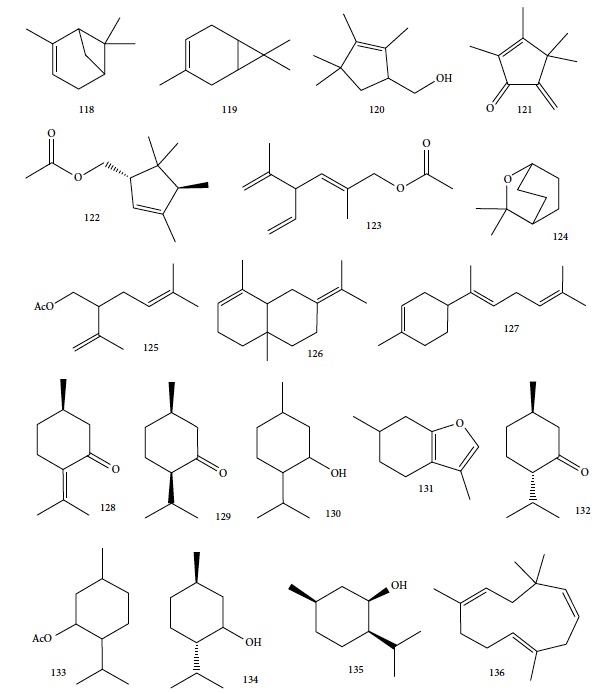
Chemical structure of main components identified in the study of Costa et al. [88].

CG-MS allowed identification of the main components, including geranial (**6**) (45.7%), neral (**7**) (32.5%), and *β*-myrcene (**61**) (11.5%) for *C. citratus*; *β*-myrcene (**61**) (84.6%) and limonene (**8**) (2.2%) for *D. tenuifolium*; *α*-pinene (**118**) (54.7%), germacrene D (**24**) (10.4%), and *β*-myrcene (**61**) (17.8%) for the berries of *J. oxycedrus*; *α*-pinene (**118**) (65.5%), Δ-3-carene (**119**) (5.7%), *β*-phellandrene (**106**) (3.2%), and *β*-myrcene (**61**) (2.7%) for leaves of *J. oxycedrus*; 1,1,2,3-tetramethyl-4-hidroxymethyl-2-cyclopentene (**120**) (2.4%), 2,3,4,4-tetramethyl-5-methylene-cyclopent-2-enone (**121**) (5.2%), trans-*α*-necrodyl acetate (**122**) (16.0%), lyratyl acetate (**123**) (3.5%), 1,8-cineole (**124**) (18.9%), lavandulyl acetate (**125**) (7.2%), linalool (**3**) (3.1%), and *α*-pinene (**118**) (2.3%) for *L. luisieri*; 1,8-cineole (**124**) (29.7%), camphor (**14**) (10.0%), *α*-pinene (**118**) (9.2%), linalool (**3**) (9.0%), selina-3,7(11)-diene (**126**) (6.6%), *Z*-*α*-bisabolene (**127**) (6.3%), borneol (**112**) (2.7%), and camphene (**15**) (2.7%) for *L. viridis*; thymol (**1**) (19.8%), *ρ*-cymene (**11**) (16.9%), 1,8-cineole (**124**) (6.6%), caryophyllene oxide (**38**) (5.7%), linalool (**3**) (5.4%), Δ-3-carene (**119**) (4.3%), *α*-terpineol (**111**) (3.6%), myrcene (**61**) (3.4%), *E*-caryophyllene (**22**) (2.4%), and *trans*-sabinene hydrate (**17**) (2.3%) for *L. graveolens*; pulegone (**128**) (74.8%), isomenthone (**129**) (10.6%), and limonene (**8**) (5.4%) for *M. cervina*; menthol (**130**) (44.0%), menthofuran (**131**) (10.9%), menthone (**132**) (9.8%), menthyl acetate (**133**) (7.8%), 1,8-cineole (**124**) (5.8%), *neo*-menthol (**134**) (4.0%), *neo*-isomenthol (**135**) (2.9%), and pulegone (128) (2.4%) for *M. x piperita*; carvacrol (**10**) (68.2%), *γ*-terpinene (**64**) (7.9%), *p*-cymene (**11**) (7.4%), *β*-myrcene (**61**) (2.4%), and thymol (**1**) (2.1%) for *O. virens*; *β*-myrcene (**61**) (32.0%), 1,8-cineole (**124**) (13.7%), camphor (**14**) (11.9%), *α*-pinene (**118**) (11.1%), limonene (**8**) (6.6%), *ρ*-cymene (**11**) (3.8%), camphene (**15**) (3.4%), and linalool (**3**) (2.1%) for *R. officinalis*; *α*-pinene (**118**) (27.4%), *β*-pinene (**39**) (16.0%), limonene (**8**) (10.0%), *γ*-terpinene (**64**) (9.3%), (*Z*)-*β*-ocimene (**89**) (8.0%), *β*-myrcene (**61**) (3.0%), camphene (**15**) (2.1%), and sabinene (**17**) (2.0%) for *S. tortuosum*; eugenol (**2**) (85.3%) and humulene (**136**) (6.8%) for *S. aromaticum*; carvacrol (**10**) (74.6%), *p*-cymene (**11**) (5.5%), *E*-caryophyllene (**22**) (3.9%), *γ*-terpinene (**64**) (3.6%), and linalool (**3**) (2.8%) for *T. capitata*; 1,8-cineole (**124**) (58.6%), borneol (**112**) (10.0%), camphene (**15**) (6.5%), *α*-pinene (**118**) (4.5%), sabinene (**17**) (3.0%), and *β*-pinene (**39**) (2.0%) for *T. capitellatus*; 1,8-cineole (**124**) (67.4%), linalool (**3**) (4.3%), *β*-pinene (**39**) (4.0%), *α*-terpineol (**111**) (3.5%), *α*-pinene (**118**) (3.0%), and sabinene (**17**) (2.4%) for *T. mastichina*; and geranyl acetate (**41**) (44.5%), geraniol (**6**) (33.1%), and camphor (**14**) (3.9%) for *T. zygis sylvestris* (Figure 27).

Of all these EOs, noteworthy findings were as follows: *Jo*EO berries (IC_50_ = 0.9 *μ*g/mL and SI = 63.4), *Cc*EO (IC_50_ = 3.2 *μ*g/mL and SI = 9.0), and *Ll*EO (IC_50_ = 5.7 *μ*g/mL and SI = 11.9). Some pure compounds were also evaluated, and the best IC_50_ values were obtained with *α*-pinene (**118**) (IC_50_ = 2.9 *μ*g/mL) and citral (**63**) (IC_50_ = 18.9 *μ*g/mL). *α*-Pinene (**118**) is the main component of the most active EO (*Juniperus oxycedrus*), contributing to its high anti-*T. brucei* activity. Because the same result was not observed with *Jo*EO leaves, the authors speculated that the synergistic effects on the most promising species potentiated their effects, suggesting that their interactions with germacrene D (**24**) and *β*-myrcene (**61**) should be further investigated [88].

**Figure 28:**
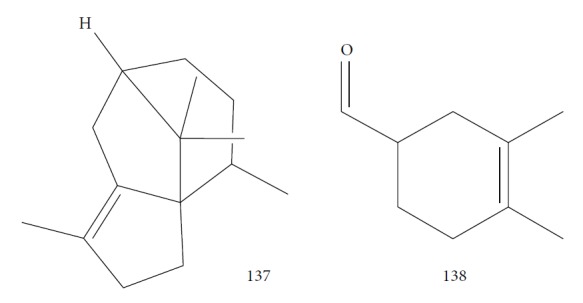
Chemical structure of cyperene (**137**) and 3,4-dimethylcyclohex-3-ene-1-carbaldehyde (**138**) – some main components identified from *Aframomum sceptrum* EO.

Cheikh-Ali et al. [133] evaluated the EO from *Aframomum sceptrum* rizhomes. *As*EO consists of 75 compounds, including *β*-pinene (**39**) (12.71%), caryophyllene oxide (**38**) (10.03%), cyperene (**137**) (5.99%), 3,4-dimethylcyclohex-3-ene-1-carbaldehyde (**138**) (3.40%), *α*-pinene (**118**) (2.78%), *β*-caryophyllene (**22**) (2.33%), *α*-terpineol (**111**) (2.12%), *D*-limonene (**8**) (1.89%), 1,8-cineole (**124**) (1.84%), and *α*-caryophyllene (or humulene) (**136**) (1.1%) (Figure 28). The minimum lethal concentration (MLC) against *T. brucei brucei* was determined for the EO (MLC = 1.47 mg/mL) and for some pure compounds, *β*-pinene (**39**) (MLC > 0.1 mg/mL) and caryophyllene oxide (**38**) (MLC = 0.1 mg/mL). When compared to the reference drug pentamidine (MLC = 7.4 *μ*g/mL), this EO and their components showed very good anti-*T. brucei brucei* activities.

**Figure 29:**
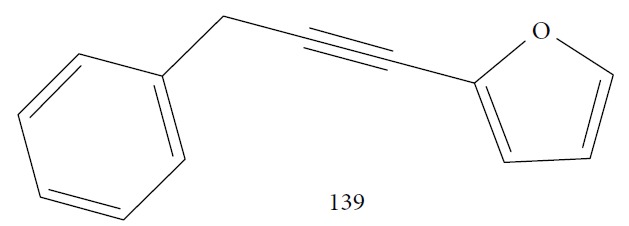
Chemical structure of carlina oxide (**139**) - main component of *Carlina acaulis* root EO.

Herrmann et al. [134] obtained the *Carlina acaulis* root EO and identified carlina oxide (**139**) (Figure 29) as its main component. Evaluation of the antitrypanosomal activity of carlina oxide against *T. b. brucei* demonstrated promising results (IC_50_ = 1.0 *μ*g/mL), with great selectivity index values (SI = 446.0). In addition to the pure compound (main component of the *C. acaulis* EO), the authors analyzed the hexane, dichloromethane, and methanol extracts, and the two former (with considerable amount of carlina oxide (**139**)) were more active (IC_50_ = 3.7 *μ*g/mL (SI = 465.5) and 4.5 *μ*g/mL (SI = 443.3), respectively) than was the latter (IC_50_ = 698.1 *μ*g/mL, SI = 1.7). When compared to the reference drug suramin (IC_50_ = 4.7 *μ*g/mL and SI = 280.2), carlina oxide (**139**) showed greater activity and better selectivity index. The authors suggested that the mechanism of action of carlina oxide (**139**) occurred through trypanothione reductase inhibition. The presence of the furan ring and the triple bond were considered by the authors to be important structural features for the activity and should be considered for the design of new drugs.

**Figure 30:**
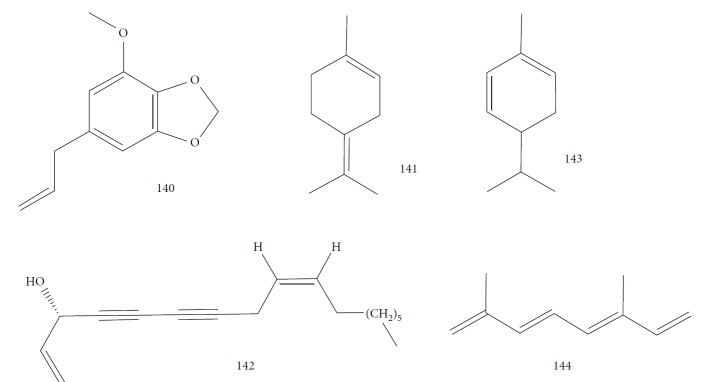
Chemical structure of myristicin (**140**), terpinolene (**141**), and (*Z*)-falcarinol (**142**) from aerial parts of *Echinophora spinosa* EO of *α*-phellandrene (**143**) and *E*,*E*-2,6-dimethyl-1,3,5,7-octatetraene (**144**) from *Crithmum maritimum* EO.

Kamte et al. [135] investigated the EOs of several herb species from the Apiaceae family from the Mediterranean zone (*Siler montanum Crantz* subsp. *siculum* – flowering aerial parts, *Sison amomum L.* – flowering aerial parts, *Echinophora spinosa L.* – roots and flowering aerial parts, *Kundmannia sicula (L.) DC. – inflorescences*, *Crithmum maritimum L.* – flowering aerial parts, *Helosciadium nodiflorum (L.) Koch –* whole, *Pimpinella anisum L.* – seeds, *Heracleum sphondylium subsp. ternatum (Velen.) Brummit* – seeds, and *Trachyspermum ammi (L.) Sprague* – seeds). The EOs from five of these plants were potent inhibitors of *T. brucei* (*Sa*EO – 4.3 ± 0.7 *μ*g/mL, *Es*EO roots – 2.7 ± 0.6 *μ*g/mL, *Es*EO aerial parts – 4.0 ± 1.6 *μ*g/mL, *Cm*EO– 5.0 ± 0.8 *μ*g/mL, and *Hn*EO – 10.7 ± 4 *μ*g/mL), and some of their main components were evaluated in isolation. The great potentiality of these EOs was attributed to the presence of the main constituents, considering as well the great importance of synergistic effects with minority constituents. For *Sa*EO, the main constituents were sabinene (**17**) (54.4%), *β*-phellandrene (**106**) (16.6%), and germacrene D (**24**) (6.7%). For *Es*EO roots, they were myristicin (41.3%) (**140**), terpinolene (**141**) (22.2%), and (*Z*)-falcarinol (**142**) (23.3%); for *Es*EO aerial parts, they were *α*-phellandrene (**143**) (47.2%), *p*-cymene (**11**) (25.6%), *β*-phellandrene (**106**) (8.3%), *E*,*E*-2,6-dimethyl-1,3,5,7-octatetraene (**144**) (6.3%), and *α*-pinene (**118**) (5.5%) (Figure 30); for *Cm*EO, they were limonene (**8**) (38.4%), *γ*-terpinene (**64**) (19.9%), and sabinene (**17**) (12.4%); and for *Hn*EO, they were myristicin (**140**) (49.1%), (*Z*)-*β*-ocimene (**89**) (19%), limonene (**8**) (7.8%), terpinolene (**141**) (7.1%), and germacrene D (**24**) (6.0%). With the exception of *Cm*EO, all other active EOs showed significant selectivities against *T. brucei*, as well as good selectivity index values. For example, in their cytotoxicity evaluation performed in Balb3T3 rat fibroblasts, they found SI = 13 for *S. amomum*, 2.1 and 3.7 for *E. spinosa* roots and aerial parts, respectively, and >9.1 for *H. nodiflorum*.

The isolated components from the EOs were also evaluated by Kamte et al. [135]. Some of these showed more activity and selectivity than did their respective EOs. The most active constituents against *T. brucei* were terpinolene (**141**) (0.035 ± 0.005 *μ*g/mL, SI = 180), *α*-pinene (**118**) (1.0 ± 0.3 *μ*g/mL, SI > 100), *β*-ocimene (**89**) (EC_50_ of 1.1 ± 0.5 *μ*g/mL, SI > 91), *p*-cymene (**11**) (EC_50_ of 4.5 ± 1.0 *μ*g/mL, SI = 6.2), limonene (**8**) (EC_50_ of 5.6 ± 1.6 *μ*g/mL, SI > 18), sabinene (**17**) (EC_50_ of 6.0 ± 1.5 *μ*g/mL, SI > 17), *α*-phellandrene (**143**) (EC_50_ of 24 ± 8 *μ*g/mL, SI = 4.8), and myristicin (**140**) (EC_50_ of 74 ± 4 *μ*g/mL, SI > 1.4). As observed for the previous values, the high anti-*T. brucei* potentiality of terpinolene (**141**) should be responsible for the great EC_50_ value found in *Es*EO roots, containing these compounds as its primary constituents.

Comparison of the chemical structure of terpinolene (**141**) with other structurally similar compounds (*γ*-terpinene (**64**), *α*-phellandrene (**143**), limonene (**8**), and *p*-cymene (**11**)) highlighted the great importance of the presence of a double endocyclic and a double exocyclic bound, the latter linking the cyclohexene ring to a dimethylated carbon for the inhibition of *T. brucei* growth. This can be used as scaffold for drug design of new anti-*T. brucei* agents.

**Figure 31:**
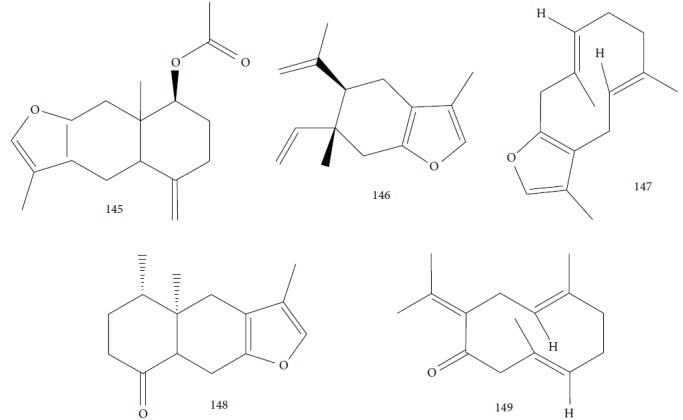
Chemical structures of *β*-acetoxyfuranoeudesm-4(15)-ene (**145**), curzerene (**146**), isofuranodiene (**147**), *β*-acetoxyfuranoeudesm-4(15)-ene (**145**), and germacrone (**149**) - main components of *Smyrnium olusatrum L.* EO.

Petrelli et al. [81] evaluated EOs of many parts of *Smyrnium olusatrum L.* (roots, leaves, flowers, and fruits), known to be rich in furan ring-containing sesquiterpenes as the main constituents. CG-MS analysis identified a domain of oxygenated sesquiterpenes (68.2% - 74.5%), followed by monoterpene hydrocarbons (13.2–22.2%) and sesquiterpene hydrocarbons (3.1-14.1%). The EO from the fruits resulted in the identification of 66 constituents, including *β*-acetoxyfuranoeudesm-4(15)-ene (**145**) (31.2%), curzerene (**146**) (23.8%), isofuranodiene (**147**) (6.6%), *β*-phellandrene (**106**) (6.2%), and *α*-pinene (**118**) (5.4%). Among the flower EOs, they found 47 compounds, including curzerene (**146**) (30.5%), myrcene (**61**) (18.2%), furanoeremophil-1-one (**148**) (12.1%), germacrone (**149**) (10.4%), and isofuranodiene (**147**) (9.8%) (Figure 31). They identified 45 compounds from roots EO, including curzerene (**146**) (39.7%), furanoeremophil-1-one (**148**) (24.4%), *β*-phellandrene (**106**) (14.4%), and isofuranodiene (**147**) (5.8%). The EO from leaves produced 43 constituents, including furanoeremophil-1-one (**148**) (30.0%), curzerene (**146**) (24.1%), germacrone (**149**) (9.7%), *β*-pinene (**39**) (9.5%), and isofuranodiene (**147**) (4.8%).

These *So*EOs showed effectiveness against *T. brucei brucei* with satisfactory SIs. The most powerful EO was obtained from fruits (IC_50_ = 1.97 ± 0.06 *μ*g/mL, SI = 29), followed by the EO from flowers (IC_50_ = 3.0 ± 0.4 *μ*g/mL, SI = 23) and from leaves (IC_50_ = 3.7 ± 0.5 *μ*g/mL, SI > 27), and at least active was the EO from roots (IC_50_ = 4.0 ± 0.5 *μ*g/mL, SI > 25). Some pure compounds were also tested, including isofuranodiene (**147**) (IC_50_ = 3.0 ± 0.8 *μ*g/mL, SI = 30), *β*-acetoxyfuranoeudesm-4(15)-ene (**145**) (IC_50_ = 26 ± 3 *μ*g/mL, SI > 3.8), and germacrone (**149**) (IC_50_ > 100 *μ*g/mL) which had the best profiles. Taking into account that curzerene (**146**) is a thermal degradation product of isofuranodiene (**147**) [136, 137], and considering the abundance of these two constituents, it may be considered that isofuranodiene (**147**) is the major component of all *So*EO and is therefore responsible for their antitrypanosomal activities. The *So*EO from fruit had as its principal component *β*-acetoxyfuranoeudesm-4(15)-ene (**145**), also contributing to its superior potential over the other components.

Structurally, the absence of the furan ring in germacrone (**149**), as opposed to isofuranodiene (**147**) and *β*-acetoxyfuranoeudesm-4(15)-ene (**145**) structures (Figure 20), can be the main explanation of its inactivity. In addition, the more potent compound isofuranodiene (**147**) was already known for its inhibitory activity against dihydrofolate reductase, an important enzyme in purine biosynthesis and therefore in DNA synthesis. This may be the mechanism of action for its inhibition of *T. brucei brucei* growth [138].

Muhd Haffiz and colleagues [121] evaluated the anti-*T. brucei brucei* activity of *Cymbopogon nardus L.* (whole plant) EO and fractions, using pentamidine (IC_50_ = 0.00438 *μ*g/mL, SI = 4.29) as the standard drug. CG-MS revealed that the majority of the constituents of *Cn*EO were oxygenated monoterpenes (79%), sesquiterpenes (12.3%), oxygenated sesquiterpenes (2.6%), and monoterpene hydrocarbons (1.9%), including citronellal (**62**) (35.5%), geraniol (**6**) (28%), and citronellol (**51**) (11%). In the biological evaluation, *C. nardus* EO exhibited strong antitrypanosomal activity and high SI (IC_50_ = 0.31 ± 0.03 *μ*g/mL, SI > 323).

**Figure 32:**
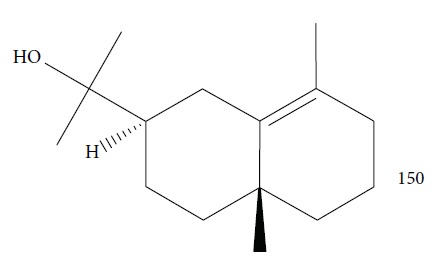
Chemical structures of *γ*-eudesmol (**150**) – one of the main constituents from *Cymbopogon nardus* EO.

These EOs were fractionated into 7 fractions, then combined, and then refracted into 8 subfractions, and finally their activities were determined. In the great majority of cases, the fractions and subfractions showed great potential for inhibition of *T. b. brucei* growth with low cytotoxicity. The most active fractions were as follows: subfraction **4**′ (IC_50_ = 0.61 ± 0.06 *μ*g/mL, SI = 127), subfraction **6**′ (IC_50_ = 0.73 ± 0.33 *μ*g/mL, SI ≥ 137), subfraction **7**′ (IC_50_ = 1.15 ± 0 *μ*g/mL, SI = 87), and subfraction **8**′ (IC_50_ = 1.11 ± 0.01 *μ*g/mL, SI ≥ 90). The major constituents were *γ*-eudesmol (**150**) (Figure 32) and *α*-cadinol (**53**) for subfractions **4**′ and **6**′ and elemol (**52**) for subfractions **7**′ and **8**′.

Essential oils from aromatic and medicinal plants from Cameroon with known antitrypanosomal properties were evaluated by Kamte et al. [18]. The authors identified chemical compositions and evaluated the anti-*T. brucei* potential of the EOs and constituents from *Azadirachta indica* (leaves), *Aframomum melegueta* (seeds), *Aframomum daniellii* (leaves), *Clausena anisata* (leaves), *Dichrostachys cinerea* (seeds), and *Echinops giganteus* (roots).

**Figure 33:**
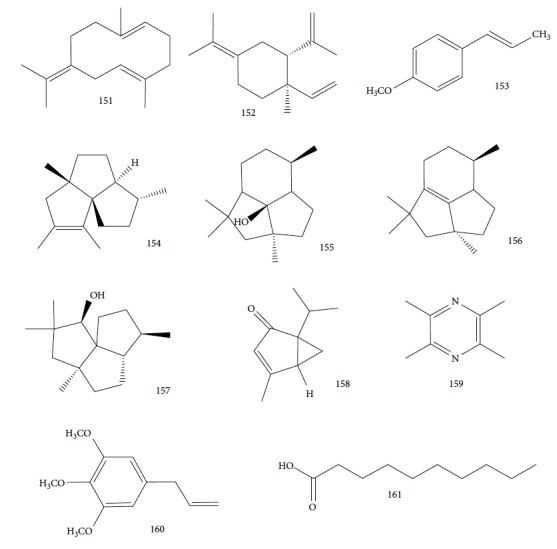
Chemical structures compounds isolated of aromatic and medicinal plants from Cameroon.

For *Ai*EO, 13 compounds were identified, with abundance of sesquiterpene hydrocarbons (97.4%), especially germacrene B (**151**) (74.0%) and *γ*-elemene (**152**) (18.3%) (Figure 33); for *Am*EO, 59 compounds were identified, with abundance of oxygenated monoterpenes (83.3%), of which 1,8-cineole (**124**) (58.5%), *α*-terpineol (**111**) (19.4%), and *β*-pinene (**39**) (7.1%) were the main constituents; for *Ad*EO, 57 compounds were identified, with greater abundance of monoterpene hydrocarbons (59.8%), where sabinene (**17**) (43.9%), (*E*)-caryophyllene (**22**) (16.6%), and *β*-pinene (**39**) (5.8%) were the most abundant; for *Ca*EO, 47 compounds were identified, with abundance of phenylpropanoids (84.0%), where (*E*)-anethole (**153**) (64.6%) and (*E*)-methyl isoeugenol (**2**) (16.1%) (Figure 33) were the main constituents; for *Eg*EO, 35 compounds were identified, with abundance of sesquiterpenes (94.3%-54.7% of sesquiterpene hydrocarbons and 39.6% of oxygenated sesquiterpenes), where silphiperfol-6-ene (**154**) (23.0%), presilphiperfolan-8-ol (**155**) (22.7%), presilphiperol-7-ene (**156**) (7.8%), cameroonan-7-*α*-ol (**157**) (7.1%), and caryophyllene (**22**) (6.3%) were the most abundant (Figure 33); and for *D. cinereal*, 49 compounds were identified, and the class of oxygenated monoterpenes (50.6%) was the primary, and geraniol (**6**) (18.2%), terpinen-4-ol (**103**) (7.5%), linalool (**3**) (4.0%), and umbellulone (**158**) (3.8%) were the most representative constituents. Representative amounts of other classes of compounds were also observed, especially ligustrazin (**159**) (5.1%), elemicin (**160**) (3.0%), and decanoic acid (**161**) (2.8%) (Figure 33).

The *in vitro* evaluation of these EOs demonstrated inactivity of *Dc*EO, *Am*EO, and *Ca*EO (IC_50_ > 100 *μ*g/mL), and moderate activity of *Ad*EO (IC_50_ = 7.65 ± 1.1 *μ*g/mL), *Eg*EO (IC_50_ = 10.50 ± 1.7 *μ*g/mL), and *Ai*EO (IC_50_ = 15.21 ± 0.97 *μ*g/mL), when compared to standard drug suramin (IC_50_ = 0.0286 ± 0.0008 *μ*g/mL). The SIs of the three more active EOs were also evaluated and were >13.1, >9.52, and >6.57, respectively [18].

Some of the main constituents of *A. melegueta* and *A. daniellii* EOs were also evaluated. 1,8-Cineole (**124**) and terpinen-4-ol (**103**) (main compounds of *Am*EO) were inactive (IC_50_ > 100 *μ*g/mL), while sabinene (**17**) (IC_50_ = 5.96 ± 1.3 *μ*g/mL), (*E*)-caryophyllene (**22**) (IC_50_ = 8.25 ± 1.3 *μ*g/mL), and *β*-pinene (**39**) (IC_50_ = 11.4 ± 2.6 *μ*g/mL), main components of *Ad*EO, showed moderate to good anti-*T. brucei* activities [18].

### 1.3. Essentials Oils Active against Leishmaniasis

Leishmaniasis is a group of noncontagious infectious diseases caused by at least 20 parasitic protozoa belonging to the family Trypanosomatidae (genus *Leishmania*). It is estimated that at least 12 million people in more than 100 countries are infected, and at least another 350 million people are at risk in endemic areas [139, 140]. This set of diseases can be divided into two major groups of distinct diseases, tegumentary leishmaniasis (subdivided into cutaneous, diffuse cutaneous, and mucosal), and visceral leishmaniasis, usually more severe, with high mortality [139, 141].

The life cycle of *Leishmania* sp. begins with the blood supply of a phlebotomine that infects itself through the blood of an infected mammalian, ingesting amastigote forms of the parasite that are internalized in macrophages. In the intestine of the vector-insect, the amastigote form becomes a metacyclic promastigote form. When the insect is fed again and it bites a new mammalian, virulent promastigote forms are inoculated in the new host. In the bloodstream, they are internalized by macrophages, differentiating again into amastigotes, thus completing the cycle [140, 141].

The few therapeutic options available for treatment include a class of drugs that is more than 60 years old, the pentavalent antimonials (meglumine antimoniate and sodium stibogluconate). More recently, for the treatment of refractory and resistant cases, pentamidine, miltefosine, amphotericin B, and paromomycin are used [140].

In the same way as observed for the trypanosomiases described above, the scarcity of drugs for leishmaniasis necessitated the use of plants, and parts of plants, as therapeutic alternatives in several parts of the world.

Between 2000 and 2018, the literature reported the use of EOs from 142 plant species (30 families) and 44 isolated compounds that were evaluated against various *Leishmania* species (*Leishmania amazonensis*, *Leishmania braziliensis*, *Leishmania aethiopica*, *Leishmania donovani*, *Leishmania chagasi*, *Leishmania infantum*, *Leishmania guyanensis*, *Leishmania panamensis*, *Leishmania mexicana*, *Leishmania tropica*, *and Leishmania major*) and forms (promastigote, axenic amastigote, and intracellular amastigote).

**Figure 34:**
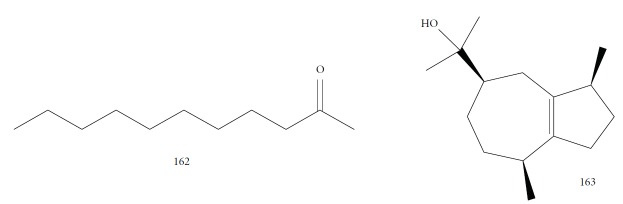
Chemical structures of some compounds strongly active (nanomolar concentration) against Leishmania species.

The most studied families were Lamiaceae, Piperaceae, and Asteraceae, with 35, 29, and 14 species evaluated, respectively. Fifty-one of the EOs (especially from plant species the families Lamiaceae and Asteraceae) and 13 isolated compounds showed IC_50_ values less than or equal to 10 *μ*g/mL against at least one species of *Leishmania* (promastigote or amastigote forms) and were considered active based on hit selection criteria for visceral leishmaniasis [142]. In addition, four EOs and one isolated compound showed quantified activity in nanomolar concentrations, considered strongly active (Tables 1 and 2 and Figure 34) (see supplementary material Figures S1 and S2). In our review, some of the most active EOs and pure compounds were detailed for their major constituents (for EOs), efficacy (IC_50_ values), selectivity index (SI), and morphological and ultrastructure alterations in the parasite, as well as for their mechanism of action. For inactive extracts and chemical entities (IC_50_ > 50 *μ*g/mL), and for those in which only the parasite was sensitive (moderate activity; 50 < IC_50_ > 10 *μ*g/mL), see supplementary material (Table S1).

**Figure 35:**
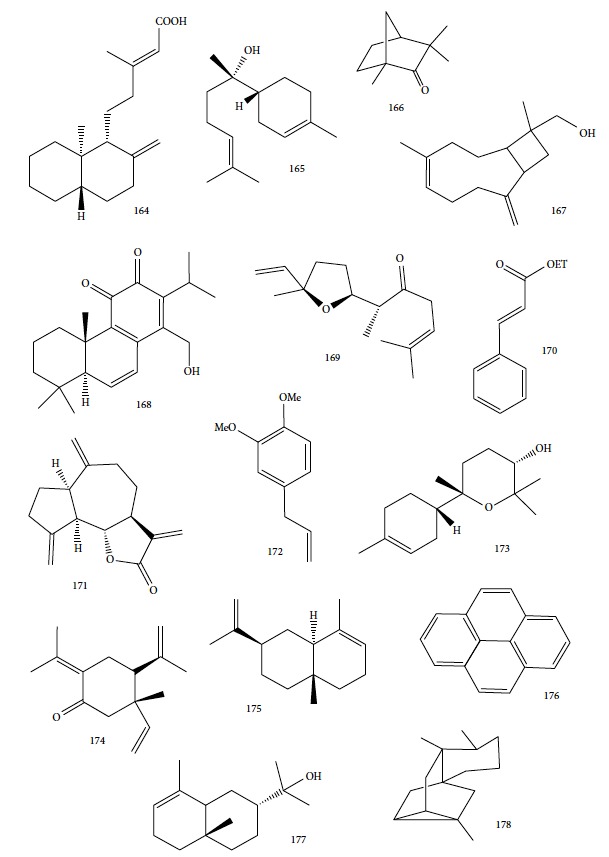
Chemical structures of 2-undecanone (**162**) from *Ruta chalepensis* EO and guaiol (**163**) from *Bulnesia sarmientoi* EO actives against Leishmania species.

EOs from 10 plants issued from the Sned region of Tunisia, an endemic region for various forms of *Leishmania*, showed antileishmanial activities against *L. major* and *L. infantum* promastigote forms. In general, for all tested extracts, *L. infantum* species were more sensitive than were *L. major*, and only two EOs were more effective against the parasites than were murine macrophagic cells. EOs from *Thymus hirtus* sp. *Algeriensis*, rich in oxygen-containing monoterpenes, especially linalool (**3**) (17.62%) and camphor (**14**) (13.82%), and from *Ruta chalepensis*, rich in 2-undecanone (**162**) (Figure 35) (84.28%), showed IC_50_ against *L. infantum* promastigote forms of 0.25 and 1.13 *μ*g/mL, respectively [143]. In addition, only *Thymus hirtus sp. Algeriensis* EO showed leishmanicidal activity against *L. major* (IC_50_ = 0.43 *μ*g/mL). On the other hand, for these two EOs, SI values (calculated as IC_80_/IC_50_) ranging from 0.19 to 1.57 showed inadequate antileishmanial activity by hit selection [143]. Two other EOs (from *T. articulata* and *Lavandula multifida*) were inactive against *L. major* promastigotes, and for the remaining six EOs, IC_50_ values ranged from 0.30 to 2.23 *μ*g/mL, but with high levels of cytotoxicity (SI < 1) [143].

EOs from sixteen plant species belonging to various families, purchased from QUINARI Cosmetic and Fragances Inc. (Maringá, Pr, Brazil) or collected in Lavras, MG (Brazil), were evaluated for their biological potential against *L. amazonensis* promastigote forms after 24 h of exposure [144]. None of the EOs tested showed desirable IC_50_ values. For the four most effective EOs (from *Matricaria chamomilla*, *Cordia verbenaceae*, *Siparuna guianensis*, and *Cinnamodendron dinisii* species), the IC_50_ values ranged from 48.55 to 64.75 *μ*g/mL and were considered moderately active or inactive. Furthermore, for all EOs tested, SI were not satisfactory (0.99 < SI > 3.94). From the chemical composition of the evaluated EOs and the IC_50_ and SI values, the authors compared these leishmanicidal activities and cytotoxicities with those of EOs from other plant species. Literature reports that EOs from *Matricaria chamomilla* showed no leishmanicidal activity against *L. donovani* specie. Another EO from *Lantana camara*, with similar major constituents (farnesene (**117**) derivatives as major constituents), showed significant leishmanicidal activity against *L. amazonensis* (IC_50_/72 h = 0.25 *μ*g/mL), but with high cytotoxicity [145, 146]. In another example, EO from *Bulnesia sarmientoi*, with 48.29% of guaiol (**163**) (Figure 35) as its major constituent, was inactive against *L. amazonensis* (IC_50_ = 85.56 *μ*g/mL). However, EO from *Endlicheria bracteolate*, with 72.12% of guaiol (**163**), had an IC_50_ of 7.93 *μ*g/mL against the same *Leishmania* species and a similar value of SI (1.91) [147]. Finally, they observed that the concentration of linalool (**3**) in the EO from *Salvia sclarea* was insufficient to promote leishmanicidal activity for this EO, since Kuźma et al. demonstrated that linalool-rich EO from the leaves of *Croton cajucara* promoted morphological changes in *L. amazonensis* promastigotes treated at concentrations of 15 ng/mL [148].

In order to explore ethnopharmacological data of copaiba oils obtained from Peru, Maranhão, and Amazonas (states in Brazil) and French Guiana which are used in folk medicine to treat leishmaniasis [149–152], Santos and coworkers investigated the antiproliferative effect of EOs from eight kinds of Brazilian copaiba against promastigote and amastigote forms of *L. amazonensis* [153]. All copaiba oils showed some level of activity with IC_50_ values ranging between 5.0 and 22.0 *μ*g/mL. The most active EO (from *Copaifera reticulata*, IC_50_ = 5.0 *μ*g/mL) demonstrated effects against axenic and intracellular amastigote forms, with IC_50_ values of 15.0 and 20 *μ*g/mL, respectively. With respect to cytotoxicity, this same EO showed a tolerable value of SI (8.0) to promastigote forms and an inadequate value (2.5) to axenic amastigote forms. Nevertheless, studies revealed that copaiba oil from *Copaifera reticulata* did not alter the viability of peritoneal macrophages at a concentration of 500 *μ*g/mL, and the treatment at 500 mg/kg with EOs from *Copaifera reticulata* and *Copaifera multijuga* did not induce behavioral alterations, lesions, or bleeding in the stomachs of mice [154, 155].

These results involving copaiba EOs from various species led the same authors to investigate the *in vivo* antileishmanial activity against *L. amazonensis* of an EO from *Copaifera martii*, also using electron microscopy, biochemical analysis, and flow cytometry [156]. Infected mice were treated with copaiba oil for 30 days. After oral treatment (100 mg/kg/day), oral and topical (1 mg/mm^2^) treatments reduced the average lesion size in relation to the control group (75% and 72.5% reduction, respectively); however, no significant difference was observed when compared to the reference drug glucantime (79.5% reduction). Nevertheless, topical and subcutaneous (100 mg/kg/day) treatments did not show significant reduction in average lesion size, and the subcutaneous treatment caused injuries to the animal.

Histopathological and mutagenicity evaluations showed no genotoxic effects or changes in various organs in animals treated with copaiba EO compared with control animals. In addition, the following microscopic aspects of the parasite were observed: (i) promastigote forms of *L. amazonensis* treated with concentrations of 14 *μ*g/mL (IC_50_) and 70 *μ*g/mL (IC_90_) showed ultrastructural changes and swollen mitochondria, and (ii) an increase in plasma membrane permeability and depolarization of the mitochondrial membrane potential were observed in axenic amastigotes treated with 100 and 200 *μ*g/mL (Figure 1). These results supported the notion that copaiba oil may be the source of a new drug; however, its mechanism of action still needs to be clarified [156].

The antileishmanial effects of copaiba EOs from *Copaifera reticulata* and *Copaifera martii* species against *L. amazonensis* led another research group to investigate the biological potential of four commercial oils from *Copaifera* ssp. C1-C4, *β*-caryophyllene-, sesquiterpene- and diterpene-rich fractions were obtained from distillation [157]. Diterpene-rich EOs (C2 and C3) were more active against *L. amazonensis* promastigotes. Sesquiterpene-rich EOs C1 and C4 were more potent against intracellular amastigotes, with IC_50_ of 2.9 and 2.3 *μ*g/mL, respectively. In comparison with the same activity of EO from *Copaifera reticulata* [153], C1 and C4 EOs were 6.9- and 8.7-fold more potent, respectively. For all the compared EOs containing *β*-caryophyllene (**22**) as their major constituent, the good IC_50_ value (1.3 *μ*g/mL or 6.4 *μ*M) demonstrated by the isolated sesquiterpene suggested that this compound may directly influence the antileishmanial activity of the EOs assayed. In addition, SI values of 29.3, 40.1, and 48.9 for C1, C4, and *β*-caryophyllene (**22**), respectively, demonstrated a better ratio of efficacy to toxicity. Finally, the C4 sesquiterpene-rich fraction showed a similar antileishmanial activity against amastigote forms, probably due to the same concentration of *β*-caryophyllene (**22**) in both the EO and the fraction, and C4 diterpene-rich fraction was not active in low concentrations [157].

EOs from four plant species reported in traditional medicine in Ethiopia, *Artemisia absinthium* L., *Artemisia abyssinica*, *Echinops kebericho* MESFIN, and *Satureja punctata*, were tested against promastigote and axenic amastigote forms of *L. donovani* and *L. aethiopica*. The results demonstrated that two EOs from *Artemisia absinthium* L. and *Echinops kebericho* MESFIN possessed strong antileishmanicidal activity against promastigote (IC_50_ ranging from 0.24 to 42.00 *μ*g/mL) and axenic amastigote forms (IC_50_ ranging from 0.0097 to 0.15 *μ*g/mL) of both *Leishmania* species_._ In relation to the other two species tested, *Artemisia abyssinica* was inactive against the two forms of parasite, and *Satureja punctata* showed high cytotoxicity. Unfortunately, the great number of constituents (including mono- and sesquiterpenes, aliphatic and aromatic hydrocarbons, carotenoid derivatives, and oxygenated structures) that appeared to have no specific cellular targets could explain the inadequate SI values for the most active oils [158, 159].

In a recent study, Silva and colleagues evaluated the antileishmanial activity of the EO from leaves of *Ocimum canum* Sims. that is commonly used as an infusion or syrup for the treatment of a number of diseases. The biological results revealed that *Oc*EO was rich in monoterpenes such as thymol (**1**) (42.15%), p-cymene (**11**) (21.17%), and *γ*-terpinene (**64**) (19.81%), demonstrating moderate activity against promastigote and intracellular amastigote forms of *L. amazonensis*, with IC_50_ values of 17.4 and 13.1 *μ*g/mL, respectively. These values were more potent than those of the reference drug pentamidine, with satisfactory SI values (18.1 and 24.0, respectively) [157]. Electron microscopy showed that *Oc*EO was associated with several ultrastructural alterations in promastigote forms of the parasite, including autophagosome-like structures that were multivesicular and had lipid bodies that could result in cell death, discontinuity of the nucleus membrane, and exocytic activity by the flagellar pocket [160].

EO from leaves and flowers of *Achillea millefolium*, species used as antihelmintic and trypanocidals, was evaluated *in vitro* against *L. amazonensis*. Significant activity was observed against promastigote and intracellular amastigote forms, with IC_50_ values of 7.8 and 6.5 *μ*g/mL and acceptable SI values of 9.2 and 11.0, respectively [161]. Unfortunately, the major constituents of *Am*EO were not determined. *Scanning electron microscopy* (SEM) and transmission *electron microscopy* (TEM) analysis of the treated promastigote forms revealed morphological alterations in the shape and size of the parasite as well as ultrastructural alterations, including changes in the flagellar membrane, abnormal membrane structures, rupture of the plasma membrane, atypical vacuoles, myelin-like figures, and vesicles that resembled autophagic vacuoles. The mechanism of action remains unclear; however, the morphological changes observed in the parasite may be related to inhibition of two parasite enzymes: serine-protease and squalene synthase (BPQ-OH) [161].

Rosa et al. investigated the leishmanicidal effects of linalool-rich EO from leaves of *Croton cajucara* and purified linalool (**3**) [162]. Both EO and purified linalool (**3**) showed potent antileishmanial activity, with IC_50_ values of 8.3 and 4.3 ng/mL against promastigote forms and values of 22.0 and 15.5 ng/mL against the amastigote form of the parasite, respectively. SI values were not determined, but for both, the concentration of 15.0 ng/mL presented no cytotoxic effects against mammalian cells. Mitochondrial swelling and important alterations in the organization of the nuclear and kinetoplast chromatins decrease in the association between macrophages and parasites, and increases in nitric oxide levels in infected macrophages could explain the extreme toxicity of the *Cc*EO for *L. amazonensis* [162].

Some studies reported that linalool (**3**) (oxygenated monoterpene) and eugenol (**2**) (phenolic compound), chemical constituents in many EOs from various plant species, as well as eugenol- and linalool-rich EOs, were fatal for several species of *Leishmania* [162–165]. Dutra and coworkers explored the biological potential of these purified compounds in *L. infantum chagasi* promastigotes and axenic amastigote forms at baseline and during the infection of peritoneal mouse macrophages [166]. The estimated IC_50_ values for eugenol (**2**) and linalool (**3**) against axenic amastigotes forms were 220 and 550 *μ*g/mL, respectively. The IC_50_ value for eugenol (**2**) against promastigote forms was 500 *μ*g/mL, and for linalool (**3**), the IC_50_ value for the last parasite form was not determined. For both compounds, some of the mechanisms associated with leishmanicidal activity were confirmed: the two derivatives were able to enhance the activities of protein kinases PKC and PKA, linalool (**3**) decreased parasite oxygen consumption, and eugenol (**2**) reduced the parasite resistance to reactive oxygen species [166].

Santim et al. (2009) evaluated the effect of EO from *Cymbopogon citratus* and its major constituent, citral (**63**), against *L. amazonensis*. The EO showed good activity against promastigote and amastigote forms, with IC_50_ values of 1.7 and 3.2 *μ*g/mL, respectively, a good SI value (14.7) for promastigotes and a tolerable value IC_50_ of 8.7 *μ*g/mL against promastigote forms, with a tolerable SI of 6.3. SEM and TEM analysis revealed aberrant morphologies of the amastigote forms exposed to the EO, including disintegration of the parasite, mitochondrial swelling, exocytic projections in the flagellar pocket, swollen mitochondria, and rupture of the plasma membrane, characterized by the presence of extracellular materials [167].

Rodrigues and colleagues [139, 169] evaluated the antileishmanial potential of EOs from *Eugenia uniflora* L. and *Syzygium cumini* (L.) Skeels, and *α*-pinene (**118**), major constituents of *Eu*EO. *α*-Pinene (**118**) was more effective than was the EO from the plant, without desirable potency (IC_50_ ≥ 38.1 *μ*g/mL against various forms of the parasite). The isolated compound showed moderate IC_50_ values (16.1 and 15.6 *μ*g/mL against axenic and intracellular amastigote forms, respectively) and low cytotoxicity (SI > 21.5). The mechanism of action for monoterpene was mediated by immunomodulatory activity (increase in phagocytic and lysosomal activities) and elevated NO levels. However, EO from *E. uniflora* showed significant antileishmanial activity, with IC_50_ values of 3.04 and 1.92 *μ*g/mL against promastigote and amastigote forms, respectively, and good SI values of 14.9 and 23.9, respectively. In relation to the mechanism of action, this EO activity was not mediated by nitric oxide production, suggesting that macrophage activation may be involved in the biological activity, as demonstrated by increases in both phagocytic capacity and lysosomal activity [168, 169].

Bosquiroli et al. evaluated the antileishmanial activity of EO from *Piper angustifolium*. *Pa*EO rich in sesquiterpenes such as spathulenol (**108**) (23.78%) and caryophyllene oxide (**38**) (13.06%) reduced the number of intracellular amastigotes of *L. infantum* with an IC_50_ value of 1.43 *μ*g/mL. It was more toxic to NIH/3T3 fibroblasts and murine macrophage J774.A1, with good SI values of 33.72 and 22.5, respectively. For *Pp*EO, the mechanism of action may be associated with increased NO release after treatment at low concentrations [170].

EOs obtained from the aerial parts of four Moroccan plant species (Lamiaceae) were screened against various *Leishmania* species [171–173]. The EOs from *Lavandula stoechas*, with fenchone (**166**) (31.81%), camphor (**14**) (29.60%), and terpineol (**111**) (13.1%) as major constituents, exhibited strong leishmanicidal effects against two *Leishmania* species, whereas *L. major* was the most sensitive strain with an IC_50_ of 0.9 *μ*g/mL, followed by *L. infantum* (IC_50_ = 7.0 *μ*g/mL) and *L. tropica* (IC_50_ > 10 *μ*g/mL). For the EO from *Origanum compactum*, three phenological stages of the plant were analyzed, and the oxygenated monoterpenes were the major constituents at the three phenological stages (49.4% at vegetative stage, 62.975% at flowering stage, and 61.379% at postflowering stage). In lower quantities, other chemical classes such as sesquiterpene hydrocarbons, oxygenated sesquiterpenes, alcohols, ketones, and acids were also identified. At low concentrations, these EOs showed significant cytotoxicity against three *Leishmania* species, while the cytotoxic effects are not significantly variable with increasing concentrations. The *L. infantum* species was the most sensitive strain, with IC_50_ values ranging from 0.02 to 0.12 *μ*g/mL at the three phenological stages. The other two EOs from *Mentha pulegium* and *Rosmarinus officinalis* were studied by the same authors, and both monoterpene-rich EOs were potent against *L. major*, *L. infantum*, and *L. tropica* species, showing IC_50_ values ranging from 0.36 to 2.6 *μ*g/mL. Unfortunately, for all EOs studied, the SI values were not determined, and the relationship between efficacy and toxicity could not be measured [171–173].

Essid et al. evaluated the antileishmanial activity of EOs from twelve medicinal plants from Northern Tunisia. The first group of EOs isolated from *Ferula communis*, *Teucrium polium*, and *Pelargonium graveolens* species displayed strong inhibitory activities against the promastigote forms of*L. major* and *L. infantum*, showing IC_50_ values ranging from 0.05 to 0.28 *μ*g/mL. The second group contained five EOs belonging to *Thymus hirtus*, *Artemisia campestris*, *Myrtus communis*, *Artemisia herba-alba*, and *Alvia officinalis*, exhibiting good leishmanicidal activities with IC_50_ values ranging from 1 to 10 *μ*g/mL against the same species of the parasite, but with lower activity when compared with amphotericin B. In the third group, EOs from *Nigella sativa*, *Laurus nobilis*, *Rosmarinus officinalis*, and *Eucalyptus globulus* showed IC_50_ values up to 10 *μ*g/mL. Purified *β*-caryophyllene (**22**), camphor (**14**), and carvacrol (**10**) were screened against the same parasite species and showed less activity when compared with the most active EOs classified as the first group. In addition, the most active EOs and purified compounds showed low cytotoxicity to macrophage cells, presenting ideal values of SI (SI > 10) [174].

An *in vivo* study was performed with the EO extracted from the leaves of *Artemisia annua*, which has camphor (**14**) (52.06%), *β*-caryophyllene (**22**) (10.95%), and 1,8-cineole (**124**) (5.57%) as major constituents. *Aa*EO presented an IC_50_ value of 7.3 *μ*g/mL against amastigote forms of *L. donovani*, but no cytotoxic effects against mammalian macrophages even at 200 *μ*g/mL. In addition, intraperitoneal administration of the *Aa*EO (200 mg/kg/b.w.) to infected BALB/c mice reduced the parasite burden by almost 90% in the liver and spleen, with significant reduction in weight. There was no hepatic or renal toxicity, as demonstrated by normal levels of serum enzymes [163].

Drmachi et al. (2016) evaluated the immunomodulatory effects of EO from *Tetradenia riparia* against *L. amazonensis* species. They found that *Tr*EO induced 50% death of amastigote forms of the parasite after 24 h incubation at a concentration of 30 *μ*g/mL. In addition, the oil did not present cytotoxicity in murine macrophages at the same concentration, with cell viability >95%. They evaluated the modulatory effects of *Tr*EO on cytokine levels in peritoneal fluid cells that were infected with *L. amazonensis*. They observed inhibition of some of the most critical cytokines for parasite growth and the establishment of infection, including granulocyte-macrophage colony-stimulating factor, interleukin-4 (IL-4), IL-10, and tumor necrosis factor (TNF). The parasite inhibited interferon-*γ* and IL-12, and the EO blocked this inhibition, indicating that these cytokines were critical for activating mechanisms associated with the death and elimination of the parasite [175].

EO from *Chenopodium ambrosioides*, containing carvacrol (**10**) (62.36%) and ascaridole (**37**) (22.54%) as major constituents, was investigated against *L. amazonensis*. *Ca*EO showed EC_50_ values of 3.7 and 4.6 *μ*g/mL against promastigote and amastigote forms, respectively, and cytotoxicity was approximately 15-fold higher for peritoneal macrophages than for both parasite forms [120]. For the promastigote form, they observed synergistic activity in conjunction with pentamidine [176], and other results showed similar activity against promastigote and amastigote forms of *L. donovani*, with EC_50_ values of 4.45 and 5.1 *μ*g/mL, respectively [177]. An *in vivo* study demonstrated significant reduction in the size of the lesions in animals treated with the EO during the first 15 days of treatment. They also observedsignificant suppression of the number of parasites in the infected footpads, as compared with the burden in the footpads of the other treated mice, and oral administration at a dose of 30 mg/kg slowed the infection in an experimental model [178].

In a subsequent study, the authors observed a significant reduction in lesion size in BALB/c mice treated with the EO from *C. ambrosioides* compared with untreated animals at doses of 30 and 150 mg/kg by intralesional and oral routes. This activity was superior to that of the reference drugs glucantime, amphotericin B, and pentamidine, or the two major constituents carvacrol (**10**) and ascaridole (**37**) [179, 180]. This ability to control disease progression of cutaneous leishmaniasis was also observed with EO from *Bixa orellana*, at dose of 30 mg/kg over 14 days [181]. Finally, recent studies suggested that *Ca*EO caused a breakdown of mitochondrial membrane potential and a modification of redox indexes [64], and the isolated compounds carvacrol (**10**), ascaridole (**37**), and caryophyllene oxide (**38**) mediated their leishmanicidal activity via various mitochondrial targets, including the electron transport chain, thiol depletion, and production of superoxide radical, causing significant impairment of mitochondrial coupling [182].

The chemical analysis of EO from *Vanillosmopsis arborea* Baker showed that *α*-bisabolol (**165**) was its main constituent (97.9%). Both *Va*EO and *α*-bisabolol (**165**) efficiently inhibited the growth of *L. amazonensis* promastigotes, with IC_50_ values of 7.35 and 4.95 *μ*g/mL, respectively [183]. In addition, *α*-bisabolol (**165**) was more effective against promastigotes and intracellular amastigotes, and the cytotoxic assay against intracellular amastigotes showed that the pure compound (SI = 9.38) was less toxic than was *Va*EO (SI = 11.52). These results suggested that both *α*-bisabolol (**165**) and *Va*EO increased cell permeability to exogenous compounds, since some sesquiterpenes can induce changes in membranes, allowing microorganisms to enter the cells, thus augmenting the microbial permeability to antimicrobial agents. The ultrastructural analysis of the parasite showed an elongated cell body and the presence of a well-defined kinetoplast and nucleus. The promastigotes treated with *α*-bisabolol (**165**) for 24 hours at 30 *μ*g/mL showed severe cell damage with loss of parasite morphology, discontinuity of the nuclear membrane, increased mitochondrial volume and kinetoplast, and presence of vesicles with an electrondense display with lipid inclusion in the plasma membrane. However, in the same conditions of analysis for *Va*EO, they observed increased volumes of flagellar pockets with consequent breakage, increased volumes and changes in mitochondrial kinetoplasts, abnormal condensation of chromatin in the nucleus, discontinuity of the nuclear membrane, lipid inclusions in the presence of electrondense vesicles, and visualization of the inclusion of a lipid envelope within the plasma membrane, with the consequent loss of parasite morphology [183].

**Figure 36:**
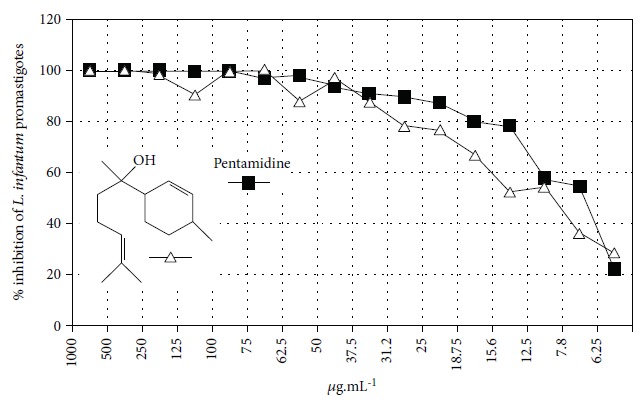
Percentage inhibition of *L. infantum* promastigotes for (−)*α*-bisabolol (**165**) in comparison with pentamidine.

Another group evaluated the effect of (−)-*α*-bisabolol (**165**) against promastigote forms of *L. infantum*. In general, this sesquiterpene showed a very similar percentage of inhibition of the parasite when compared with the reference drug pentamidine (Figure 36). The value of IC_50_ for the compound was 10.99 *μ*g/mL, and the presence of a hydroxyl in its structure explains its high capacity to inhibit *L. infantum* promastigote form. In addition, the very low toxicity *in vivo* supported the therapeutic use of (−)-*α*-bisabolol (**165**) to treat leishmaniasis caused by *L. infantum* [184, 185].

**Figure 37:**
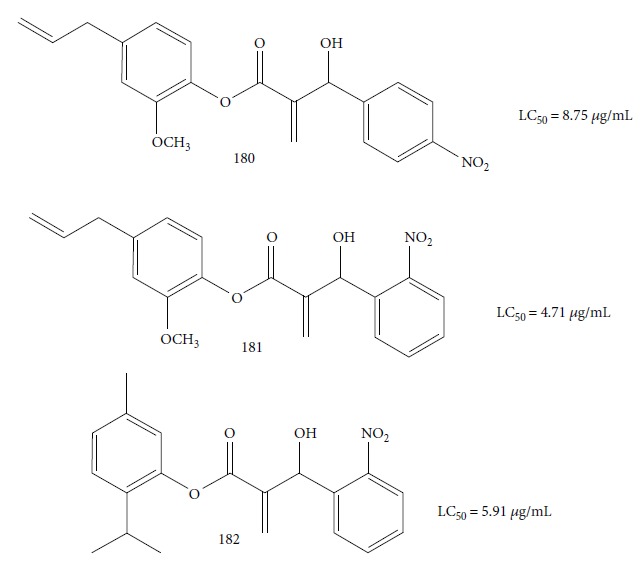
MBHA hybrids from eugenol, thymol, and carvacrol, chemical structures and IC_50_ values of the most potent derivatives against of *L. amazonensis* promastigote form.

Xavier et al. explored the leishmanicidal properties of synthesized compounds derived from the oxygenated monoterpenes eugenol (**2**), thymol (**1**), and carvacrol (**10**), by molecular hybridation with nitrated Morita-Baylis-Hillman adducts (MBHAs) [184]. The general structure and IC_50_ values of the most potent hybrid compounds against *L. amazonensis* promastigote forms are shown in Figure 37. Hybrid compounds **180**, **181**, and **182** exhibited the best antileishmanial activity, with IC_50_ values ranging from 4.71 to 8.75 *μ*g/mL, and SI > 45 (except for derivative **3**, which was not determined). In addition, the compounds substituted with nitro groups at the *ortho* position were more bioactive than the *meta* and *para* nitroaryl isomers. Experimental and in silico studies indicated that the mechanism of action was connected to nitro group reduction that generated RNO^−•^ [186].

Ten chemical constituents of EOs, including nine monoterpenes and one phenylpropanoid, were investigated as antileishmanial agents against promastigote forms of *L. amazonensis* [187]. Among the monoterpenes evaluated, the positional isomers carvacrol (**10**) and thymol (**1**) that presented a phenolic moiety were the most active compounds, showing moderate antiparasitic activity, with IC_50_ values of 25.4 and 26.8 *μ*g/mL, respectively. This result suggests that the position of the hydroxyl group in the aromatic ring did not influence the antileishmanial activity. For eugenol (**2**), a hydrogen bond between hydroxyl and *o-*methoxyl groups reduced the release of protons by the OH group, and the acidity of this group may play an important role in the potency of the compounds. In addition, for this series of compounds, the absence of aromatic hydroxyls caused compounds to be less potent, as observed for *p*-cymene (**11**) and menthol (**130**) with IC_50_ values of up to 198.9 *μ*g/mL.

In the same study, the IC_50_ value found for carvacrol (**10**) was different from values reported in the literature for *L. chagasi* and *L. amazonensis* promastigotes (IC_50_ = 2.3 [56] and 28.0 [56], and 15.3 *μ*g/mL [188], respectively). Drug-parasite incubation (24-72 h) and assay methods may explain these differences between values of IC_50_. For thymol, similar activity was observed by de Medeiros et al., who reported an IC_50_ value of 22.6 *μ*g/mL. IC_50_ values ranging from 9.8 to 65.2 *μ*g/mL were found for other species of *Leishmania* [56, 188, 189]. Both eugenol (**2**) and thymol (**1**) compounds showed low cytotoxicity at concentrations of 50 and 100 *μ*g/mL, where the percentage of viability on L929 fibroblasts ranged from 46.1 to 64.5%.

### 1.4. Essentials Oils Active against Arboviruses, Especially Dengue

Arboviruses (arthopod-borne viruses) are viral diseases transmitted to humans by arthropods (insect and arachnid). Among the vectors, one that stands out is the mosquito *Aedes aegypti*, which every year transmits diseases to more than 700 million people worldwide. According to the World Health Organization, it causes more death than any other arboviral disease, killing about 2.5% of all affected children [201].

*Aedys aegypti* is a carrier of various diseases, including dengue fever, dengue haemorrhagic fever, chikungunya, yellow fever, and even Zika virus [202]. Yellow fever, dengue, and Zika virus have their greatest focus in tropical and subtropical areas [203], while dengue haemorrhagic fever has a greater number of reports in Asia, Thailand, and Indonesia [202].

In recent decades, the spread of dengue has increased worldwide, and in 2012 it was considered the most important viral disease in the world, with more than 100 countries and more than 2.5 billion people (about 40% of the world population) at risk of infection by some mosquito-borne disease. Of this total population, approximately 50-100 million are infected annually, of which 500 thousand cases are considered of greater severity [204, 205]

The mosquito transmits arboviruses through the blood meals of the female. The life cycle consists of egg, larva, pulp, and adult. Eggs are deposited on the walls of any reservoirs containing water, and the ideal temperature range for hatching is 28-33°C; larval and pupal stages take place in the water. The transition phase from larval to pupa lasts 24 hours, and the pupal phase lasts 48 hours until the adult mosquito appears. When adults appear, it takes a few seconds for the stiffening of chitin that allows them to fly [206].

Treatment of dengue, chikungunya, and Zika virus are symptomatic, and vaccines are not yet available. Currently, the only effective way to control these diseases is by breaking the disease transmission cycle, controlling the mosquito population through the use of biological and/or synthetic insecticides and repellents.

In several public health programs, various larvicides and insecticides currently used are effective in eliminating mosquitoes and larvae, including the organophosphates temephos [204] and pyrethroids [207]. However, their continuous use has allowed some mosquitoes to become resistant [204], and the insecticides are not selective, causing damage to other organisms, including humans [205, 208–210]. Temephos, for example, was monitored in 16 countries between 1960 and 2000. It was observed that the agent was active from 48 to 72 hours in water, and it interfered with the viability of other living organisms (in addition to *A. aegypti*), causing serious environmental impact [206].

Currently, in Brazil temephos 1% and Bti (an efficient bioinsecticide) are both used. Bti does not present the same environmental impact as does temephos; however, its high cost makes its general use impossible [206].

In this scenario, it is necessary to identify new larvicides with various modes of action to reduce the development of mechanisms of resistance to pesticides, thereby bolstering public health system efforts [204].

The use of biological agents such as EOs is an alternative to synthetic insecticides, with good selectivity to the target and low toxicity to other organisms and the environment [206, 211, 212].

The use of biological agents has been gaining great prominence, reaching the point of a single review article compiling the larvicidal activity of 361 EOs from 269 plant species. Among these, more than 60% were considered active, with LCs < 100 mg/L [204].

Knowing that it is easier to interfere with the proliferation of *Aedes aegypti* in the larval phase than in the adult phase [208], several EOs are being studied and have already shown to have relevant larvicidal activity, with advantages over synthetic insecticides. Among these advantages, the following stand out: lower risks of allergy, less irritability to the skin, less encephalopathy in children [202], more pleasant odors [201], less toxicity to humans, animals, plant life and other organisms [203], and biodegradable properties [206]. In these terms, EOs are superior for the control of arboviruses [204].

In this context, several authors reported activity of EOs and their phytoconstituents in the control of *Aedes aegypti* adults and larvae. A summary of these studies is presented in Table 3.

Most of the studies, while identifying the constituents of the EO, restricted the experiments of biological activity to the oil itself, with few studies evaluating the activity of each individual substance. In some cases, only the activity of the compounds with the highest concentration was evaluated. This is primarily due to the high cost of these compounds, when they are available for purchase [208].

The monoterpenes were the most tested compounds, especially oxygenated monoterpenes, corresponding to 38% (*n* = 49) of the compounds shown in Table 3. The monoterpene hydrocarbon class presented the best activity. This group included 3-carene (**119**) (LC_50_ = 10.7 and 25.3 *μ*g/mL), *α*-terpinene (**182**) (LC_50_ = 14.7 *μ*g/mL), and limonene (**8**) (LC_50_ = 18.1 *μ*g/mL), among others.

Knowing the isolated constituents of the EOs and their activity allowed the execution of chemical analysis and quantitative structure-activity relationship (QSAR), which identifies characteristics related to the structure of the compound that describe the biological activity, making possible the design of new promising compounds.

One study conducted a consensus principal component analysis (CPCA) and principal component analysis (PCA), identifying characteristics related to hydrophobicity (LogS, DRY, and H_2_O) of thymol (**1**) and carvacrol (**10**) derivatives that were important to explain the activities of the compounds against larva of *Aedes aegypti* [227]. Another study performed CPCA and PCA in a series of 55 substances isolated from EOs, obtaining similar results. They highlighted the importance of hydrophobicity (DRY and H_2_O) for the activity of these compounds against the larva of *Aedes aegypti*; however, steric characteristics were also important to explain this activity, indicating that these characteristics were crucial to the activity of these substances, based on the evaluated compounds [228].

## 2. Conclusions

In this work, a large number of papers that dealt with the use of EOs, their fractions, and/or their chemical constituents as treatment options for some neglected diseases were reviewed. Despite the full knowledge that mankind makes use of plants for medicinal purposes from its origin, the absence of new effective drugs, with fewer side effects that can be used against resistant strains of the parasites, promotes a strong demand for natural products, especially for the poorest and most affected populations that are continuously under risk of these diseases. Several EOs and chemical constituents were identified, which have biological activities equipotential to those of the few commercial drugs available, with good margins of safety, suggesting that popular use cannot be neglected.

As examples for isolated active compounds against *T. cruzi*, the isolated compounds thymol (**1**) and linalool (**3**) showed similar activity when compared with benznidazole and quantified activity in nanomolar concentrations, respectively. In addition, semisynthetic aphidicolin (**28**) derivatives (diterpenes) exhibited high potency and selectivity against the same parasite.

For EOs with anti-*T. brucei* activity, the isolated compounds rotundifolone (**88**) and (−)-perillyl aldehyde (**92**) were slightly less active than the reference drug suramin, and carvacrol (**10**), ascaridole (**37**), and caryophyllene oxides (**38**) showed strong potential to growth inhibition of the parasite, with activity comparable to the same standard drug. In addition, some EOs and isolated compounds demonstrated promising antitrypanosomal activity and high selectivity index values, specially the compounds carlina oxide (**139**) (isolated from *Carlina acaulis* roots), linalool (**3**) (which exhibited potential activity against different trypanosome species), and *C. nardus* EO (rich in oxygenated monoterpenes), which demonstrated IC_50_ values ranged from 0.31 to 2.5 *μ*g/mL and high selectivity index (>40).

For antileishmanial activity, several EOs or isolated compounds were active against the parasite (IC_50_ ≤ 10 *μ*g/mL). Some of them promoted morphological alterations in the parasite, but few showed a great ratio of efficacy to toxicity. Linalool-rich EO (from *Croton cajucara*) and purified linalool (**3**) showed potent antileishmanial activity at nanomolar concentration, promoting morphological changes in the parasite, as well as decreased parasite oxygen consumption and enhances the activities of the protein kinases and increases in nitric oxide levels in infected macrophages, confirming some of the mechanisms associated with leishmanicidal activity. Another isolated compound identified as *α*-bisabolol (**165**) (main constituent of EO from *Vanillosmopsis arborea*) efficiently inhibited the growth of promastigotes and amastigotes forms of the same parasite, inducing several ultrastructural alterations and severe cell damage in the parasite, where the very similar percentage of inhibition (compared with pentamidine) and very low toxicity *in vivo* supported their therapeutic use to treat leishmaniasis.

Other EOs rich in spathulenol (**108**) and caryophyllene oxide (**38**) (from *Piper angustifolium*) and citral (**63**) (from *Cymbopogon citratus*) showed potential activity against promastigote and amastigote forms of the parasite and good SI values.

For control of *Aedes aegypti* adults and larvae, some monoterpene hydrocarbon EOs, specially 3-carene (**119**), *α*-terpinene (**182**), and limonene (**8**), exhibited the best activity.

In this sense, knowledge of the chemical structures of these phytochemicals may allow them to serve as scaffolds for rational drug design, suggesting chemical modifications to increase activity, bioavailability, and toxicity, among other characteristics, allowing the design of new and more active compounds.

## Figures and Tables

**Table 1:** Active EOs against *Leishmania* species.

Plant family	Plant specie	Plant origin	Part of plant	Major constituents (%)	LC_50_/*Leishmania* specie (evolutive form)	SI	Ref.
Rutaceae	*Ruta chalepensis*	Tunisia/Sned region	Aerial parts	2-Undecanone (**162**) (84.28%)	8.16 *μ*g.mL^−1^/*L. major* (promastigote) 1.13 *μ*g.mL^−1^/*L. infantum* (promastigote)	1.57 for *L. major* and 2.7 for *L. infantum* ^a^	[143]

Umbelliferae	*Pituranthos tortuosus*	Tunisia/Sned region	Aerial parts	nd	0.64 *μ*g.mL^−1^/*L. major* (promastigote) 0.66 *μ*g.mL^−1^/*L. infantum* (promastigote)	0.78 for *L. major* and 0.76 for *L. infantum* ^a^	[143]

Cyperaceae	*Cyperus rotundus*	Tunisia/Sned region	Aerial parts	nd	1.40 *μ*g.mL^−1^/*L. major* (promastigote) 0.55 *μ*g.mL^−1^/*L. infantum* (promastigote)	0.09 for *L. major* and 0.23 for *L. infantum* ^a^	[143]

*Cupressaceae*	*Tetraclinis articulata*	Tunisia/Sned region	Aerial parts	nd	>8 *μ*g.mL^−1^/*L. major* (promastigote) 3.31 *μ*g.mL^−1^/*L. infantum* (promastigote)	0.25 for *L. major* and 0.6 for *L. infantum* ^a^	[143]

*Pinaceae*	*Pinus halepensis*	Tunisia/Sned region	Aerial parts	nd	2.23 *μ*g.mL^−1^/*L. major* (promastigote) 1.92 *μ*g.mL^−1^/*L. infantum* (promastigote)	0.61 for *L. major* and 0.94 for *L. infantum* ^a^	[143]

Fabaceae	*Copaifera reticulata*	Brazil/Pará State	Trunks	*β*-Caryophyllene (**22**) (40.9%)	5.0 *μ*g.mL^−1^/*L. amazonensis* (promastigote) 15.0 *μ*g.mL^−1^/*L. amazonensis* (axenic amastigote) 20.0 *μ*g.mL^−1^/*L. amazonensis* (intracellular amastigote)	8.0 for promastigote and 2.5 for axenic amastigote	[153]
	*Copaifera multijuga*	Brazil/Amazonas State	Trunks	*β*-Caryophyllene (**22**) (57.5%) Copalic acid (**164**) (6.2%)	10.0 *μ*g.mL^−1^/*L. amazonensis* (promastigote)	nd	[153]
	*Vouacapoua americana*	French Guiana	Wood	nd	7.2 *μ*g/mL/*L. amazonensis* (axenic amastigote)	5.0	[190]
	*Copaifera* ssp. (C1) commercial oil	Brazil/Acre State	nd	*trans*-*β*-Caryophyllene (**22**) (44.23%) *α*-Humulene (**136**) (7.29) Caryophyllene oxide (**38**) (10.17)	2.9 *μ*g.mL^−1^/*L. amazonensis* (amastigote)	29.3	[157]
	*Copaifera* ssp. (C4) commercial oil	Brazil/Pará State	nd	*trans*-*β*-Caryophyllene (**22**) (36.46%) Copalic acid (**164**) (7.62%) Germacrene D (**24**) (4.56%)	2.3 *μ*g.mL^−1^/*L. amazonensis* (amastigote)	40.1	[157]

Lamiaceae	*Satureja punctata*	Inchini/Addis Abada	Leaves	Geranial (**6**) (27.62%) Neral (**7**) (21.72%) *α*-Bisabolol (**165**) (13.62%)	156.5 *μ*g.mL^−1^/*L. donovani* (promastigote) 312.5 *μ*g.mL^−1^/*L. aethiopica* (promastigote) 8.70 *μ*g.mL^−1^/*L. donovani* (axenic amastigote) 4.06 *μ*g.mL^−1^/*L. aethiopica* (axenic amastigote)	0.001 for *L. donovani* and 0.05 for *L. aethiopica*	[159]
	*Thymus hirtus* sp. *Algeriensis*	Tunisia/Sned region	Aerial parts	Linalool (**3**) (17.62%) Camphor (**14**) (13.82%) Terpinen-4-ol (**103**) (6.80%)	0.43 *μ*g.mL^−1^/*L. major* (promastigote) 0.25 *μ*g.mL^−1^/*L. infantum* (promastigote)	0.19 for *L. major* and 1.34 for *L. infantum* ^a^	[143]
	*Rosmarinus officinalis*	Tunisia/Sned region	Aerial parts	nd	>8 *μ*g.mL^−1^/*L. major* (promastigote) 3.05 *μ*g.mL^−1^/*L. infantum* (promastigote)	0.09 for *L. major* and 0.23 for *L. infantum* ^a^	[143]
	*Teucrium alopecurus*	Tunisia/Sned region	Aerial parts	nd	1.14 *μ*g.mL^−1^/*L. major* (promastigote) 0.30 *μ*g.mL^−1^/*L. infantum* (promastigote)	0.05 for *L. major* and 0.21 for *L. infantum* ^a^	[143]
	*Lavandula multifida*	Tunisia/Sned region	Aerial parts	nd	>8 *μ*g.mL^−1^/*L. major* (promastigote) >8 *μ*g.mL^−1^/*L. infantum* (promastigote)	nd	[143]
	*Lavandula stoechas*	Morocco/Province of Quezzane	Aerial parts	Fenchone (**166**) (31.81%) Camphor (**14**) (29.60%) Terpineol (**111**) (13.1%)	0.9 *μ*g/mL/*L. major* (promastigote) 7 *μ*g/mL/*L. infantum* (promastigote)	nd	[171]
	*Origanum compactum Benth*	Morocco/Province of Quezzane	Aerial parts (vegetative, flowering and post-flowering)	*p*-Cymene (**11**) (17.81–19.24%) Thymol (**1**) (15.32-38.01%) Carvacrol (**10**) (6.39–43.58%)	0.13–0.26 *μ*g.mL^−1^ */L. major* (promastigote) 0.02–0.12 *μ*g.mL^−1^ */L. infantum* (promastigote) 0.22–0.72 *μ*g.mL^−1^ */L. tropica* (promastigote)	nd	[173]
	*Mentha pulegium*	Morocco/Province of Quezzane	Aerial parts	Pulegone (**128**) (40.98%) Menthone (**132**) (21.164%)	1.3 *μ*g.mL^−1^/*L. major* (promastigote) 2 *μ*g.mL^−1^ */L. infantum* (promastigote) 2.2 *μ*g.mL^−1^ */L. tropica* (promastigote)	nd	[172]
	*Rosmarinus officinalis*	Morocco/Province of Quezzane	Aerial parts	1,8-Cineole (**124**) (23.673%) Camphor (**14**) (18.743%) Borneol (**112**) (15.46%)	2.6 *μ*g.mL^−1^/*L. major* (promastigote) 1.2 *μ*g.mL^−1^ */L. infantum* (promastigote) 3.5 *μ*g.mL^−1^ */L. tropica* (promastigote)	nd	[172]
	*Tetradenia riparia*	Brazil/Paraná State	Leaves	**Summer** Fenchone (**166**) (5.54%) *α*-Cadinol (**53**) (16.91%) 14-Hydroxy-9-epi-caryophyllene (**167**) (15.28%);	**Summer** 15.67 ng.mL^−1^/*L. amazonensis* (promastigote)	**Summer** 94.19	[191]
				**Winter** *α*-Cadinol (**53**) (14.82%) 14-Hydroxy-9-epi-caryophyllene (**167**) (10.23%) Dehydroroyleanone (**168**) (20.47%)	**Winter** 13.31 ng.mL^−1^/*L. amazonensis* (promastigote)	**Winter** 76.80	
				**Autumn** *α*-Cadinol (**53**) (17.16%) 14-Hydroxy-9-epi-caryophyllene (**167**) (13.10%) Dehydroroyleanone (**168**) (16.50%).	**Autumn** 15.66 ng.mL^−1^/*L. amazonensis* (promastigote)	**Autumn** 25.01	
				**Spring** *α*-Cadinol (**53**) (13.81%) 14-Hydroxy-9-epi-caryophyllene (**167**) (12.70%) 6,7-Dehydroroyleanone (**168**) (12.51%)	**Spring** 15.47 ng.mL^−1^/*L. amazonensis* (promastigote)	**Spring** 67.51	
	*Tetradenia riparia*	Brazil/Paraná State	Leaves	nd	0.03 *μ*g.mL^−1^/*L. amazonensis* (promastigote)	5.67	[192]
	*Tetradenia riparia*	Brazil/Paraná State	Leaves	nd	30.0 *μ*g.mL^−1^/*L. amazonensis* (intracellular amastigote)	nd	[175]
	*Teucrium polium*	Northern Tunisia	Aerial parts	Carvacrol (**10**) (56.06%) *β* -Caryophyllene (**22**) (7.68%) *α*-Pinene (**118**) (5.02%)	0.09 *μ*g.mL^−1^/*L. infantum* (Promastigotes) 0.15 *μ*g.mL^−1^/*L. major* (promastigotes)	40.44 for *L. infantum* and 24.26 for *L. major*	[174]
	*Salvia officinalis*	Northern Tunisia	Aerial parts	nd	2.67 *μ*g.mL^−1^/*L. infantum* (Promastigote) 3.4 *μ*g.mL^−1^/*L. major* (promastigote)	7.54 for *L. infantum* and 5.92 for *L. major*	[174]
	*Thymus hirtus*	Northern Tunisia	Aerial parts	*α*-Pinene (**118**) (16.93%)	5.90 *μ*g.mL^−1^/*L. infantum* (Promastigote) 8.8 *μ*g.mL^−1^/*L. major* (promastigote)	24.78 for *L. infantum* and 16.61 for *L. major*	[174]

Asteraceae	*Artemisia absinthium* L.	Inchini/Addis Abada	Leaves	Camphor (**14**) (27.40%) Davanone (**169**) (16.43%) Ethyl(*E*)-cinnamate (**170**) (5.81%)	0.15 *μ*g.mL^−1^/*L. donovani* (promastigote) 0.15 *μ*g.mL^−1^/*L. aethiopica* (promastigote) 42.0 *μ*g.mL^−1^/*L. donovani* (axenic amastigote) 7.94 *μ*g.mL^−1^/*L. aethiopica* (axenic amastigote)	3.6 for *L. donovani* and 19.2 for *L. aethiopica*	[158]
	*Echinops kebericho* Mesfin	Inchini/Addis Abada	Tubers	Dehydrocostus lactone (**171**) (41.83%) *β*-Phellandrene (**106**) (10.84%) Germacrene B (**151**) (5.38%)	0.07 *μ*g.mL^−1^/*L. donovani* (promastigote) 0.00097 *μ*g.mL^−1^/*L. aethiopica* (promastigote) 0.50 *μ*g.mL^−1^/*L. donovani* (axenic amastigote) 0.24 *μ*g.mL^−1^/*L. aethiopica* (axenic amastigote)	0.8 for *L. donovani* and 1.7 for *L. aethiopica*	[158]
	*Achillea millefolium*	Brazil/Paraná State	Leaves and flowers	nd	7.8 *μ*g.mL^−1^/*L. amazonensis* (promastigote) 6.5 *μ*g.mL^−1^/*L. amazonensis* (intracellular amastigote)	9.2 for promastigote and 11.0 for intracellular amastigote	[161]
	*Matricaria chamomilla*	USA/Clackamas	Flowers	nd	10.30 *μ*g.mL^−1^/*L. braziliensis* (intracellular amastigote) 2.87 *μ*g.mL^−1^/*L. panamensis* (intracellular amastigote) 230 *μ*g.mL^−1^/*L. braziliensis* (axenic amastigote)	2.93 for *L. braziliensis* intracellular amastigote, 10.52 for *L. panamensis* intracellular amastigote, and 0.14 for *L. braziliensis* axenic amastigote	[193]
	*Pulicaria gnaphalodes*	Iran/Tabas region	Aerial parts	1,8-Cineole (**124**) (9.45%) *α*-Pinene (**118**) (3.81%) *α*-Terpineol (**111**) (3.63%)	0.27 *μ*g.mL^−1^/*L. major* (promastigote)	nd	[194]
	*Vanillosmopsis arborea Baker*	Brazil/Ceará State	Stems	*α*-Bisabolol (**165**) (97.9%) *o*-Methyl eugenol (**172**) (1.6%) Bisabolol oxide (**173**) (0.5%)	7.35 *μ*g.mL^−1^/*L. amazonensis* (promastigote) 12.58 *μ*g.mL^−1^/*L. amazonensis* (intracellular amastigote)	11.52	[183]
	*Artemisia herba alba*	Northern Tunisia	Aerial parts	nd	1.22 *μ*g.mL^−1^/*L. infantum* (promastigote) 2.78 *μ*g.mL^−1^/*L. major* (promastigote)	7.21 for *L. infantum* and 3.16 for *L. major*	[174]
	*Artemisia campestris*			*α*-Pinene (**118**) (24.98%)	3.24 *μ*g.mL^−1^/*L. infantum* (promastigote) 4.59 *μ*g.mL^−1^/*L. major* (promastigote)	24.87 for *L. infantum* and 17.55 for *L. major*	[174]
	*Mikania micrantha*	French Guiana		nd	6.8 *μ*g.mL^−1^/*L. amazonensis* (axenic amastigote)	7.0	[190]
	*Artemisia annua*	Jamia Hamdard	Leaves	Camphor (**14**) (52.06%) *β*-Caryophyllene (**22**) (10.95%) 1,8-Cineole (**124**) (5.57%)	14.63 *μ*g.mL^−1^/*L. donovani* (promastigote) 7.3 *μ*g.mL^−1^/*L. donovani* (Intracellular amastigote)	CC_50_ > 200 *μ*g.mL^−1^	[163]

Myrtaceae	*Eugenia uniflora* L.	Brazil/São Luís State	Leaves	Curzerene (**146**) (47.3%) *γ*-Elemene (**152**) (14.25%) *trans*-*β*-Elemenone (**174**) (10.4%)	3.04 *μ*g.mL^−1^/*L. amazonensis* (promastigote) 1.92 *μ*g.mL^−1^/*L. amazonensis* (amastigote)	14.9 for promastigote and 23.9 for amastigote	[163]
	*Myrtus communis*	nd	Aerial parts	*α*-Pinene (**118**) (52.52%)	4.58 *μ*g.mL^−1^/*L. infantum* (promastigote) 6.28 *μ*g/mL/*L. major* (promastigote)	27.86 for *L. infantum* and 20.31 for *L. major*	[174]
	*Eucalyptus globulus*	Tunisia/Sned region	Aerial parts	nd	0.98 *μ*g.mL^−1^/*L. major* (promastigote) 0.68 *μ*g.mL^−1^/*L. infantum* (promastigote)	0.27 for *L. major* and 0.38 for *L. infantum* ^a^	[143]
	*Myrtus communis*	Kerman province	Leaves	*α*-Pinene (**118**) (24.7%) 1,8-Cineole (**124**) (19.6%) Linalool (**3**) (12.6%)	8.4 *μ*g/mL/*L. tropica* (promastigote) 11.6 *μ*g/mL/*L. tropica* (amastigote)	11.7	[195]

Euphorbiaceae	*Croton cajucara*	Brazil/Amazonas State	Leaves	Linalool (**3**)	8.3 ng.mL^−1^/*L. amazonensis* (promastigote) 15.5 ng.mL^−1^/*L. amazonensis* (amastigote)	nd	[162]
	*Croton pedicellatus*	Tolima	Leaves	Borneol (**112**) *γ*-Terpinene (**64**) Germacrene D (**24**) *trans*-*β*-Caryophyllene (**22**)	46.68 *μ*g.mL^−1^/*L. panamensis* (amastigote) 7.14 *μ*g.mL^−1^/*L. panamensis* (promastigote) 19.77 *μ*g.mL^−1^/*L. braziliensis* (amastigote) 19.65 *μ*g.mL^−1^/*L. braziliensis* (promastigote)	0.83 for *L. panamensis* (amastigotes), 1.30 for *L. panamensis* (promastigotes), 0.47 for *L. braziliensis* (amastigotes), and 0.47 for *L. braziliensis* (promastigotes)	[196]

Poaceae	*Cymbopogon citratus*	Brazil/Paraná State	Leaves	Citral (**63**) Geranial (**6**) (42.2%) Neral (**7**) (36.3%)	1.7 *μ*g.mL^−1^/*L. amazonensis* (promastigote) 3.2 *μ*g.mL^−1^/*L. amazonensis* (amastigote)	14.7 for promastigote and 7.8 for amastigote	[167]
	*Cymbopogon citratus*	French Guiana	Leaves	nd	5.3 *μ*g.mL^−1^/*L. amazonensis* (axenic amastigote)	5.0	[188]

Piperaceae	*Piper angustifolium Lam.*	Brazil/Mato Grosso do Sul State	Leaves	Spathulenol (**108**) (23.78%) Caryophyllene oxide (**38**) (13.06%)	1.43 *μ*g.mL^−1^/*L. infantum* (intracellular amastigote)	22.15	[170]
	*Piper hispidum (Piperaceae)*	French Guiana	Leaves	Curzerene (**146**) (15.7%), Germacrene B (**151**) (10.9%) *α*-Selinene (**175**) (10.5%)	3.4 *μ*g.mL^−1^/*L. amazonensis* (axenic amastigote) 4.7 *μ*g.mL^−1^/*L. amazonensis* (intracellular amastigote)	11.0 for axenic amastigote and 8.0 for intracellular amastigotes-	[188]

Annonaceae	*Annona foetida*	Brazil/Amazonas State	Leaves	Bicyclogermacrene (**23**) (35.12%) (*E*)-Caryophyllene (**22**) (14.19%) *α*-Copaene (**113**) (8.19%)	16.2 *μ*g.mL^−1^/*L. amazonensis* (promastigote) 9.9 *μ*g.mL^−1^/*L. braziliensis* (promastigote) 27.2 *μ*g.mL^−1^/*L. chagasi* (promastigote) 4.1 *μ*g.mL^−1^/*L. guyanensis* (promastigote)	nd	[197]
	*Xylopia discreta*	Colombia/Municipality of Barrancabermeja	Leaves	*α*- and *β*-Pyrene (**176**) Camphene (**15**) *β*-Myrcene (**61**) 1,8-Cineol (**128**)	EC_50_ = 6.35^b^ and 6.25^c^ *μ*g.mL^−1^/*L. panamensis* (promastigote)^b^	11.0^b^ and 25.6^c^	[198]

Verbenaceae	*Lippia citriodora*	Antioquia	Aerial parts	Limonene (**8**) (8.4%) Neral (**7**) (15.0%) Geranial (**6**) (17.5%)	5.2 *μ*g.mL^−1^/*L. chagasi* (promastigote)	nd	[56]
	*Lippia origanoides*	Cauca/Mercaderes	Aerial parts	p-Cymene (**11**) (11.5%) Thymol (**1**) (53.6%)	4.4 *μ*g.mL^−1^/*L. chagasi* (promastigote)	nd	[56]
	*Lantana camara*	Brazil/Minas Gerais State	Leaves	Germacrene-D (**24**) (24.90%) (*E*)-Caryophyllene (**22**) (14.31%) (*E,E*)-Farnesene (**117**) (11.58%)	0.25 *μ*g.mL^−1^/*L. amazonensis* (promastigote) 18 *μ*g.mL^−1^/*L. chagasi* (promastigote)	LC_50_ 10 *μ*g/mL and CC_50_ 4 *μ*g/mL	[145]

Apiaceae	*Ferula communis*	Northern Tunisia	Aerial parts	*β*-Caryophyllene (**22**) (15.22%) Myrcene (**61**) (10.33%) *α*-Eudesmol (**177**) (9.8%)	0.05 *μ*g.mL^−1^/*L. infantum* (promastigote) 0.11 *μ*g.mL^−1^/*L. major* (promastigote)	81.60 for *L. infantum* 37.09 for *L. major*	[174]

Geraniaceae	*Pelargonium graveolens*	Northern Tunisia	Aerial parts	Citronellol (**51**) (24.75%) Geraniol (**6**) (13.99%) *γ*-Eudesmol (**150**) (11.23%)	0.11 *μ*g.mL^−1^/*L. infantum* (promastigote) 0.28 *μ*g.mL^−1^/*L. major* (promastigote)	57.36 for *L. infantum* 22.53 for *L. major*	[174]

Burseraceae	*Protium ovatum*	Brazil/Goiás State	Leaves	Spathulenol (**108**) (17.6%) Caryophyllene oxide (**38**) (16.4%) *β*-Caryophyllene (**22**) (14.0%)	2.28 *μ*g.mL^−1^/*L. amazonensis* (promastigote)	nd	[199]
	*Protium heptaphyllum*	French Guiana	Fruits	nd	3.7 *μ*g.mL^−1^/*L. amazonensis* (axenic amastigote)	19.0	[188]

Scrophulariaceae	*Achetaria guianensis*	French Guiana	Leaves and stems	nd	6.3 *μ*g.mL^−1^/*L. amazonensis* (axenic amastigote)	5.0	[188]

Plantaginaceae	*Otacanthus azureus*	French Guiana	Aerial parts	nd	0.7 *μ*g.mL^−1^/*L. amazonensis* (axenic amastigote) 16.1 *μ*g.mL^−1^/*L. amazonensis* (intracellular amastigote)	51.0 for axenic amastigote and 2.0 for intracellular amastigote	[188]

Chenopodiaceae	*Chenopodium ambrosioides*	Havana City	Aerial parts	Carvacrol (**10**) (62.36%) Ascaridole (**37**) (22.54%)	EC_50_ = 3.7 *μ*g.mL^−1^/*L. amazonensis* (promastigote) EC_50_ = 4.6 *μ*g.mL^−1^/*L. amazonensis* (amastigote)	15.0	[120]
	*Chenopodium ambrosioides*	Havana City	Aerial parts	nd	EC_50_ = 4.45 *μ*g.mL^−1^/*L. donovani* (promastigote) EC_50_ = 5.14 *μ*g.mL^−1^/*L. donovani* (amastigote)	nd	[177]
	*Chenopodium ambrosioides*	Havana City	Aerial parts	nd	3.74 *μ*g.mL^−1^/*L. amazonensis* (promastigote)	nd	[176]

Bixaceae	*Bixa orellana* L.	Havana City	Fruits	Ishwarane (**178**) (18.6%) Geranylgeraniol (**5**) (9.1%) Bicyclogermacrene (**23**) (8.4%)	8.54 *μ*g.mL^−1^/*L. amazonensis* (amastigote)	7.0	[181]

^a^SI calculated as IC_80_/IC_50_. ^b^Murine infection assay (J774 cells infected with *L. panamensis*). ^c^Human infection assay (U937 cells infected with *L. panamensis*). nd: not determined.

**Table 2:** Active and inactive isolated compounds and/or synthesized derivatives from EOs actives against *Leishmania* species.

Classes	Name and/or chemical structure of isolated or synthesized compound	Activity (IC_50_)/*Leishmania* specie (evolutive form)	SI	Ref.
Monoterpene hydrocarbon	3-Carene (**119**)	72.5 *μ*g.mL^−1^/*L. amazonensis* (promastigote)	nd	[187]
	*p*-Cymene (**11**)	>1000 *μ*g.mL^−1^/*L. amazonensis* (promastigote)	nd	[187]
	*α*-Pinene (**118**)	19.7 *μ*g.mL^−1^/*L. amazonensis* (promastigote) 16.1 *μ*g.mL^−1^/*L. amazonensis* (axenic amastigote) 15.6 *μ*g.mL^−1^/*L. amazonensis* (intracellular amastigote)	21.5 for promastigote, 26.4 for axenic amastigote, and 27.2 for intracellular amastigote	[169]

Oxygenated monoterpene	Carvacrol (**10**)	25.4 *μ*g.mL^−1^/*L. amazonensis* (promastigote)	nd	[187]
	Thymol (**1**)	26.8 *μ*g.mL^−1^/*L. amazonensis* (promastigote) 9.8 *μ*g.mL^−1^/*L. chagasi* (promastigote)	nd	[187, 200]
	(−)-Carvone (**13**)	194.7 *μ*g.mL^−1^/*L. amazonensis* (promastigote)	nd	[187]
	(−)-Menthol (**130**)	198.9 *μ*g.mL^−1^/*L. amazonensis* (promastigote)	nd	[187]
	1,8-Cineole (**124**)	568.1 *μ*g.mL^−1^/*L. amazonensis* (promastigote)	nd	[187]
	Linalool (**3**)	550 *μ*g.mL^−1^/*L. infantum chagasi* (axenic amastigote) 276.2 *μ*g.mL^−1^ or 8.3 ng.mL^−1^/*L. amazonensis* (promastigote) 15.5 ng.mL^−1^/*L. amazonensis* (amastigote)	nd	[162] [187] [166]
	Citral (**63**)	8.0 *μ*g.mL^−1^/*L. amazonensis* (promastigotes) 25.0 *μ*g.mL^−1^/*L. amazonensis* (amastigotes)	6.3 for promastigote and 2.0 for amastigote	[167]
	Geraniol (**9**)	3.78 *μ*g.mL^−1^/*L. infantum* (promastigote) 5.57 *μ*g.mL^−1^/*L. major* (promastigote)	21.26 for *L. infantum* 14.43 for *L. major*	[174]
	Camphor (**14**)	5.55 *μ*g.mL^−1^/*L. infantum* (promastigote) 7.90 *μ*g.mL^−1^/*L. major* (promastigote)	4.56 for *L. infantum* 3.20 for *L. major*	[174]
	Carvacrol (**10**)	7.35 *μ*g.mL^−1^/*L. infantum* (promastigote) 9.15 *μ*g.mL^−1^/*L. major* (promastigotes) 2.3 *μ*g.mL^−1^/*L. chagasi* (promastigote)	34.08 for *L. infantum* and 27.37 for *L. major*	[174, 200]
	Ascaridole (**37**)	0.1 *μ*g.mL^−1^/*L. amazonensis* (promastigote) 0.3 *μ*g.mL^−1^/*L. amazonensis* (amastigote)	4.0	[64]

Oxygenated sesquiterpene	(−)-*α*-Bisabolol (**165**)	10.99 *μ*g.mL^−1^/*L. infantum* (promastigote) 4.95 *μ*g.mL^−1^/*L. amazonensis* (promastigote)	9.38 for *L. amazonensis*	[183, 185]
	Caryophyllene Oxide (**38**)	4.9 *μ*g.mL^−1^/*L. amazonensis* (promastigote) 13.6 *μ*g.mL^−1^/*L. amazonensis* (amastigote)	1.0	[64]
	*β*-Caryophyllene (**22**)	1.3 *μ*g.mL^−1^/*L. amazonensis* (amastigote) 1.06 *μ*g.mL^−1^/*L. infantum* (promastigote) 1.33 *μ*g.mL^−1^/*L. major* (promastigote)	48.9 for *L. amazonensis*, 20.82 for *L. infantum*, and 16.59 for *L. major*	[174]

Phenolic compounds	Eugenol (**2**)	220 *μ*g.mL^−1^/*L. infantum* (promastigote) 500 *μ*g.mL^−1^/*L. infantum chagasi* (axenic amastigote) 82.9 *μ*g.mL^−1^/*L. amazonensis* (promastigote)	nd	[166, 187]

Phenylpropanoid	Isoborneol (**179**) 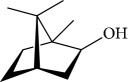	190.2 *μ*g.mL^−1^/*L. amazonensis* (promastigote)	nd	[187]

Diterpene	6,7-Dehydroroyleanone (**167**)	*μ*g.mL^−1^/*L. amazonensis* (promastigote)	0.22	[192]

Others	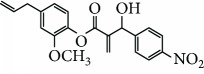	8.75 *μ*g.mL^−1^/*L. amazonensis* (promastigotes)	>45.71	[186]
	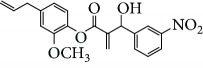	10.49 *μ*g.mL^−1^/*L. amazonensis* (promastigotes)	>38.13	[186]
	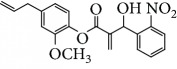	4.71 *μ*g.mL^−1^/*L. amazonensis* (promastigotes)	>84.92	[186]
	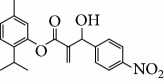	11.40 *μ*g.mL^−1^/*L. amazonensis* (promastigotes)	nd	[186]
	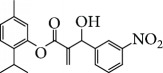	10.56 *μ*g.mL^−1^/*L. amazonensis* (promastigotes)	nd	[186]
	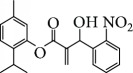	5.91 *μ*g.mL^−1^/*L. amazonensis* (promastigotes)	nd	[186]
	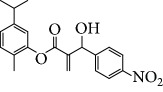	18.08 *μ*g.mL^−1^/*L. amazonensis* (promastigotes)	nd	[186]
	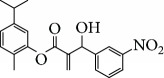	22.30 *μ*g.mL^−1^/*L. amazonensis* (promastigotes)	nd	[186]
	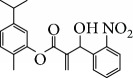	13.60 *μ*g.mL^−1^/*L. amazonensis* (promastigotes)	nd	[186]
	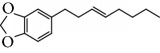 5-[(3*E*)-Oct-3-en-1-il]-1,3-benzodioxole	82.5 *μ*g.mL^−1^/*L. infantum* (promastigotes)	2.4	[186]

nd: not determined.

**Table 3:** Isolated compounds from essential oils and/or synthesized compounds derivated from essential oils actives against *Aedes aegypti*.

Classes	Chemical Structure of isolated or synthesized compound	LC_50_	Reference
Monoterpene hydrocarbons	3-Carene (**119**)	10.7 *μ*g/mL	[213]
		150 ppm	[214]
		25.3 *μ*g/mL	[215]
	*α*-Terpinene (**183**) 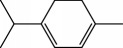	14.7 *μ*g/mL	[213]
		14.7 *μ*g/mL	[216]
		28.1 *μ*g/mL	[215]
	*γ*-Terpinene (**64**)	37.2 *μ*g/mL	[213]
		30.7 *μ*g/mL	[216]
		26.8 *μ*g/mL	[215]
		56 ppm	[214]
	*p*-Cymene (**11**)	43.3 *μ*g/mL	[213]
		51 ppm	[217]
		23.3 ppm	[218]
		19.2 *μ*g/mL	[216]
		37.1 *μ*g/mL	[215]
	*β*-Pinene (**39**)	183.8 *μ*g/mL	[213]
	*R*-Limonene (**8**)	27 ppm	[214]
		17.8 *μ*L L^−1^	[219]
	*S*-Limonene (**8**)	30 ppm	[214]
		33.9 ppm	[220]
		13 *μ*L L^−1^	[219]
	*RS*-Limonene (**8**)	517 ppm	[214]
		18.1 *μ*g/mL	[216]
	*β*-Myrcene (**61**)	35.8 *μ*g/mL	[215]
		120.3 ppm	[221]
	*α*-Pinene (**118**)	>50 *μ*g/mL	[216]
		79.1 *μ*g/mL	[215]
	Terpinolene (**141**)	28.4 *μ*g/mL	[216]
		32.1 *μ*g/mL	[215]
	*α*-Phellandrene (**143**)	39.3 *μ*g/mL^−1^	[222]
		16.6 *μ*g/mL	[216]
	Camphene (**15**)	>400 *μ*g/mL	[213]
	(−)-Camphene (**15**)	220 ppm	[217]
	(+)-Camphene (**15**)	406 ppm	[217]

Oxygenated monoterpenes	(−)-*α*-Terpineol (**111**)	>400 *μ*g/mL	[213]
		>50 *μ*g/mL	[216]
	(−)-Terpinen-4-ol (**103**)	>400 *μ*g/mL	[213]
		>50 *μ*g/mL	[216]
		>100 *μ*g/mL	[215]
	Thymol (**1**)	81 ppm	[217]
		17.5 ppm	[218]
		13.9 ppm	[223]
	Carvacrol (**10**)	69 ppm	[217]
		20.13 ppm	[223]
	Borneol (**112**)	610 ppm	[217]
	Isoborneol (**178**)	598 ppm	[217]
	Camphor (**14**)	657 ppm	[217]
	*R*-Carvone (**13**)	152 ppm	[217]
		129.1 ppm	[217]
	*S*-Carvone (**13**)	124 ppm	[217]
	*RS*-Carvone (**13**)	118 ppm	[214]
	Isopulegol (**184**) 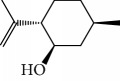	297 ppm	[214]
	Neoisopulegol (**185**) 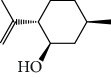	554 ppm	[214]
	Menthol (**130**)	404 ppm	[214]
	Menthone (**132**)	508 ppm	[214]
	1,2-Carvone oxide (**186**) 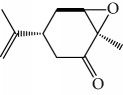	219 ppm	[214]
	(+)-Pulegone (**128**)	188.1 ppm	[220]
	1,4-Cineole (**187**) 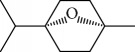	751 ppm	[217]
	1,8-Cineole (**124**)	1419 ppm	[217]
		>100 *μ*g/mL^−1^	[222]
		>50 *μ*g/mL	[216]
	5-Norbornene-2,2-dimethanol (**188**) 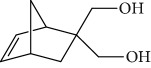	785 ppm	[217]
	5-Norbornene-2-endo,3-endo-dimethanol (**189**) 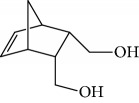	1407 ppm	[217]
	5-Norbornene-2-exo,3-exo-dimethanol (**190**) 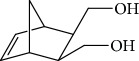	717 ppm	[217]
	5-Norbornene-2-ol (**191**) 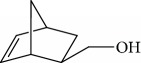	759 ppm	[217]
	(1*R*,2*R*,5*R*)-2-Methyl-5-(1-methylethenyl)-3-oxo-cyclohex-anecarbonitrile 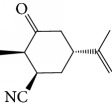	412 ppm	[214]
	Carvacrol methyl ether (**192**) 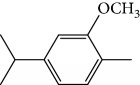	40.6 ppm	[223]
	Umbellulone (**158**)	32.3 ppm	[221]
	Vanillin (**193**) 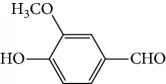	513 ppm	[217]
	*α*-Terpinyl acetate (**194**) 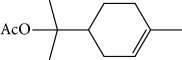	>50 *μ*g/mL	[216]
	Rotundifolone (**88**)	62.5 ppm	[220]
	Pulegone epoxide (**195**) 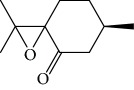	1116.2 ppm	[220]
	(−)-Carvone epoxide (**196**) 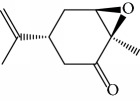	217.5 ppm	[220]
	(+)-Carvone epoxide (**197**) 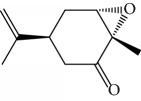	254.6 ppm	[220]
	(+)-Limonene epoxide (**90)**	525.0 ppm	[220]
	(−)-Limonene epoxide (**90)**	522.5 ppm	[220]
	(−)-Perill aldehyde (**92**)	115.8 ppm	[220]
	Perillaldehyde epoxide (**198**) 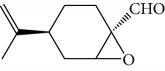	715.1 ppm	[220]
	*trans*-Isopulegone (**199**) 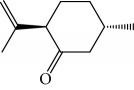	538.8 ppm	[220]
	*trans*-dihydrocarvone (**200**) 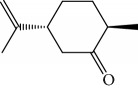	361.3 ppm	[220]
	Hydroxycarvone (**199**) 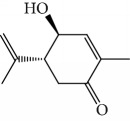	1470.9 ppm	[220]
	Hydroxydihydrocarvone (**200**) 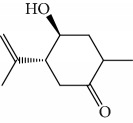	1628.2 ppm	[220]
	Acetoxycarvotanacetone (**201**) 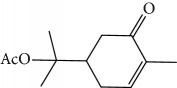	230.7 ppm	[220]

Oxygenated sesquiterpenes	Elemol (**52**)	>100 *μ*g/mL	[215]
	*β*-Eudesmol (**59**)	>50 *μ*g/mL	[224]
		>100 *μ*g/mL	[215]

Diterpenes	16-Kaurene **(202)** 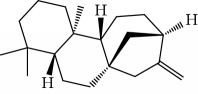	57 *μ*g/mL	[213]
		57.0 *μ*g/ml	[217]

Phenylpropanoids	Estragole (**74**)	46.4 *μ*L L^−1^	[219]
	Eugenol (**2**)	88 ppm	[217]
		93.3 ppm	[224]
		71.9 ppm	[224]
		23.4 ppm	[223]
	1-Acetate-2-methoxy-4-(2-propen-1-yl)-phenol 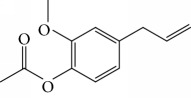	113.3 ppm	[224]
		107.7 ppm	[224]
	1-Propanoate-2-methoxy-4-(2-propen-1-yl)-phenol 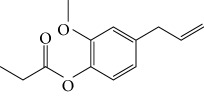	97.2 ppm	[224]
		62.3 ppm	[224]
	1-Benzoate-2-methoxy-4-(2-propen-1-yl)-phenol 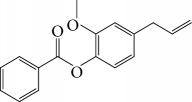	706.8 ppm	[224]
		723.2 ppm	[224]
	1-Benzoate-2-methoxy-4-(3-hydroxypropyl)-phenol 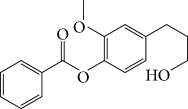	166 ppm	[224]
		151.1 ppm	[224]
	1,2-Dimethoxy-4-(2-propen-1-yl)-benzene 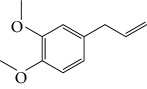	107.3 ppm	[224]
		101.4 ppm	[224]
	1-Ethoxy-2-methoxy-4-(2-propen-1-yl)-benzene 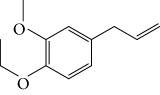	67.2 ppm	[224]
		76.4 ppm	[224]
	1-{[(1,1-Dimethylethyl)dimethylsilyl]oxy}-2-methoxy-4-(2-propen-1-yl)-benzene 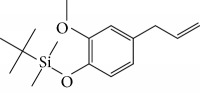	278.9 ppm	[224]
		274.5 ppm	[224]
	4-Hydroxy-3-methoxy-benzenepropanol 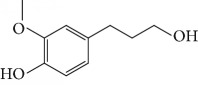	1415.1 ppm	[224]
		1614.9 ppm	[224]
	2-[2-Methoxy-4-(2-propen-1-yl)phenoxy] acetic acid 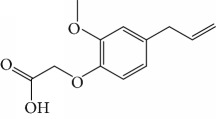	295.9 ppm	[224]
		240.7 ppm	[224]
	Methyleugenol (**171**)	36.5 ppm	[221]
	*trans*-Anethole (**153**)	29.3 *μ*L L^−1^	[219]

Others	Phenol (**203**) 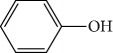	194 ppm	[217]
	1-Dodecanol (**204**) 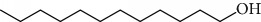	5.2 ppm	[221]
	1-Tridecanol (**205**) 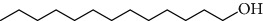	2.1 ppm	[221]
	Allyl *cis*-1-propenyl disulfide 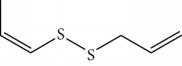	15.35 ppm	[225]
	Allyl methyl disulfide 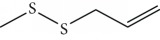	>200 ppm	[225]
		>200 ppm	[26]
	Allyl methyl trisulfide 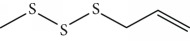	>200 ppm	[225]
	Dimethyl tetrasulfide 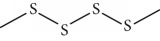	>200 ppm	[226]
	Dimethyl trisulfide 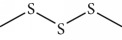	>200 ppm	[225]
		>200 ppm	[226]
	Dipropyl trisulfide 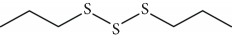	>200 ppm	[225]
		>200 ppm	[226]
	Methlyl propyl trisulfide 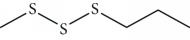	19.38 ppm	[226]
	Catechol (**206**) 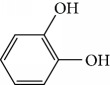	243 ppm	[217]
	Guaiacol (**207**) 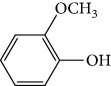	177 ppm	[217]
	Resorcinol (**208**) 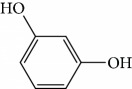	577 ppm	[217]
	Salicylaldehyde (**209**) 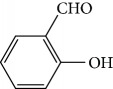	137 ppm	[217]

## Supplementary Materials

Supplementary Materials Figure S1: plant families and number of OEs evaluated between the years 2000 and 2018 against at least one species of Leishmania. Figure S2: plant families and the number of active EOs (IC_50_ ≤ 10 *μ*g/mL) against at least one Leishmania species and parasite form. Table S1: inactive or moderated active EOs against *Leishmania* species.
