# Supplementary material for: Active Essential Oils and Their Components in Use against Neglected Diseases and Arboviruses
Source: Oxid Med Cell Longev. 2019 Feb 7;2019:6587150. doi: 10.1155/2019/6587150 (PMC6387720; doi:10.1155/2019/6587150)
Supplement: Supplementary Materials — Figure S1: plant families and number of OEs evaluated between the years 2000 and 2018 against at least one species of Leishmania. Figure S2: plant families and the number of active EOs (IC50 ≤ 10 μg/mL) against at least one Leishmania species and parasite form. Table S1: inactive or moderated active EOs against Leishmania species. [file 6587150.f1.pdf]

SUPPLEMENTARY MATERIAL

Active Essential Oils and their Components in Use Against Neglected Diseases and Arboviruses

Emanuela Coutinho Luna[1], Isadora Silva Luna[1], Luciana Scotti[2,3], Alex France M. Monteiro[3], Marcus Tullius Scotti[3], Ricardo Olímpio de Moura[1], Rodrigo Santos Aquino de Araújo[1], Kadja Luana Chagas Monteiro[4], Thiago Mendonça de Aquino[5], Frederico Fávaro Ribeiro[1], Francisco Jaime Bezerra Mendonça Junior[1]

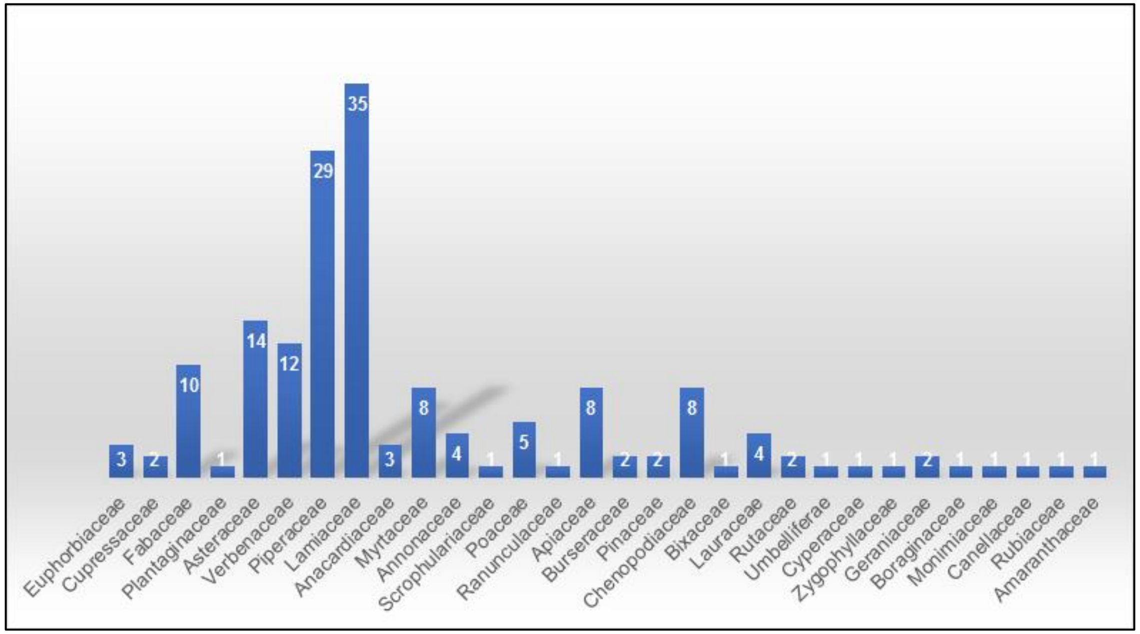

FIGURE S1: Plant families and number of OEs evaluated between the years 2000 and 2018 against at least one species of *Leishmania*.

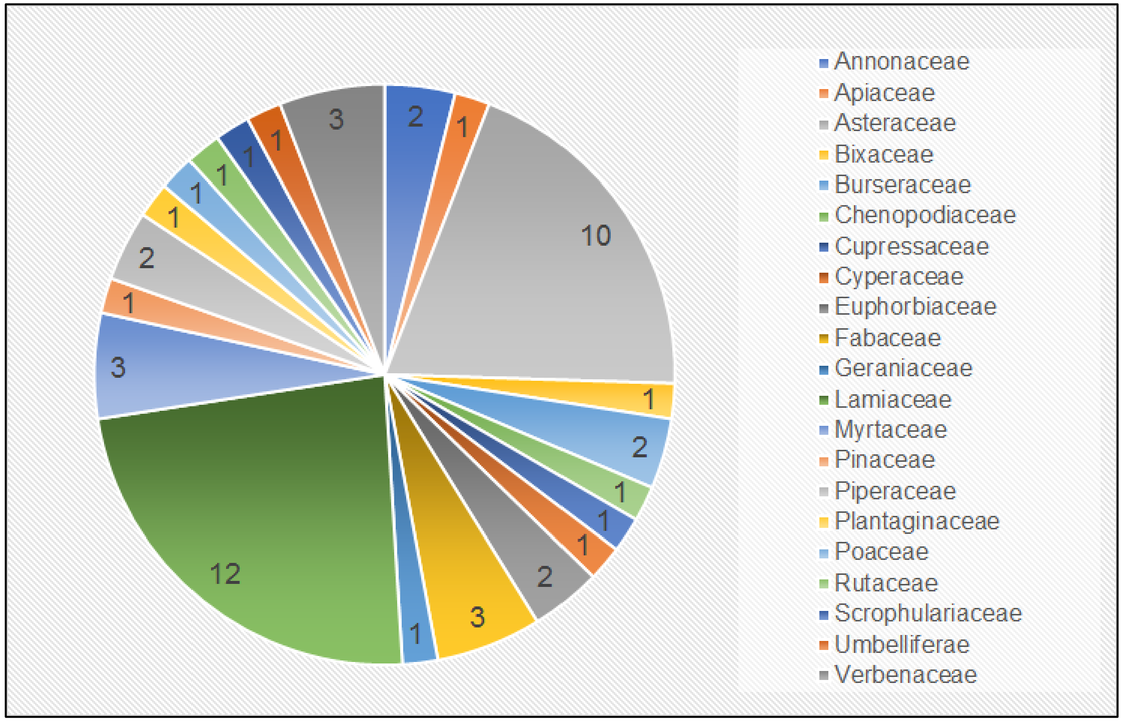

FIGURE S2: Plant families and the number of active EOs (IC_{50} ≤ 10 µg/mL) against at last one *Leishmania* species and parasite form.

Table S1. Inactive or moderated active EOs against *Leishmania* species.

Plant family	Plant specie	Plant origin	Part of plant	Major Constituents (%)	Activity (IC_{50})/*Leishmania* specie (evolutive form)	SI	Ref.
Lauraceae	*Litsea cubeba*	QUINARI Cosmetic and Fragances Inc. (Maringá-Pr, Brazil)	Fruits	nd	nd	nd	[147]
	Cinnamomum camphora		Barks	nd	>500.0 µg/mL/*L. amazonensis* (promastigote)	nd	[147]
	Licaria canella	Brazil/Amazonas State	Leaves	Benzyl benzoate (69.7%)	19.0 µg/mL/*L. amazonensis* (promastigote)	nd	[237]
	Aniba canelilla			1-Nitro-2-phenylethane (88.9%)	40.0 µg/mL/*L. amazonensis* (promastigote)	nd	[237]
	Laurus nobilis	Northen Tunisia	Aerial parts	1,8-Cineole (43.35%)	13.24 µg/mL/*L. infantum* (promastigote)<br>24.36 µg/mL/*L. major* (promastigote)	28.73 for *L. infantum* and 15.61 for *L. major*	[261]
Zingiberaceae	*Elettaria cardamomum*	QUINARI Cosmetic and Fragances Inc. (Maringá-Pr, Brazil)	Seeds	nd	>500.0 µg/mL/*L. amazonensis* (promastigote)	nd	[147]
Zygophyllaceae	*Bulnesia sarmientoi*		Barks	Guaiol (48.29%)<br>2-Undecanone (19.49%)<br>1,8-Cineole (8.71%)	85.56 µg/mL/*L. amazonensis* (promastigote)	1.91	[147]
Apiaceae	*Ferula galbaniflua*		Resin	Methyl 8-pimaren-18-oate (41.82%)<br>β-Pinene (17.34%)<br>Diethyl phthalate (13.09%)	95.70 µg/mL/*L. amazonensis* (promastigote)	3.94	[147]
	Foeniculum officinalis		Leaves	nd	328.28 µg/mL/*L. amazonensis* (promastigote)	1.12	[147]

	Coriandrum sativum	Brazil/Ceará State	Seeds	β-Linalool (73.21%)<br>Camphor (4.25%)<br>α-Pinene (4.20%)	181.0 µg/mL/*L. chagasi* (promastigote)<br>1.51 µg/mL/*L. chagasi* (amastigote)	nd	[239]
Geraniaceae	*Pelargonium graveolens*	QUINARI Cosmetic and Fragances Inc. (Maringá-Pr, Brazil)	Leaves and stems	nd	363.71 µg/mL/*L. amazonensis* (promastigote)	1.01	[147]
Boraginaceae	*Cordia verbenaceae*		Leaves	nd	64.75 µg/mL/*L. amazonensis* (promastigote)	2.01	[147]
Monimiaceae	*Siparuna guianensis*	Brazil/Lavras		Bicyclogermancrene (16.71%)<br>β-Myrcene (13.14%)<br>Germancrene-D (8.68%)	48.55 µg/mL/*L. amazonensis* (promastigote)	1.60	[147]
Canellaceae	*Cinnamodendron dinisii*			α-Pinene (35.41%)<br>β-Pinene (17.81%)<br>Sabinense (12.01%)	54.05 µg/mL/*L. amazonensis* (promastigote)	2.23	[147]
Fabaceae	*Copaifera reticulata*	Brazil/Acre State	Trunks	α-Copaene (25.1%)<br>β-Caryophyllene (13.1%)<br>Copalic acid (7.7%)	22.0 µg/mL/*L. amazonensis* (promastigote)	nd	[156]
	Copaifera martii	Brazil/Pará State		Kovalenic acid (29.0%)<br>β -Bisabolene (10.7%)<br>Kaurenoic acid (7.9%)	14.0 µg/mL/*L. amazonensis* (promastigote)	nd	[156]
	Copaifera cearenses	Brazil/Minas Gerais State		β-Caryophyllene (19.7%)<br>α-Copaene (8.2%)<br>Hardwickiic acid (6.2%)	18.0 µg/mL/*L. amazonensis* (promastigote)	nd	[156]
	Copaifera paupera	Brazil/Acre State		β -Bisabolene (20.2%)<br>α-Zingiberene (19.4%)<br>Kaurenoic acid (13.3%)	11.0 µg/mL/*L. amazonensis* (promastigote)	nd	[156]
	Copaifera langsdorfii	Brazil/São Paulo State		Kaurenoic acid (44.3%)<br>β-Caryophyllene (32.8%)<br>Hardwickiic acid (8.2%)	20.0 µg/mL/*L. amazonensis* (promastigote)	nd	[156]

	Copaifera officinalis	Brazil/Roraima State		Hardwickiic acid (30.7%) Copalic acid (13.9%) β-Caryophyllene (8.5%)	20.0 μg/mL/*L. amazonensis* (promastigote)	nd	[156]
	Copaifera lucens	Brazil/Rio de Janeiro State		Polyalthic acid (69.8%) Copalic acid (11.1%)	20.0 μg/mL/*L. amazonensis* (promastigote)	nd	[156]
	Myroxylon peruiferum (Fabaceae)	QUINARI Cosmetic and Fragances Inc. (Maringá-Pr, Brazil)	Barks	α-copaene (13.41%) Guaiol (9.35%) Safrole (8.35%)	162.25 μg/mL/*L. amazonensis* (promastigote)	0.99	[147]
nd	*Copaifera* ssp. (C2) commercial oil	Brazil/Acre State	nd	Daniellic acid (33.65%) *trans*-*β*-Caryophyllene (12.83%) *β*-Bisabolene (12.65%)	nd	nd	[160]
nd	*Copaifera* ssp. (C3) commercial oil		nd	*α*-Copaene (36.46%) Kaurenoic acid (10.10%) Hardwickiic acid (9.00%)	nd	nd	[160]
nd	*Copaifera* ssp. sesquiterpene-rich fraction		nd	*trans*-*β*-Caryophyllene (42.36%) *α*-Copaene (11.22%) *α*-Bergamotene (9.11%)	nd	nd	[160]
nd	*Copaifera* ssp. Diterpene-rich fraction		nd	Copalic acid (49.87%) Acetoxy-copalic acid (19.56%) Hydroxy-copalic acid (9.39%)	nd	nd	[160]
nd	*Tea tree* commercial oil	nd	nd	nd	ED_{50} = 403.0 μg/mL/*L. major* (promastigote)	0.1	[240]
nd	*Clove* commercial oil	nd	nd	nd	ED_{50} = 58.4 μg/mL/*L. major* (promastigote)	0.6	[240]
nd	*Kanuka* commercial oil	nd	nd	nd	ED_{50} = 26.2 μg/mL/*L. major* (promastigote)	0.6	[240]

nd	*Manuka* commercial oil	nd	nd	nd	ED_{50} = 208.1 µg/mL/*L. major* (promastigote)	1.2	[240]
nd	*Anise* commercial oil	nd	nd	nd	ED_{50} = 286.1 µg/mL/*L. major* (promastigote)	1.1	[240]
nd	*Dwarf pine* commercial oil	nd	nd	nd	ED_{50} = 111.8 µg/mL/*L. major* (promastigote)	0.2	[240]
nd	*Pine* commercial oil	nd	nd	nd	ED_{50} = 123.2 µg/mL/*L. major* (promastigote)	1.2	[240]
nd	*Spruce* commercial oil	nd	nd	nd	ED_{50} = 29.9 µg/mL/*L. major* (promastigote)	8.8	[240]
nd	*Balmmint* commercial oil	nd	nd	nd	ED_{50} = 7.0 µg/mL/*L. major* (promastigote)	3.6	[240]
nd	*Peppermint* commercial oil	nd	nd	nd	ED_{50} = 227.0 µg/mL/*L. major* (promastigote)	0.9	[240]
nd	*Rosemary* commercial oil	nd	nd	nd	ED_{50} = 282.1 µg/mL/*L. major* (promastigote)	0.3	[240]
nd	*Thyme* commercial oil	nd	nd	nd	ED_{50} = 127.4 µg/mL/*L. major* (promastigote)	0.2	[240]
Asteraceae	*Matricaria chamomilla* (Asteraceae)	QUINARI Cosmetic and Fragances Inc. (Maringá-Pr, Brazil)	Flowers	(*E*)-β-Farnesene (52.73%) Bisabolol oxide B (12.09%) (*E*,*E*)-α-Farnesene (10.34%)	60.16 µg/mL/*L. amazonensis* (promastigote)	2.87	[147]
	Artemisia abyssinica	Inchini/Addis Abada	Leaves	Yomogi alcohol (38.47%) Artemisyl acetate (24.88%) Artemisia alcohol (6.70%)	312.5 µg/mL/*L. donovani* (promastigote)	2.7 for *L. donovani* and 28 for *L. aethiopica*	[162]

					76.5 µg/mL/*L. aethiopica* (promastigote)<br>131.00 µg/mL/*L. donovani* (axenic amastigote)<br>12.44 µg/mL/*L. aethiopica* (axenic amastigote)		
	Artemisia abrotanum	Cuba	Aerial parts	nd	MIC = 116.5 mg/mL/*L. amazonensis* (promastigote)	nd	[241]
	Pluchea carolinensis		Leaves	selin-11-en-4α-ol (51.0%)	24.7 µg/mL/*L. amazonensis* (promastigote)<br>6.2 µg/mL/*L. amazonensis* (amastigote)	5	[242]
	Baccharis dracunculifolia DC.	Brazil/ São Paulo	Aerial parts	α-muurolol (4.66%)<br>spathulenol (16.24%)<br>nerolidol (33.51%)	42.0 µg/mL/*L. donovani* (promastigote)	nd	[243]
	Crithmum maritimum	nd		γ-terpinene (48.7%)<br>dillapiole (13.8%)	122.0 µg/mL/*L. infantum* (promastigote)	nd	[244]
	Distichoselinum tenuifolium	nd		Myrcene (84.6%)	295.0 µg/mL/*L. infantum* (promastigote)	nd	[244]
	Eryngium maritimum	nd		Germacrene D (41.1%)	205.0 µg/mL/ *L. infantum* (promastigote)	nd	[244]
	Seseli tortuosum	nd		α-Pinene (27.4%)<br>*β*-pinene (16.0%)<br>Limonene (10.0%)	133.0 µg/mL/*L. infantum* (promastigote)	nd	[244]
Annonaceae	*Cananga odorata*	QUINARI Cosmetic and Fragances Inc. (Maringá-Pr, Brazil)	Flowers	nd	nd	nd	[147]
	Annona coriacea Mart.	Brazil/São Paulo State	Leaves	Bicyclogermacrene (39.8%)<br>γ-Muurolene (7.9%)<br>δ-Cadinene (6.0%)	160.20 µg/mL/*L. amazonensis* (promastigote)<br>261.20 µg/mL/*L. braziliensis* (promastigote)	nd	[245]

					39.93 µg/mL/*L. chagasi* (promastigote) 305.20 µg/mL/*L. major* (promastigote)		
Lamiaceae	*Lavandula officinalis*	QUINARI Cosmetic and Fragances Inc. (Maringá-Pr, Brazil)	Flowers	-	>500.0 µg/mL/*L. amazonensis* (promastigote)	nd	[147]
	Salvia sclarea		Leaves	Linalyl acetate (60.08%) Linalool (28.80%)	325.92 µg/mL/*L. amazonensis* (promastigote)	1.15	[147]
	Melissa officinalis			Geraniale (52.02%) Neral (37.18%)	132.02 µg/mL/*L. amazonensis* (promastigote)	2.25	[147]
	Ocimum basilicum	German	Stems, leaves and flowers	(-)-Linalool (30.7%) (+)-*δ*-Cadinene (3.43%) Eucalyptol (2.66%)	48.3 µg/mL/*L. amazonensis* (promastigote)	nd	[262]
	Ocimum basilicum	Mesten		(-)-Linalool (30.7%) Eugenol (9.68%)	49.6 µg/mL/*L. donovani* (promastigote)	nd	[262]
	Ocimum sanctum	Mississipi		Eugenol (18.0%) Methylchavicol (14.7%) Eucalyptol (5.45%)	37.7 µg/mL/*L. donovani* (promastigote)	nd	[262]
	Basil Bulgaria commercial oil	nd	nd	(-)-Linalool (37.1%) Eucalyptol (2.97%)	40.0 µg/mL/*L. donovani* (promastigote)	nd	[262]
	Basil sweet commercial oil	nd	nd	(-)-Bornyl acetate Methylchavicol (71.3%)	50.0 µg/mL/*L. donovani* (promastigote)	nd	[262]
	Ocimum canum	Brazil/Maranhão State	Leaves	Thymol (42.15%) *p*-Cymene (21.17%) γ-Terpinene (19.81%)	17.4 µg/mL/*L. amazonensis* (promastigote) 13.1 µg/mL/*L. amazonensis* (intracellular amastigote)	18.1 for promastigote and 24.0 for intracellular amastigote	[163]
	Ocimum gratissimum L.	Brazil/ Teresina, Piauí	Leaves	Eugenol (40%) 1,8-Cineole (24%)	75.0 µg/mL/*L. chagasi* (promastigote)	nd	[246]
	Lavandula stoechas	Morocco/ Province of Quezzane	Aerial parts	nd	>10.0 µg/mL/*L. tropica* (promastigote)	nd	[175]

	Rosmarinus officinalis	Northen Tunisia		1,8-Cineole (43.77%)	16.34 µg/mL/*L. infantum* (promastigote)<br>20.92 µg/mL/*L. major* (promastigote)	15.35 for *L. infantum* and 11.99 for *L. major*	[178]
	Lavandula viridis	nd	Leaves	α-Pinene (9.2%)<br>1,8-Cineole (29.7%)<br>Camphor (10.0%)	263.0 µg/mL/*L. infantum* (promastigote)	nd	[244]
	Mentha cervine	nd	Aerial parts	Limonene (5.4%),<br>Isomenthone (10.6%)<br>Pulegone (74.8%)	178.0 µg/mL/*L. infantum* (promastigote)	nd	[244]
	Mentha x piperita	nd		Menthone (9.8%)<br>Menthofuran (10.9%)<br>Menthol (44.0%)	198.0 µg/mL/*L. infantum* (promastigote)	nd	[244]
	Origanum virens	nd		*p*-Cymene (7.4%)<br>γ-Terpinene (7.9%)<br>Carvacrol (68.2%)	196.0 µg/mL/*L. infantum* (promastigote)	nd	[244]
	Rosmarinus officinalis	nd		Myrcene (32.0%)<br>1,8-Cineole (13.7%)<br>Camphor (11.9%)	nd	nd	[244]
	Thymbra capitate	nd		*p*-Cymene (5.5%)<br>Carvacrol (74.6%)	130.0 µg/mL/*L. infantum* (promastigote)	nd	[244]
	Thymus capitelatus	nd		Camphene (6.5%)<br>1,8-Cineole (58.6%)<br>Borneol (10.0%)	37.0 µg/mL/*L. infantum* (promastigote)	nd	[244]
	Thymus mastichina	nd		1,8-Cineole (64.6%)	133.0 µg/mL/*L. infantum* (promastigote)	nd	[244]
	Thymus zygis (chemotype geraniol)	nd		Geraniol (33.1%)<br>Geranyl acetate (44.5%)	162.0 µg/mL/*L. infantum* (promastigote)	nd	[244]
	Thymus zygis (chemotype thymol)	nd		*p*-Cymene (36.6)<br>γ-Terpinene (21.0%)<br>Thymol (15.2%)	293.0 µg/mL/*L. infantum* (promastigote)	nd	[244]

	Thymus capitellatus	Portugal		1,8-Cineole (58.6%)<br>Borneol (10.1%)	37.0 µg/mL/*L. infantum* (promastigote)<br>35.0 µg/mL/*L. tropica* (promastigote)<br>62.0 µg/mL/*L. major* (promastigote)	nd	[247]
Myrtaceae	Syzygium cumini (L.) Skeels	São Luís State/Brazil	Leaves	α-Pinene (31.85%)<br>(*Z*)-β-Ocimene (28.98%)<br>(*E*)-β-Ocimene (11.71%)	60.0 µg/mL/*L. amazonensis* (promastigote)<br>43.9 µg/mL/*L. amazonensis* (axenic amastigote)<br>38.1 µg/mL/*L. amazonensis* (intracelular amastigote)	10.2 for promastigote, 13.9 for axenic amastigote and 16.1 for intracellular amastigote	[173]
	Syzygium cumini (L.) SKEELS	Brazil/ São Luiz (Maranhão)	Leaves	α-Pinene (31.85%)<br>(Z)-β-Ocimene (28.98%)<br>(E)- β-Ocimene (11.71%)	36.0 $mg.L^{-1}$/*L. amazonensis* (promastigote)	nd	[248]
	Eucalyptus globulus	Northen Tunisia	Aerial parts	1,8-cineole (42.31%)	16.28 µg/mL/*L. infantum* (promastigote)<br>18.30 µg/mL/*L. major* (promastigote)	19.04 for *L. infantum* and 16.94 for *L. major*	[178]
	Syzygium aromaticum (L.) (commercial oil)	nd	Buds	Eugenol (85.3%)<br>α-Humulene (6.8%)	220.0 µg/mL/*L. infantum* (promastigote)	nd	[244]
Piperaceae	*Piper rivinoides* Kunth	Brazil/ Paraná (Antonina)	Leaves	α-Humulene (10.0%)<br>Bicyclogermacrene (11.8%)<br>(Z)-α-Bisabolene (10.9%)	10.9 µg/mL/*L. amazonensis* (promastigote)<br>>200.0 µg/mL/*L. amazonensis* (axenic amastigote)	nd	[249]

	Piper mosenii C. DC.	Brazil/ Paraná (Antonina)		(E)-Caryophyllene (8.6%)<br>α-Humulene (11.3%)<br>Caryophyllene oxide (12.1%)	17.4 µg/mL/*L. amazonensis* (promastigote)<br>>200.0 µg/mL/*L. amazonensis* (axenic amastigote)	nd	[249]
	Piper cernuum Vell.	Brazil/ Paraná (Antonina)		α-Pinene (11.4%)<br>β-Elemene (10.1%)<br>Spathuleol (11.5%)	27.1 µg/mL/*L. amazonensis* (promastigote)<br>>200.0 µg/mL/*L. amazonensis* (axenic amastigote)	nd	[249]
	Piper diospyrifolium Kunth	Brazil/ Paraná (Antonina)		(E)-Caryophyllene (7.4%)<br>γ-Gurjunene (6.9%)<br>Selin-11-en-4-α-ol (17.7%)	13.5 µg/mL/*L. amazonensis* (promastigote)<br>76.1 µg/mL/*L. amazonensis* (axenic amastigote)	2.35 for axenic amastigote	[249]
	Piper arboretum Aubl.	Brazil/ Paraná (Antonina)		Spathulenol (7.9%)<br>Caryophyllene oxide (5.9%)<br>1-epi-Cubenol (10.4%)	15.2 µg/mL/*L. amazonensis* (promastigote)<br>>200.0 µg/mL/*L. amazonensis* (axenic amastigote)	nd	[249]
	Piper aduncum L.	Brazil/ Paraná (Cerro Azul)		(E)-β-Ocimene (13.9%)<br>Safrole (6.2%)<br>Bicyclogermacrene (20.9%)	25.9 µg/mL/*L. amazonensis* (promastigote)<br>36.2 µg/mL/ *L. amazonensis* (axenic amastigote)	>5.52 for axenic amastigote	[249]
	Piper gaudichaudianum Kunth	Brazil/ Paraná (Antonina)		δ-3-Carene (5.9%)<br>γ-Elemene (5.4%)<br>δ-Cadinene (45.3%)	93.5 µg/mL/*L. amazonensis* (promastigote)	nd	[249]
	Piper xylosteoides (Kunth) Steud.	Brazil/Paraná State		α-Thujene (7.9%)<br>β-Phellandrene (22.6%)<br>(*E*)-Nerolidol (8.5%)	>100.0 µg/mL/*L. amazonensis* (promastigote)	nd	[249]

	Piper mikanianum (Kunth) Steudel	Brazil/Santa Catarina State		α-Thujene (6.0%)<br>Safrole (72.4%)	>100.0 µg/mL/*L. amazonensis* (promastigote)	nd	[249]
	Piper rivinoides Kunth	Brazil/Paraná State		α-Humulene (10.0%)<br>Bicyclogermacrene (11.8%)<br>(*Z*)-α-Bisabolene (10.9%)	10.9 µg/mL/*L. amazonensis* (promastigote)<br>>200.0 µg/mL/*L. amazonensis* (axenic amastigote)	nd	[249]
	Piper aduncum	Brazil/Minas Gerais State		Nerolidol (25.22%)<br>Linalool (13.42%)	77.9 µg/mL/*L. braziliensis* (promastigote)	1.12 for promatigotes (macrophages) and >6.42 for promastigote (red blood cells)	[250]
	Piper cubeba (commercial oil)	nd	Fruits	Sabinene (19.99%)<br>Eucalyptol(11.87%)<br>4-Terpineol (6.36%)	326.5 µg/mL/*L. amazonensis* (promastigote)	nd	[251]
	Piper auritum	Cuba	Aerial parts	nd	490.7 mg/mL/*L. amazonensis* (promastigote)	nd	[241]
	Piper aduncum			nd	50.8 mg/mL/*L. amazonensis* (promastigote)	nd	[241]
	Piper aduncum var. *ossanum*	Cuba/Bauta	Leaves	Camphene (7.41%)<br>Piperitone (20.07%)<br>Viridiflorol (12.97%)	32.5 µg/mL/*L. infantum* (intracellular amastigote)<br>19.3 µg/mL/*L. amazonensis* (intracellular amastigote)	nd	[252]

	Piper aduncum var. *ossanum*	Cuba/Ceiba		Camphor (9.41%)<br>Piperitone (19.01%)<br>Viridiflorol (18.80%)	32.5 µg/mL/*L. infantum* (intracellular amastigote)<br>>64.0 µg/mL/*L. amazonensis* (intracellular amastigote)	nd	[252]
	Piper auritum Kunth	Colombia/Valle del Cauca	Aerial parts	Safrole (91.3%)<br>Myristicin (4.8%)	>100.0 µg/mL/*L. infantum* (promastigote)<br>>100.0 µg/mL/*L. infantum* (amastigote)	nd	[253]
	Piper bredemeyeri	Colombia/Cesar	Aerial parts	α-Pinene (20.3%)<br>ß-Pinene (32.3%)<br>Limonene (4.1%)	>100.0 µg/mL/*L. infantum* (promastigote)<br>>100.0 µg/mL/*L. infantum* (amastigote)	nd	[253]
	Piper bogotense	Colombia/Nariño		α-Pinene (20.3%)<br>ß-Pinene (32.3%)<br>*trans*-ß-Caryophyllene (6.3%)	>100.0 µg/mL/*L. infantum* (promastigote)<br>>100.0 µg/mL/*L. infantum* (amastigote)	nd	[253]
	Piper marginatum	Colombia/Tolima		α-Phellandrene (11.2%)<br>Limonene (7.6%)<br>*trans*-ß-Caryophyllene (11.1%)	88.70 µg/mL/*L. infantum* (promastigote)<br>>100.0 µg/mL/*L. infantum* (amastigote)	nd	[253]
	Piper cf divaricatum			α-Pinene (11.4%)<br>1,8-Cineole (18.3%)<br>Linalool (15.0%)	73.29 µg/mL/*L. infantum* (promastigote)<br>>100.0 µg/mL/*L. infantum* (amastigote)	nd	[253]
	Piper cf. brachypodon var. *hirsuticaule Yunck*	Colombia/Chocó		ß-Elemene (6.4%)<br>*trans*-ß-Caryophyllene (9.8%)<br>Germacrene D (16.7%)	93.60 µg/mL/*L. infantum* (promastigote)<br>>100.0 µg/mL/*L. infantum* (amastigote)	nd	[253]

	Piper obrutum			Linalool (15.8%)<br>ß-Elemene (7.6%)<br>α-Humulene (6.4%)	35.87 µg/mL/*L. infantum* (promastigote)<br>89.02 µg/mL/*L. infantum* (amastigote)	>1.12	[253]
	Piper septuplinervium			epi-Cubebol (9.0%)<br>δ-Cadinene (10.9%)<br>Viridiflorol (7.9%)	30.05 µg/mL/*L. infantum* (promastigote)<br>64.80 µg/mL/*L. infantum* (amastigote)	1.54	[253]
	Piper lanceifolium			*trans*-ß-Caryophyllene (11.6%)<br>Germacrene D (10.7%)<br>ß-Selinene (7.8%)	37.81 µg/mL/*L. infantum* (promastigote)<br>>100.0 µg/mL/*L. infantum* (amastigote)	nd	[253]
	Piper brachypodom			*trans*-ß-Caryophyllene (20.2%)<br>9-epi-*trans*-Caryophyllene (5.8%)<br>Caryophyllene oxide (10.8%)	23.43 µg/mL/*L. infantum* (promastigote)<br>>100.0 µg/mL/*L. infantum* (amastigote)	2.83	[253]
	Piper var brachypodon (Benth.) C. DC.			*trans*-ß-Caryophyllene (20.2%)<br>Bicyclogermacrene (8.5%)<br>Caryophyllene oxide (10.7%)	23.68 µg/mL/*L. infantum* (promastigote)<br>>100.0 µg/mL/*L. infantum* (amastigote)	2.65	[253]
	Piper claussenianum	Castelo	Fresh Leaves	(*E*)-Nerodiol (81.41%)<br>Linalool (5.20%)<br>γ-Muurolene (1.09%)	nd	nd	[254]
	Piper auritum	Cuba	Aerial parts	Safrole (86.91%)	29.1 µg/mL/*L. major* (promastigote)<br>63.3 µg/mL/*L. mexicana* (promastigote)<br>52.1 µg/mL/*L. braziliensis* (promastigote)	4.0 for *L. major*,<br>2.0 for *L. mexicana*,<br>2.0 for *L. braziliensis*	[255]

					12.8 µg/mL/*L. donovani* (promastigote)	and 8.0 for *L. donovani*	
Verbenaceae	*Lantana camara* L	Brazil/Ceará State	Leaves	(*E*)-Caryophyllene (23.75%)<br>Bicyclogermacrene (15.80%)<br>Germacrene D (11.73%)	72.31 µg/mL/*L.braziliensis* (promastigote)	nd	[255]
	Lippia alba	Santander/Bucaramanga	Aerial parts	Carvone (31.8%)<br>Limonene (29%)<br>Bicyclosesquiphellandrene (11.3%)	>100.0 µg/mL/*L. chagasi* (promastigote)	nd	[194]
	Lippia alba	Boyacá/Cubará		Carvone (49.4%)<br>Limonene (31.9%)	>100.0 µg/mL/*L. chagasi* (promastigote)	nd	[194]
	Lippia alba	Santander/Bucaramanga		Carvone (49.6%)<br>Limonene (28.8%)	>100.0 µg/mL/*L. chagasi* (promastigote)	nd	[194]
	Lippia alba	Cundinamarca/Cachipai		Carvone (47.6%)<br>Limonene (10.8%)	>100.0 µg/mL/*L. chagasi* (promastigote)	nd	[194]
	Lippia alba	Cundinamarca, Anolaima		Carvone (25.6%)<br>Limonene (18.2%)	>100.0 µg/mL/*L. chagasi* (promastigote)	nd	[194]
	Lippia alba	Tolima/Flandes		Carvone (48.3%)<br>Limonene (19.1%)	>100.0 µg/mL/*L. chagasi* (promastigote)	nd	[194]

	Lippia alba	Boívar/Colorado		Limonene (3.2%)<br>*trans-β*-Caryophyllene (7.3%)<br>Geranial (28.9%)	79.5 µg/mL/*L. chagasi* (promastigote)	nd	[194]
	Lippia alba	Santander/Bucaramanga		*trans-β*-Caryophyllene (6.6%)<br>Geranial (23.3%)	18.9 µg/mL/*L. chagasi* (promastigote)	nd	[194]
	Lippia alba	Bolívar/Turbaco		Limonene (3.2%)<br>*trans-β*-Caryophyllene (5.5%)<br>Geranial (26.7%)	91.5 µg/mL/*L. chagasi* (promastigote)	nd	[194]
	Lippia citriodora	Quindío/Armenia		Limonene (10.7%)<br>Neral (15.6%)<br>Geranial (18.9%)	77.9 µg/mL/*L. chagasi* (promastigote)	nd	[194]
	Lippia micromera	Cesar/Manaure		*p*-Cymene (13.1%)<br>Methyl thymyl ether (14.9%)<br>Thymol (29.1%)	51.8 µg/mL/*L. chagasi* (promastigote)	nd	[194]
	Lippia origanoides	Santander/Piedecuesta		*p*-Cymene (12.0%)<br>Carvacrol (46.2%)<br>γ-Terpinene (9.5%)	13.7 µg/mL/*L. chagasi* (promastigote)	nd	[194]
	Lippia origanoides	Santander/Jordán Sube		*p*-Cymene (13.9%)<br>Thymol (9.2%)<br>Carvacrol (36.5%)	63.2 µg/mL/*L. chagasi* (promastigote)	nd	[194]

	Lippia origanoides			*p*-Cymene (15.7%)<br>*tans-β*-caryophyllene (9.4%)	>100.0 µg/mL/*L. chagasi* (promastigote)	nd	[194]
	Lippia origanoides	Santander/Bucaramanga		Thymol (15.1%)<br>Carvacrol (38.8%)<br>γ-Terpinene (12.6%)	16.0 µg/mL/*L. chagasi* (promastigote)	nd	[194]
	Lippia origanoides	Santander/Los Santos		*p*-Cymene (11.2%)<br>*tans-β*-Caryophyllene (11.3%)	>100.0 µg/mL/*L. chagasi* (promastigote)	nd	[194]
	Lippia dulcis	Santander/Bucaramanga		α-Copaene (8.4%)<br>*tans-β*-Caryophyllene (10.4%)<br>α-Bisabolol (8.2%)	37.1 µg/mL/*L. chagasi* (promastigote)	nd	[194]
	Lippia sidoides	Brazil/Sergipe State	Leaves	*p*-Cymene (34.1%; 17.8)<br>Thymol (38.7%;6.0)<br>Carvacrol (0.6% 43.7%)<br>(Due different accessions)	74.1 and 54.8 µg/mL /*L. chagasi* (promastigote)<br>(Due different accessions)	nd	[256]
	Lippia sidoides		Aerial parts	Thymol<br>(78.37%)<br>ρ-cymene (6.32%)	44.38 µg/mL/*L. amazonensis* (promastigote)<br>34.4 µg/mL/*L. amazonensis* (amastigote)	4.91 for promastigote and 6.32 for amastigote	[238]
	Lippia gracilis		Leaves	Thymol (61.84%)<br>Carvacrol (48.92%)<br>(Two different genotypes)	86.32 and 77.26 µg/mL/*L. chagasi* (promastigote)<br>(Two different genotypes)	nd	[208]
	Lippia sidoides Cham.	Brazil/Piauí State		Thymol (68.3%)<br>*p*-Cymene (14.4%)	89.0 µg/mL/*L. chagasi* (promastigote)	nd	[246]

	Lippia sidoides comercial oil	nd	nd	Thymol (59.65%)<br>β-Caryophyllene (10.60%)	19.76 µg/mL/*L. chagasi* (promastigote)<br>5.07 µg/mL/*L. chagasi* (amastigote)	nd	[239]
	Lippia graveolens	nd	Aerial parts	*p*-Cymene (16.9%)<br>1,8-Cineole (6.6%)<br>Thymol (19.8%)	171.0 µg/mL/*L. infantum* (promastigote)	nd	[244]
Anacardiaceae	*Myracrodruon urundeuva* (Engl.) Fr. All	Brazil/Piauí State	Leaves	β-Myrcene (42.46%)<br>α-Myrcene (37.23%)<br>Caryophyllene (4.28%)	205.0 µg/mL/*L. amazonensis* (promastigote)<br>104.6 µg/mL/*L. amazonensis* (axenic amastigote)<br>44.5 µg/mL/*L. amazonensis* (intracellular amastigote)	12.3 for intracellular amastigote	[257]
		Brazil/Pernambuco State	Latex of fruits	β-Pinene (40.7%)<br>Terpinolene (28.3%)<br>α-Pinene (11.5%)	39.1 µg/mL/*L. amazonensis* (promastigote)	3.7	[258]
	Mangifera indica var. Espada		Latex of fruits	Terpinolene (73.6 %)<br>δ-3-Carene (5.7%)	23.0 µg/mL/*L. amazonensis* (promastigote)	6.9	[258]
Ranunculaceae	*Nigella sativa*	Northen Tunisia	Aerial parts	*p*-Cymene (53.1%)	10.68 µg/mL/*L. infantum* (promastigote)<br>13.25 µg/mL/*L. major* (promastigote)	20.67 for *L. infantum* and 16.66 for *L. major*	[178]
Rubiaceae	*Mitracarpus frigidus*	Brazil/Minas Gerais State		Linalool (29.9%)<br>Eugenol acetate (15.85%)<br>5-Hydroxy-isobornyl (8.41%)	47.2 µg/mL/*L. major* (promastigote)<br>89.7 µg/mL/*L. amazonensis* (promastigote)	nd	[259]

Amaranthaceae	*Chenopodium ambrosioides*	Cuba		nd	MIC= 27.8 mg/mL/*L. amazonensis* (promastigote)	nd	[241]
Pinaceae	*Pinus caribaea*	Pinar del Río		nd	MIC = 0.16 mg/mL/*L. amazonensis* (promastigote)	nd	[241]
Poaceae	*Cymbopogon citratus*	Angola/Luanda		Geranial (48.4%)<br>Neral (32.6%)<br>Myrcene (6.4%)	25.0 µg/mL/*L. infantum* (promastigote)<br>52.0 µg/mL/*L. tropica* (promastigote)<br>38.0 µg/mL/*L. tropica* (promastigote)	nd	[260]
	Cymbopogon citratus	nd		Myrcene (6.4%)<br>Neral (32.6%)<br>Geranial (48.4%)	25.0 µg/mL/*L. infantum* (promastigote)	nd	[244]
	Cymbopogon citratus (DC) Stapf.	Brazil/Piaui State	Leaves	Geranial (41.1%)<br>Neral (40.4%)	45.0 µg/mL/*L. chagasi* (promastigote)	nd	[246]
Cupressaceae	*Juniperus oxycedrus*	nd	Berries	α-Pinene (56.4%)	51.0 µg/mL/*L. infantum* (promastigote)	nd	[244]
	Juniperus oxycedrus	nd	Leaves	α-Pinene (76.4 %)	127.0 µg/mL/*L. infantum* (promastigote)	nd	[244]
Euphorbiaceae	*Croton leptostachyus*	Alvarado/Tolima	Leaves	nd	84.59 µg/mL/*L. panamensis* (amastigote)<br>7.39 µg/mL/*L. panamensis* (promastigote)<br>36.74 µg/mL/*L. braziliensis* (amastigote)<br>22.74 µg/mL/*L. braziliensis*	0.2 for *L. panamensis* amastigote,<br>2.24 for *L. panamensis* promastigote,	[204]

					(promastigote)	0.45 for *L. braziliensis* Amastigote and 0.73 for *L. braziliensis* promastigote	

REFERENCES – SUPLEMENTARY MATERIAL

[147] M. A. Andrade, C. D. Azevedo, F. N. Motta, et al., "Essential oils: In vitro activity against Leishmania amazonensis, cytotoxicity and chemical composition", *BMC Complement. Altern. Med.*, vol. 16, no. 1, pp. 1–8, 2016.

[156] A. O. Santos, T. Ueda-Nakamura, B. P. Dias Filho, V. F. Veiga Junior, A. C. Pinto, and C. V. Nakamura, "Effect of Brazilian copaiba oils on Leishmania amazonensis", *J. Ethnopharmacol.*, vol. 120, no. 2, pp. 204–208, 2008.

[160] D. C. Soares, N. A. Portella, M. F. D. S. Ramos, A. C. Siani, and E. M. Saraiva, "Trans-β-caryophyllene: An effective antileishmanial compound found in commercial copaiba Oil (Copaifera spp.)", *Evidence-based Complement. Altern. Med.*, vol. 2013, 2013.

[162] Y. Tariku, A. Hymete, A. Hailu, and J. Rohloff, "Essential-oil composition, antileishmanial, and toxicity study of Artemisia abyssinica and Satureja punctata ssp. punctata from Ethiopia", *Chem. Biodivers.*, vol. 7, no. 4, pp. 1009–1018, 2010.

[163] V. D. da Silva, F. Almeida-Souza, A. M. Teles, et al., "Chemical composition of Ocimum canum Sims. essential oil and the antimicrobial, antiprotozoal and ultrastructural alterations it induces in Leishmania amazonensis promastigotes", *Ind. Crops Prod.*, vol. 119, no. March, pp. 201–208, 2018.

[173] K. A. F. Rodrigues, L. V. Amorim, C. N. Dias, et al., "*Syzygium cumini* (L.) Skeels essential oil and its major constituent α-pinene exhibit anti-Leishmania activity through immunomodulation *in vitro*", *J. Ethnopharmacol.*, vol. 160, pp. 32–40, 2015.

[175] A. Bouyahya, A. Et-Touys, J. Abrini, et al., "*Lavandula stoechas* essential oil from Morocco as novel source of antileishmanial, antibacterial and antioxidant activities", *Biocatal. Agric. Biotechnol.*, vol. 12, no. July, pp. 179–184, 2017.

[178] R. Essid, F. Z. Rahali, K. Msaada, et al., "Antileishmanial and cytotoxic potential of essential oils from medicinal plants in Northern Tunisia", *Ind. Crops Prod.*, vol. 77, pp. 795–802, 2015.

[194] P. Escobar, S. M. Leal, L. V. Herrera, J. R. Martinez, and E. Stashenko, "Chemical composition and antiprotozoal activities of Colombian *Lippia spp* essential oils and their major components", *Mem. Inst. Oswaldo Cruz*, vol. 105, no. 2, pp. 184–190, 2010.

[204] F. L. Neira, E. Stashenko, and P. Escobar, “Actividad antiparasitaria de extractos de plantas colombianas de la familia Euphorbiaceae”, *Rev. la Univ. Ind. Santander.*, vol. 46, no. 1, pp. 15–22, 2014.

[208] J. O. de Melo, T. A. Bitencourt, A. L. Fachin, et al., “Antidermatophytic and antileishmanial activities of essential oils from *Lippia gracilis* Schauer genotypes”, *Acta Trop.*, vol. 128, no. 1, pp. 110–115, 2013.

[237] J. R. A. Silva, D. M. F. Carmo, E. M. Reis, et al., “Chemical and biological evaluation of essential oils with economic value from lauraceae species”, *J. Braz. Chem. Soc.*, vol. 20, no. 6, pp. 1071–1076, 2009.

[238] M. D. Medeiros, A. C. Silva, A. M. Citó, et al., “*In vitro* antileishmanial activity and cytotoxicity of essential oil from *Lippia sidoide*s Cham”, *Parasitol. Int.*, vol. 60, no. 3, pp. 237–241, 2011.

[239] H. C. R. M. F. C. M. Rondon; C. M. L. Bevilaqua, M. P. Accioly, S. M. Morais, H. F. Andrade-Júnior, C. A. Carvalho, J. C. Lima, “*In vitro* efcacy of *Coriandrum sativum*, *Lippia sidoides* and *Copaifera reticulata* against *Leishmania chagasi*”, *Rev. Bras. Parasitol. Vet., Jaboticabal*, vol. 21, no. 3, pp. 185–191.

[240] J. Mikus, M. Harkenthal, D. Steverding, and J. Reichling, “*In vitro* effect of essential oils and isolated mono-and sesquiterpenes on *Leishmania major* and *Trypanosoma brucei*”, *Planta Med.*, vol. 66, pp. 66–68, 2000.

[241] L. M. Fidalgo, I. S. Ramos, A. M. M. Álvarez, N. G. Lorente, R. S. Lizama, and J. A. Payrol, “Propiedades antiprotozoarias de aceites esenciales extraídos de plantas cubanas”, *Rev. Cubana Med. Trop.*, vol. 56, no. 3, pp. 230–233, 2004.

[242] M. García, R. Scull, P. Satyal, W. N. Setzer, and L. Monzote, “Chemical Characterization, Antileishmanial Activity, and Cytotoxicity Effects of the Essential Oil from Leaves of *Pluchea carolinensis* (Jacq.) G. Don. (Asteraceae)”, *Phyther. Res.*, vol. 31, no. 9, pp. 1419–1426, 2017.

[243] N. A. Parreira, L. G. Magalhães, D. R. Morais, et al., “Antiprotozoal, Schistosomicidal, and Antimicrobial Activities of the Essential Oil from the Leaves of *Baccharis dracunculifolia*”, *Chem. Biodivers.*, vol. 7, no. 4, pp. 993–1001, 2010.

[244] M. Machado, G. Santoro, M. C. Sousa, L. Salgueiro, and C. Cavaleiro, “Activity of essential oils on the growth of *Leishmania infantum* promastigotes”, *Flavour Fragr. J.*, vol. 25, no. 3, pp. 156–160, 2010.

[245] C. A. T. Siqueira, J. Oliani, A. Sartoratto, et al., “Chemical constituents of the volatile oil from leaves of *Annona coriacea* and *in vitro* antiprotozoal activity”, *Brazilian J. Pharmacogn.*, vol. 21, no. 1, pp. 33–40, 2011.

[246] V. C. S. Oliveira, D. M. S. Moura, J. A. D. Lopes, P. P. De Andrade, N. H. Da Silva, and R. C. B. Q. Figueiredo, “Effects of essential oils from *Cymbopogon citratus* (DC) Stapf., *Lippia sidoides* Cham., and *Ocimum gratissimum* L. on growth and ultrastructure of *Leishmania chagasi* promastigotes”, *Parasitol. Res.*, vol. 104, no. 5, pp. 1053–1059, 2009.

[247] M. Machado, A. M. Dinis, M. Snatos-Rosa, et al., “Activity of *Thymus capitellatus* volatile extract, 1,8-cineole and borneol against Leishmania species”, *Vet. Parasitol.*, vol. 200, no. 1–2, pp. 39–49, 2014.

[248] C. N. Dias, K. A. Rodrigues, F. A. Carvalho, et al., “Molluscicidal and leishmanicidal activity of the leaf essential oil of *Syzygium cumini* (L.) Skeels from Brasil”, *Chem. Biodivers.*, vol. 10, pp. 1133–1141, 2013.

[249] K. Z. Bernuci, C. C. Iwanaga, C. M. Fernandez-Andrade, et al., “Evaluation of chemical composition and antileishmanial and antituberculosis activities of essential oils of Piper species”, *Molecules*, vol. 21, no. 12, p. 12, 2016.

[250] L. F. Ceole, M. D. G. Cardoso, and M. J. Soares, “Nerolidol, the main constituent of *Piper aduncum* essential oil, has anti-*Leishmania braziliensis* activity”, *Parasitology*, vol. 144, no. 9, pp. 1–12, 2017.

[251] M. L. A. S. V. R. Esperandim, D. S. Ferreira, K. C. S. Rezende, L. G. Magalhães, J. M. Souza, P. M. Pauletti, A. H. Januário, R. S. Laurentz, J. K. Bastos, G. V. Símaro, and W. R. Cunha, “Chemical composition and antiprotozoal activities of Colombian *Lippia spp* essential oils and their major components”, *Planta Med.*, vol. 79, p. 1653, 2013.

[252] Y. Gutiérrez, R. Montes, R. Scull, et al., “Chemodiversity Associated with Cytotoxicity and Antimicrobial Activity of *Piper aduncum var. ossanum*”, *Chem. Biodivers.*, vol. 13, no. 12, pp. 1715–1719, 2016.

[253] S. M. Leal, N. Pino, E. E. Stashenko, J. R. Martínez, and P. Escobar, “Antiprotozoal activity of essential oils derived from *Piper spp*. grown in Colombia”, *J. Essent. Oil Res.*, vol. 25, no. 6, pp. 512–519, 2013.

[254] A. M. Marques, A. L. S. B. Barreto, J. A. da R. Curvelo, M. T. V. Romanos, R. M. d. A. Soares, and M. A. C. Kaplan, “Antileishmanial activity of nerolidol-rich essential oil from *Piper claussenianum*”, *Brazilian J. Pharmacogn.*, vol. 21, no. 5, pp. 908–914, 2011.

[255] L. Monzote, M. García, A. M. Montalvo, R. Scull, and M. Miranda, “Chemistry, cytotoxicity and antileishmanial activity of the essential oil from *Piper auritum*”, *Mem. Inst. Oswaldo Cruz*, vol. 105, no. 2, pp. 168–173, 2010.

[256] P. A. Farias-Junior, M. C. Rios, T. A. Moura, et al., “Leishmanicidal activity of carvacrol-rich essential oil from *Lippia sidoides* Cham.”, *Biol. Res.*, vol. 45, no. 4, pp. 399–402, 2012.

[257] C. E. Carvalho, E. P. Sobrinho-Junior, L. M. Brito, et al., “Anti-*Leishmania* activity of essential oil of *Myracrodruon urundeuva* (Engl.) Fr. All.: Composition, cytotoxity and possible mechanisms of action”, *Exp. Parasitol.*, vol. 175, pp. 59–67, 2017.

[258] E. H. Ramos, M. M. Moraes, L. L. Nerys, et al., “Chemical composition, leishmanicidal and cytotoxic activities of the essential oils from *Mangifera indica* L. *var. Rosa* and *Espada*.”, *Biomed Res. Int.*, vol. 2014, p. 734946, 2014.

[259] L. Monzote, M. García, A. M. Montalvo, R. Scull, M. Miranda, and J. A. Payrol, “*In Vitro* Activity of an Essential Oil against *Leishmania donovani*”, *Phyther. Res.*, vol. 22, no. April 2008, pp. 557–559, 2009.

[260] M. Machado et al., “Monoterpenic aldehydes as potential anti-Leishmania agents: Activity of *Cymbopogon citratus* and citral on *L. infantum*, *L. tropica* and *L. major*”, *Exp. Parasitol.*, vol. 130, no. 3, pp. 223–231, 2012.

[261] M. D. Medeiros, A.C. Silva A. M. Citó, et al., "*In vitro* antileishmanial activity and cytotoxicity of essential oil from *Lippia sidoides Cham*.", *Parasitol. Int.* vol. 60, pp. 237–241, 2011.

[262] V. D. Zheljazkov, C. L. Cantrell, B. Tekwani, and S. I. Khan. “Content, Composition, and Bioactivity of the Essential Oils of Three Basil Genotypes as a Function of Harvesting”, *J. Agric. Food Chem,* vol. 56, pp. 380–385, 2008.
